# Abstracts From 3rd Annual Meeting of the Connective Tissue Oncology Society

**DOI:** 10.1080/13577149778317

**Published:** 1997-12


*Sarcoma* (1998) **1**, 193–226

CARFAX

### Abstracts from 3rd Annual Meeting of the Connective Tissue Oncology Society

### Proffered paper sessions

### Involvement of bone or neurovascular structures by soft tissue sarcoma at MRI as prognostic factors

D. M. Panicek, S. D. Go, J. H. Healey, D. Leung, M. F. Brennan & J. J. Lewis
(*Memorial Sloan-Kettering Cancer Center, New York, NY 10021, USA*)

*Purpose.* To evaluate whether involvement of bone or major neurovascular structures by soft tissue sarcoma as determined from magnetic resonance images (MRI) is predictive of local recurrence (LR), distant metastasis (DM) or disease-specific survival (DSS).
*Patients and methods.* Pre-operative MRI of 46 consecutive patients with soft tissue sarcoma (from the Radiologic Diagnostic Oncology Group musculoskeletal tumor study) were reviewed by two radiologists for the presence of bone, major vessel and major nerve involvement by the tumor, and for the closest distance between the tumor and these structures. Findings were correlated with subsequent LR, DM and DSS after surgery with curative intent and adjuvant chemotherapy and/or radiotherapy (RT). The primary tumors were predominantly in the lower extremity (74%); 96% were deep, 76% were high-grade and 89% were larger than 5 cm.
*Results.* Radiologically, 26% of tumors invaded bone, 11% encased a major vessel and 15% encased a major nerve. For patients with and without bone invasion, 0% versus 21% ($p = 0.16$) had LR, 67% versus 53% ($p = 0.7$) had DM and 75% versus 35% ($p = 0.02$) died of disease. For patients with and without vessel encasement, 20% versus 15% ($p > 0.9$) had LR, 75% versus 54% ($p = 0.6$) had DM and 60% versus 44% ($p = 0.7$) died of disease. For patients with and without nerve encasement, 14% versus 15% ($p > 0.9$) had LR, 50% versus 57% ($p = 0.8$) had DM and 57% versus 44% ($p = 0.7$) died of disease. No significant difference in LR, DM or DSS was found between lesions located more than 1 cm from bone, vessel or nerve and those that contacted those structures.
*Conclusion.* MRI demonstration of bone invasion was significantly predictive of decreased DSS. Appropriate tailoring of surgical procedures based on MRI evidence for bone or neurovascular involvement appears to have overshadowed any other prognostic significance of those findings for LR, DM or DSS.

### Chondrosarcoma of the phalanx; a locally aggressive lesion

J. V. M. G. Bovée,[1] R. O. Van Der Heul,[1] A. H. M. Taminiau[2] & P. C. W. Hogendoorn[1]

([1]*Departments of Pathology &* [2]*Orthopaedic Surgery, Leiden University Medical Center, The Netherlands*)

*Introduction.* Chondrosarcomas are the second most frequent primary malignant bone tumors. Enchondromas are the most common primary benign bone tumors of the hand, predominantly located in the phalanges. On the contrary, chondrosarcomas with phalangeal localization are rather uncommon. The clinicobiological behavior of these tumors is poorly documented in the literature.
*Methods.* Thirty-five cases of chondrosarcoma of the phalanx in the period 1960–1994 were retrieved from the files of the Netherlands Committee on Bone Tumors, containing more than 11 000 cases of bone tumors documented by clinical history, X-rays and histology. Histological slides and radiographs were reviewed. Histological grading was done according to Evans. Follow-up was obtained from treating physicians. Additionally, an analysis of 65 cases of chondrosarcoma of the phalanx retrieved from the literature (1934–1997) was performed.
*Results.* From the 35 cases, two were excluded by reviewing the slides of the resected specimen. One case turned out to be localized in the metacarpus and one was reclassified as chondroblastic osteosarcoma. Treatment varied from localized therapy (excochleation, curettage or local resection) for 12 patients to exarticulation/amputation at different levels for 20 patients. From one case the exact therapy could not be documented. An average follow-up of 81 months could be retrieved for 24 patients. Nine cases were lost to follow-up. None of the patients developed metastases or died as a result of the tumor. Nine of them had a local recurrence with an average interval of 3 years, and three patients had a second recurrence. Nine of 11 patients who received additional therapy for residue or recurrent tumor received local therapy as first treatment. Survival data could be obtained for 26 patients; the average survival was 140 months. In the summarized literature, the average follow-up time was 65 months for 57 patients. One case documented in the literature dating from 1942 developed metastases. The slides of this case were reviewed and the reported diagnosis was confirmed. Twelve patients in the literature had a local recurrence.
*Conclusion.* Chondrosarcoma of the phalanx is a lesion with a histologically and radiographically malignant appearance. However, the biological behavior is locally destructive only and development of metastases is extremely rare. Therefore, a conservative surgical approach seems to be justified.

**[Goletz *et al.*, see page 210]**

### Late effects of therapy in patients with localized primary orbital rhabdomyosarcoma: a report from the Intergroup Rhabdomyosarcoma Study (IRS)-III, 1984–1991

R. B. Raney, J. Kollath, J. Anderson, M. Wharam & H. Maurer for the IRS Group of the Children's Cancer Group and Pediatric Oncology Group
(*The University of Texas, MD Anderson Cancer Center, Houston, TX 77030, USA*)

We reviewed sequelae in 52 patients treated on IRS-III for

1357-714X/97/030193-34 © 1997 Journals Oxford Ltd orbital rhabdomyosarcoma (RMS). Six patients had the globe removed, three after local recurrence and one each for initial therapy, treatment complications or unknown cause. Forty-six patients (88%) continue with the globe in place. Sixty-eight per cent had reduced visual acuity in the affected eye, including four with unilateral blindness. Seventy-four per cent had a unilateral cataract, and 64% of them underwent cataract extraction. The affected eye was dry in 39% of the patients and painful in 20%; 27% had unilateral ptosis. Chronic conjunctivitis or keratitis was noted in approximately 20% of patients, but only 7% had corneal ulceration, and 11% had increased tearing. Only one patient had retinal detachment. A hypoplastic orbit was reported in 61% of the patients, but only 21% had significant enophthalmos. Twenty-one per cent of the patients were referred for plastic/craniofacial surgical reconstruction. Four patients (9%) received growth hormone therapy. Thirteen of 14 girls had a normal menstrual pattern, and three had established fertility. We conclude that the most common late sequelae of therapy for orbital RMS are decreased visual acuity, unilateral cataract, hypoplastic orbit and dry eye. Serious post-treatment problems, such as corneal ulceration or growth failure requiring hormone treatment, are unusual and may become even less frequent with lower radiation therapy doses and improved techniques.

**[De Paoli *et al.*, see page 208]**

### Localized high-grade soft tissue sarcomas of the extremities in adults: preliminary results of the Italian cooperative study

P. Picci, S. Frustaci, A. De Paoli, G. Pignatti, H. Zmerly, A. Azzarelli, A. Comandone, A. Buonadonna, P. Olmi, V. Ippolito, E. Barbieri, G. Apice, S. Ferrari, G. Bacci & F. Gherlinzoni
(*Italian Cooperative Group, Instituti Ortopedici Rizzoli, Bologna, Italy*)

The efficacy of adjuvant chemotherapy in soft tissue sarcomas is still an open question, with some recent studies, including a large meta-analysis, indicating some benefits especially for tumors located in the extremities. The contradictory results reported in other randomized studies performed in the past may also be due to the heterogeneity of the tumors included and/or the mild chemotherapy employed in the treatment arms. Keeping these facts in mind, a new randomized study was activated in Italy in June 1992. The inclusion criteria were very strict and only those sarcomas which were localized, high grade (3 or 4), spindle or pleomorphic, deeply seated, larger than 5 cm (also smaller if presenting as a local recurrence), located in an extremity or girdle, in patients ranging in age between 18 and 65 years, entered the study. After local treatment and stratification for size and presentation (primary or recurrent), patients were randomized to receive, or not, a very intensive adjuvant chemotherapy consisting of five cycles of epirubicin (120 mg $m^{-2}$ in 2 days), ifosfamide (9 g $m^{-2}$ in 5 days) with mesna and G-CSF (300 mg/day sc day 8/15), q 3 weeks. An interim analysis was performed after half of the above mentioned patients had been randomized according to the protocol, in October 1996, with 104 patients in the study. Fifty-three and 51 patients were randomized to chemotherapy or control, respectively. This analysis revealed a statistically significant difference both for disease-free survival and overall survival ($p = 0.001$ and $p = 0.007$, respectively). The analysis was repeated in May 1997. After a median follow-up of 24 months (range 5–57 months), 47 patients (45%) relapsed: 19 in the treatment arm and 28 in the control arm. Furthermore, 27 patients (26%) died: 9 in the treatment arm and 18 in the control arm. First site of relapse was local in 7 patients, distant in 38 and both in 2. The statistical analysis again shows a difference between the two treatment groups at the level of $p = 0.001$ for disease-free survival and at the level of $p = 0.005$ for overall survival. Longer follow-up is mandatory to confirm these differences but the high level of statistical evidence persuaded us to stop the accrual of new patients.

### Induction of apoptosis in a leiomyosarcoma cell line by adenoviral-mediated over-expression of the transcription factor E2F-1

K. K. Hunt, T.-J. Liu, B. Feig, P. W. T. Pisters, R. E. Pollock, D. Yu & M.-C. Hung
(*MD Anderson Cancer Center, Houston, TX 77030, USA*)

*Introduction.* Surgery continues to be the primary treatment modality for patients with leiomyosarcomas. These tumors are often resistant to chemotherapy, radiation therapy and other treatment modalities. We sought to investigate a novel gene therapy approach for the induction of apoptosis, or programmed cell death, in a leiomyosarcoma cell line using a recombinant adenovirus coding for the transcription factor E2F-1.
*Methods.* A leiomyosarcoma cell line (SKLMS) with a known p53 mutation was studied in addition to a stable transfectant which expresses will-type p53 (SKLMS-37). Cells were mock-infected (control), infected with a recombinant adenovirus without a foreign transgene (AdV) or a recombinant adenovirus containing the transcription factor E2F-1 (AdVE2F) at a multiplicity of infection of 100. Cells were assessed for evidence of apoptotic cell death using assessment of morphologic changes, a commercially available 'cell death' ELISA and flow cytometry analysis (FACS) after propidium iodide staining. Protein expression was assessed using Western blot analysis.
*Results.* Over-expression of the transcription factor E2F-1 was verified in all of the cell lines tested 48 h after infection. Cells treated with AdVE2F demonstrated morphologic changes consistent with apoptosis within 72 h of infection. By day 6, a significant population of the cells had undergone apoptotic cell death as measured by FACS analysis. The results are listed below.

| | SKLMS Parental | SKLMS-37 Clone |
|---|---|---|
| Control | 12% | 9% |
| AdV | 14% | 9% |
| AdVE2F | 38% | 23% |

The quantitative assessment of a subdiploid population (apoptotic cells) by FACS analysis was verified using the 'cell death' ELISA assay. We noted more significant apoptosis in the mutant p53 parental SKLMS cell line as compared with the p53 transfected SKLMS-37 clone.
*Conclusions.* Over-expression of the transcription factor E2F-1 in a leiomyosarcoma cell line results in apoptotic cell death in a significant number of the cell population. These results seem to be more dramatic in the parental mutant p53 cell line. These results have implications for potential therapy. Additional cell lines will need to be tested and an in vivo strategy will also be developed for treatment of human leiomyosarcoma grown in nude mice.

**[Feig *et al.*, see page 2120]**
**[Weibolt *et al.*, see page 225]**
**[Benassi *et al.*, see page 201]**

### Isolation of osteosarcoma-associated homozygously deleted DNA sequences using representational difference analysis

A. Simons,[1] M. Schepens,[1] A. Forus,[2] & A. Geurts van Kessel[1]
*([1]Department of Human Genetics, University Hospital Nijmegen, The Netherlands & [2]Department of Tumor Biology, Norwegian Radium Hospital, Oslo, Norway)*

Representational difference analysis (RDA) allows for the direct isolation of differences between tumor DNA and normal DNA through a combination of subtractive hybridization and polymerase chain reaction (PCR) amplification. Previously, we were able to isolate osteosarcoma-associated amplified DNA fragments using this technique. In this paper, we present the isolation of DNA fragments that are homozygously deleted in an osteosarcoma. Using tumor (xenograft) DNA as driver and a mixture of DNAs from 15 normal individuals as tester, several DNA fragments (difference products; DP) were obtained after three rounds of subtraction and amplification. Southern blot analysis revealed that some of the difference products represented polymorphisms. Other fragments, however, showed signals only in the normal DNA (tester) and are, thus, absent (deleted) in the xenograft (driver). These results were confirmed by PCR analysis of normal- and xenograft DNA using DP-derived primers. Subsequent somatic cell hybrid analysis disclosed the chromosomal origin of the isolated tumor-deleted fragments. Further analysis of these fragments may lead to the isolation of a (novel) tumor-suppressor gene(s) involved in the development of osteosarcomas.

**[Graeven *et al.*, see page 211]**

### Isolated single lung perfusion with adriamycin for unresectable sarcomatous metastases

J. B. Putnam Jr, T. Madden, H. T. Tran & R. S. Benjamin
*(The Sarcoma Center, University of Texas, MD Anderson Cancer Center, Houston, TX 77030, USA)*

We have hypothesized that isolated single lung perfusion (ISLP) will safely deliver high levels of drug to lung and tumor, minimize systemic toxicity and enhance survival. To begin to test this hypothesis, we conducted a phase I study of ISLP in 14 patients with isolated unresectable lung metastases from soft tissue sarcomas between January 1995 and April 1996. All patients had exceeded systemic tolerance for effective chemotherapeutic agents including adriamycin and ifosfamide. Two patients subsequently had resectable PM and were not perfused. Twelve eligible patients underwent thoracotomy and ISLP. The pulmonary artery (PA) and pulmonary veins (PV) were dissected, isolated and clamped. ISLP via the PA over 20 min was performed with adriamycin at 60 mg m$^{-2}$ ($n = 8$; 200 mg m$^{-1}$; group I) or 75 mg m$^{-2}$ ($n = 4$; 250 mg ml$^{-1}$; group II). Effluent was drained from the PV and not recirculated. Higher drug levels were obtained in lung tissues (median 592 $\mu$g g$^{-1}$) compared to tumor (median 153 $\mu$g g$^{-1}$). No intra-operative complications occurred. Two patients from group II developed acute drug-related lung injury and one died. One patient died from tumor embolus (group I, not ISLP-related) (mortality 16.6%, 2/12). Median survival exceeds 18 months. Radiographs from nine evaluable patients were reviewed for treatment response and compared to PM in the untreated lung. Regression of ipsilateral PM growth was observed in 5/9 evaluable patients with one major response and four patients with temporarily stable disease. Late follow-up revealed 50% decrease in ventilation and perfusion of the ISLP lung ($n = 3$) compared to reductions of FEV1 and FVC of only 20% ($n = 6$). We conclude that: (1) ISLP with adriamycin may be performed safely at 60 mg m$^{-2}$ (200 mg l$^{-1}$) with moderate late toxicity, (2) ISLP delivers higher drug levels to the lung than to tumor, (3) acute toxicity reflects drug concentration and (4) late toxicity reflects gradual attenuation of ventilation and perfusion after ISLP. Further studies are warranted to refine the therapeutic effectiveness of this therapy.

**[Hieken *et al.*, see page 212]**

### Multi-modal therapy combined with aggressive surgery for vessel-invasive soft tissue sarcoma

P. Hohenberger,[1] C. Kettelhack,[1] P. Reichardt[2] & P. M. Schlag[1]

*([1]Division of Surgical and [2]Medical Oncology, Robert-Rössle Hospital, Virchow Klinikum at the Humboldt University, D-13125 Berlin, Germany)*

*Problem and approach.* Soft tissue sarcoma infiltrating neurovascular bundles is a major challenge to achieving limb-saving surgery. Nineteen patients underwent pre-operative treatment and extended sarcoma resection with reconstruction of major vessels by a vein or an allograft. The impact of this strategy on limb-salvage rate, local disease-free survival and overall survival was analyzed.
*Patients and treatment.* There were nineteen patients (12 male, 6 female 22–65 years) with soft tissue sarcoma invading to neurovascular bundles. Eight patients presented with recurrent tumors and two of them had a recurrence within a radiation field. Histological subtypes were MFH ($n = 5$), liposarcoma ($n = 4$), leiomyosarcoma ($n = 5$), synovial sarcoma ($n = 2$), alveolar type ($n = 2$) and mesenchymoma ($n = 1$). Pre-operatively, patients were treated by either isolated limb perfusion with TNF$\alpha$ + melphalan, or systemic high-dose ifosfamide/epirubicin, or hyperfractionated radiotherapy (66 Gy). In six patients, a myocutaneous flap was required for soft tissue coverage after vessel resection and reconstruction.
*Results.* Limb salvage was possible in 18/19 patients The median local recurrence-free survival was 51 months. Three local relapses were detected one of which could be resected again for cure. Twelve out of 19 patients developed distant metastases with median survival free from metastases of 23 months. The overall median survival time is 48 months.
*Conclusions.* Extended sarcoma resection after preoperative aggressive multi-modal therapy achieves long-term local recurrence-free survival and limb salvage. However, distant metastases mainly in the lung are observed in the overwhelming majority of patients. Thus, amputation of extremity sarcoma can hardly be justified even in cases of tumor invasion to major vessels, but the problem of systemic spread remains unsolved.

**[Lewis *et al.*, see page 215]**

**Impact of multi-disciplinary management on outcome in adult soft tissue sarcomas of the chest wall**

B. O'Sullivan, C. Catton, T. Panzarella, A. Davis, M. Johnston, R. Bell & J. Wunder
*(Princess Margaret Hospital, University of Toronto, Ontario, Canada CM5G 2M9)*

The prognosis of soft tissue sarcomas (STS) in varying anatomic locations differs although the explanation for this may be multi-factorial. For example, lesions of the trunk are reputed to have a less favorable outcome compared to the more common lesions in the extremities where the modern principles of adult STS treatment, including multi-disciplinary management, were largely developed. The present study was undertaken to assess the impact of the introduction of a comprehensive multi-disciplinary management approach on the local control, metastatic failure and cause-specific survival in a non-extremity subsite (adult cases of non-metastatic STS of the chest wall) registered at our center from January 1980 until December 1994. In 1987, a rigorous and consistent approach to assessment, treatment and audit was introduced with the goal of improving outcome for STS. During the period, 66 non-metastatic lesions of the chest wall were registered from a total database of 1117 STS cases overall. The median follow-up of the chest wall lesions was 5.2 years (range 0.34–16.2 years). A variety of histopathologic types were seen, with malignant fibrous histiocytoma the most frequent (24 or 36.4%). Size was $\leq 5$ cm in 25 (38%), 5–10 cm in 27 (41%) and $> 10$ cm in 14 (21%). Tumor grade was high in 50 and low in 16; 25 were superficial, while 41 arose deep to the investing fascia. Treatment consisted of chemotherapy in 14, surgery in 60, while 47 received radiotherapy. The 5-year actuarial local relapse-free rate was 78%, cause-specific survival 72% and distant relapse-free rate 68%. The most apparent prognostic factors for time to cause-specific death were performance status ($p = 0.001$), lesion size ($p < 0.0001$) and local extension ($p < 0.0001$). Patients treated between January 1980 and June 1987 were compared to those treated in the second half of the study (July 1987 and December 1994), when management changed to a consistent multi-disciplinary approach to assessment and treatment. The 5-year actuarial local relapse-free rate was 89% in the later period compared to 71% in the early period, despite an equal distribution of prognostic factors for both groups. Similarly, the 5-year metastatic failure-free rate improved (82% versus 56%, $p < 0.05$), as did 5-year cause-specific survival (84% versus 60%, $p = 0.038$). These results demonstrate that high rates of control and survival should be expected in STS of the chest wall and at a level similar to that for extremities. In addition they support a policy of referral of these rare tumors to a multi-disciplinary setting for definitive management as this strategy is associated with an improvement in tumor outcome.

**Initial results with high-dose-rate brachytherapy for soft tissue sarcomas**

R. L. Crownover, K. E. Marks, R. J. Zehr, E. J. Lee, G. F. Muschler, M. J. Joyce, P. Lavertu & G. T. Budd
*(The Cleveland Clinic Foundation, Cleveland, OH 44195-5213, USA)*

Radiotherapy is frequently employed in combination with surgery for the management of soft tissue sarcomas. For selected cases, interstitial brachytherapy is an effective method of applying localized radiation. Traditional brachytherapy is delivered with low-dose-rate (LDR) sources which require hospitalization in a shielded room for several days and incidental radiation exposure to hospital staff. High-dose-rate (HDR) devices have recently gained popularity with radiation oncologists due to the elimination of staff exposure and the ability to treat on an outpatient basis. The biologic equivalence of HDR and LDR treatment schedules remains controversial. Twelve patients with soft tissue sarcomas have received treatment using HDR techniques at our institution. Eight patients received definitive HDR brachytherapy as the sole radiation treatment, and four received HDR brachytherapy as a boost treatment along with external beam irradiation. Initial results for 10 patients having follow-up greater than 1 year show 100% local control without excessive acute or late radiation sequelae. In addition, our experience with similar implants employed to manage other tumor types will be discussed. Our preliminary conclusion is that HDR brachytherapy is an attractive alternative to LDR techniques for soft tissue sarcomas.

**Complete surgery of lung metastases combined with chemotherapy in 31 adult soft tissue sarcomas: long-term results**

P. Casali,[1] U. Pastorino,[2] R. Bertulli,[1] A. Azzarelli,[1] V. Quagliuolo,[3] S. Fissi,[1] L. Balzarini,[1] R. Kenda[1], L. Lozza,[1] M. Mastore,[4] A. Laffranchi,[1] A. Valente,[1] C. Catania[1] & A. Santoro[3]

*([1]Istituto Nazionale Tumori, Milan, Italy; [2]Royal Brompton Hospital, London, UK, [3]Istituto Clinico Humanitas, Milan & [4]Ospedale Civile, Desio, Italy)*

Surgery is standard treatment for operable, isolated lung metastases from adult soft tissue sarcomas. The role of chemotherapy in this patient group is still unassessed. In 1988, a prospective study was undertaken at the Istituto Nazionale Tumori, Milan, Italy, on the EID regimen (epirubicin 90 mg m$^{-2}$ + ifosfamide 7500 mg m$^{-2}$ in 3 days with mesna + dacarbazine 900 mg m$^{-2}$ in 3 days), which was combined with surgery for lung metastases in all operable patients suffering from adult non-small cell soft tissue sarcomas with no extrapulmonary metastatic sites. Fifty-two patients with isolated lung metastases were enrolled; 17 of them also had local disease. Completely resectable patients were given three courses of chemotherapy before surgery for lung metastases; otherwise, up to six courses were given, followed by surgery, if feasible. Thirty-one patients (60% of the series) were able to undergo a complete excision of all detectable lung nodules through a median sternotomy ($\pm$ adequate surgery for local disease in eight of them). Median follow-up is as high as 75 months. The response rate to the EID regimen was 52% (95% CI: 38–66%), thus demonstrating that the chemotherapy employed in this study was a highly 'active' one. While unresectability proved to be a poor prognostic factor, correlating with a median survival of only 10 months in the 21 inoperable patients, the 31 surgically treated patients had a median survival of 45 months. Their 7-year actuarial disease-free and overall survival reached a plateau, at 22 and 26%, respectively. Seven patients are therefore long-term disease-free survivors: all had a previous free interval higher than 10 months, all were chemosensitive (4/7 patients had a pathologically complete response, 2/7 a partial response, 1/7 a minor response); the number of lesions was $\leq 5$ in 5/7 patients as assessed at comuted tomography (CT) -scan before chemotherapy, in 7/7 as assessed at surgical exploration after cytoreduction. One patient was converted to resectability after chemotherapy. At a long actual follow-up, this series confirms that 'cure' seems to be achievable through surgery of lung metastases in some metastatic adult soft tissue sarcomas with relatively favourable prognostic factors. A plateau level in the range of 25% would not suggest that chemotherapy may have significantly affected the cure rate in this series, even though cyto-reduction might benefit the occasional patient with worse prognostic factors. However, relatively long 'palliation' was achieved in incurable patients, as demonstrated by the median overall survival in excess of 3 years. Chemotherapy might have contributed to the palliative effect, but it is still to be demonstrated that this treatment strategy is superior to delaying medical treatment until relapse. Currently, the high number of patients previously treated with adjuvant chemotherapy makes it extremely difficult to carry out controlled trials in this patient population, comparing surgery plus chemotherapy in chemotherapy-naive patients versus surgery without chemotherapy. The physician is therefore left with the task of tailoring the decision of whether to add chemotherapy to surgery according to known prognostic factors, even by sharing the uncertainty with the patient, as it often occurs in rare tumors. New approaches to clinical research are needed, as are new treatment strategies.

### Regional induction chemotherapy and surgical resection for large or unresectable soft tissue sarcomas: evaluation of tumor necrosis, disease-free survival and local recurrence—is radiation therapy necessary for good responders?

R. Henshaw, C. Rubert, D. Priebat, D. Perry, B. Shmookler & M. Malawer
(*Washington Cancer Institute at The Washington Hospital Center, Washington, DC 20010, USA*)

*Introduction.* Treatment of large and/or unresectable soft tissue sarcomas (STS) of the extremities remains a major challenge. Based upon experience with primary bone sarcomas, we hypothesized that induction chemotherapy would permit limb-sparing surgery by inducing tumor necrosis. In addition, we postulated that local control can be obtained for good responders without radiation therapy (RT).
*Strategy.* Twenty-four patients presenting with large and/or unresectable intermediate to high-grade STS of the extremities were prospectively enrolled in a neo-adjuvant (induction) protocol consisting of two cycles of intra-arterial (regional) cis-platin and intravenous adriamycin chemotherapy followed by limb-sparing surgery. Only patients who were deemed unresectable or of large tumor volume were treated in this study. Sixty-seven per cent of patients had a high-grade MFH, with 41% involving the groin or popliteal fossa.
*Pathology.* Detailed analysis of surgical specimens was performed to determine per cent (%) tumor necrosis and assess surgical margins. If there was greater than 90% necrosis with negative margins, post-operative RT was not utilized. RT was given only if necrosis was less than 90% or for a positive margin. Good and poor responders were defined as those with greater and less than 90% tumor necrosis, respectively.
*Analysis.* Relapse- and disease-free survival was determined using the Kaplan–Meier method. Local recurrence was evaluated. Odds ratios were calculated based upon surgical margins and percentage of tumor necrosis for local recurrence as well as survival.
*Results.* Median follow-up was 51.5 months (range 19–133.4 months). The limb salvage rate was 92% (22/24 patients), overall survival was 87.5% and disease-free survival was 70.4%. Local control was 92% (22/24 patients). Both patients with local recurrence were reresected and remain alive and free of disease 29 and 85 months post-recurrence. Of the five patients who received RT, one died of metastatic disease, one is alive with metastases and the other three are disease free. Overall, five patients developed matastatic disease, of whom two remain alive. Median histologic tumor necrosis was 95% (range 5–100%). Close surgical margins were strongly associated with recurrent disease (one local and two metastatic) with an odds ratio of 10.5.
*Conclusions.* This study demonstrates that neo-adjuvant chemotherapy successfully shrinks most large and/or unresectable extremity STS allowing for limb-sparing procedures with excellent local control. The median percentage of tumor necrosis (95%) is comparable to that seen in neo-adjuvant treatment of osteosarcoma. This study suggests that RT is not necessary for all STSs, and can be limited to those patients with poor histological response or close surgical margins. We recommend induction chemotherapy for all difficult or unresectable high-grade STS of the extremities.

### Surgical resection and adjuvant intraperitoneal chemotherapy for recurrent abdominal sarcomas

F. C. Eilber, F. R. Eilber, G. Rosen & C. Forscher
(*UCLA Department of Surgery and Medical Oncology, Los Angeles, USA*)

Recurrent intra-abdominal sarcomas have proven to be resistant to systemic chemotherapy since many of these recurrences occur on the peritoneal and serosal surfaces. A pilot phase I–II study of resection of all gross disease, omentectomy and post-operative intraperitoneal chemotherapy (mitozantrone) was performed from November 1990 to January 1997. Fifty-three patients were treated. Histology: leiomyosarcoma (44) (stomach (6), small bowel (20), colon (2), mesentery (3), uterus (13)), hemangiopericytoma (1), synovial (1), osteo (1), liposarcoma (3). Disease was confined to the peritoneal cavity in 44; liver metastases were also present in 9 patients. Following resection of peritoneal implants (1–300), patients were treated via peritoneal catheters with mitozantrone 20 mg $m^{-2}$ in 1000 ml NS for 1–5 cycles q 1 month. Twenty-one out of 53 (40%) developed further abdominal recurrence and 15/53 (28%) remained alive from 1 to 7 years (median 30 months) following treatment. Intraperitoneal mitozantrone chemotherapy appears to be more effective than systemic chemotherapy in reducing the intraperitoneal recurrence of abdominal sarcomas. Treatment-related complications were small 3/53 (6%), and all were related to the third or fourth IP cycle of chemotherapy when drug distribution was restricted by adhesions; however, 29/53 (55%) developed hepatic metastases. In order to address these problems, future trials of intra-operative intraperitoneal chemotherapy combined with post-operative systemic chemotherapy are proposed.

**Dose-intensive chemotherapy with adriamycin, ifosfamide and growth factors in chemotherapy naive patients with soft tissue sarcomas**

S. R. Patel, S. Vadhan-Raj, M. A. Burgess, N. E. Papadopoulos, C. Plager, J. Jenkins & R. S. Benjamin
(*University of Texas, MD Anderson Cancer Center, 1515 Holcombe Blvd, Houston, TX 77030, USA*)

Adriamycin (A) and ifosfamide (I), are the two most active agents in sarcomas and both have been shown to have a steep dose–response relationship. Their combination, with or without other agents, has been used at less than full doses of each of the two agents which may have compromised the activity of the regimen. In a group of 79 evaluable patients treated with AI in three sequential studies, 50 objective responses were noted for an objective response rate of 63% (95% CI 52–74%, complete response 9%). Response rate for primary soft-tissue sarcoma (all sites) was 76% (28/37 patients); for extremity primaries only it was 80% (20/25 patients, 3 CR). The response rate for metastatic disease patients was 52% (22/42 patients). Responses in individual histologies were as follows: 12/20 (60%) MFH, 13/14 (93%) synovial, 9/14 (64%) unclassified, 6/13 (46%) non-GI leiomyosarcomas and 10/18 (56%) miscellaneous histologies. The first 16 patients were treated with A at 75 $mg\,m^{-2}$ and I at 10 $g\,m^{-2}$ every 3 weeks. Since then, we have treated 63 patients with A at 90 $mg\,m^{-2}$ given as a 72-h infusion, days 1–3 along with I at 2.5 $g\,m^{-2}$ over 3 h qd × 4, days 1–4. G-CSF was used at 5 $\mu g\,kg^{-1}\,day^{-1}$ from day 5 until AGC ≥ 10 000 and the last 50 patients have received thrombopoietin in an on-going trial. The median age of this group is 46 years (range 16–65 years), and there were 44 males and 35 females. Of the 29 patients who did not achieve an objective response, 7 patients (9%) have had a minor response (MR), 16 patients (20%) had NC and only 6 patients (8%) progressed on therapy. Approximately 300 cycles have been administered. The median AGC nadir was 0 (0.0–2.64) cells/$\mu l$ on day 11 lasting a median of 4 days (range 1–12 days). In 68 patients 141 cycles were complicated with febrile neutropenia. The median platelet nadir was 37.5 (3–212) × $10^3$ cells/$\mu l$ on day 13. Approximately 50% of patients who did not receive thrombopoietin experienced grades 3–4 thrombocytopenia in the first two cycles and virtually all patients had it by cycle 3. Eleven patients/14 cycles were complicated by grade 3 nausea and vomiting, 19 patients/31 cycles were complicated with grade 3 stomatitis requiring a reduction in the duration of A infusion to 48 h and 19 patients/28 cycles had grade 3 fatigue. One patient died due to cardiac toxicity after five cycles. We feel that in appropriately selected patients (age < 65 years, XRT to < 20% marrow, PS 0–1, no prior chemotherapy) this high-dose therapy is feasible and continues to produce a high response rate.

**Ppar-gamma ligands as differentiation therapy for liposarcomas: preclinical modeling and early phase II clinical trial results**

G. D. Demetri, B. Spiegelman, C. D. Fletcher, L. A. Stefanowicz, E. Geiger & S. Singer
(*Dana-Farber Cancer Institute and Brigham and Women's Hospital, Harvard Medical School, Boston, MA 02115, USA*)

Adipocyte differentiation has been a useful model for exploring molecular mechanisms which control proliferation and differentiation. Peroxisome proliferator-activated receptor-gamma (PPAR-$\gamma$) is a novel nuclear receptor which has been identified as a critical regulator of adipogenesis. PPAR-$\gamma$ forms a heterodimer with the RXR nuclear receptor, linking this pathway to retinoid-associated pathways of differentiation as well. Ligands for PPAR-$\gamma$ have been identified primarily using insulin-sensitizing assays, since PPAR-$\gamma$ plays a role in insulin responsiveness. Thiazolidinediones, including the first-generation compound troglitazone (TGZ), have been shown to be effective agonist ligands for PPAR-$\gamma$. TGZ has received regulatory agency approval as an insulin-sensitizing agent for control of refractory type II diabetes, and the safety profile of this agent is well understood. Because PPAR-$\gamma$ signaling pathways can stimulate terminal differentiation of adipocytes, preclinical modeling experiments were designed to assess whether PPAR-$\gamma$ ligands could induce terminal differentiation of human malignant liposarcomas *ex vivo*. These results have been published, *Proc Natl Acad Sci*, showing that all human liposarcomas studied, regardless of histologic grade or subtype, expressed the PPAR-$\gamma$ receptor, and that PPAR-$\gamma$ ligands have the ability to induce terminal adipocytic differentiation. These results strongly supported the translation of this strategy into a clinical trial, and this will be presented in detail. Patients with malignant liposarcomas that are not curable by multi-disciplinary approaches are eligible to participate in this study. Five patients have been accrued as of August 1997. Serial imaging studies and, whenever possible, serial biopsy evaluations, are being performed to assess adipocytic differentiation status of the tumors. TGZ, orally administered, has been very well tolerated with no adverse events in this population. With this translational research effort between the clinic and the collaborating laboratory groups, we hope to gain insight into the therapeutic potential of this novel differentiation strategy in humans. Other on-going studies to evaluate the mechanisms of the PPAR-$\gamma$ pathway and possible synergistic approaches with other mechanisms will be discussed.

**[Reichardt *et al.*, see page 219]**
**[Azzarelli *et al.*, see page 200]**
**[Baker *et al.*, see page 201]**

**Neo-adjuvant chemotherapy combined with regional hyperthermia followed by surgery and radiation in primary and recurrent high-risk soft tissue sarcomas of adults: updated report**

R. D. Issels,[1,2] S. Abdel-Rahman,[2] C. Salat,[2] M. H. Falk,[2] O. Ochmann[2] & W. Wilmanns[1,2]
(*[1]GSF-Institute of Clinical Hematology and [2]Medizinische Klinik III, Klinikum Grosshadern Medical Center (KGMC), University of Munich, D-81377 Munich, Germany*)

*Purpose.* This phase II trial was designed to evaluate response rate, time to progression and survival for neo-adjuvant chemotherapy combined with regional hyperthermia (RHT) followed by surgical resection and radiation in patients with locally advanced primary or recurrent high-risk soft tissue sarcoma (HR-STS).

*Methods.* Fifty-nine (31 primary/28 recurrent) eligible patients with HR-STS (tumor grade II/III tumor size >8 cm and deep) were treated at KGMC. Preoperative chemotherapy included doxorubicin (50 mg $m^{-2}$) on day 1, etoposide (125 mg $m^{-2}$) on days 1 and 4 and ifosfamide (1250 mg $m^{-2}$) on days 1–4 (EIA). RHT was given (1 h at 42°C) on days 1 and 4. After receiving four EIA cycles plus RHT, patients underwent surgical resection which was followed in turn by additional combination chemotherapy and radiotherapy.

*Results.* Clinical response rate (42%) evaluable in 52 patients included: 1 complete response (CR), 8 partial response (PR) and 13 minor response (MR) regressions. Seventeen patients showed stable disease (SD) and 13 patients progressive disease PD. Forty-nine patients underwent surgery. Forty were resected without mutilation. In 6/49 resection specimens, no viable tumor was present (6 CR). During follow-up (median 38 months), local recurrence occurred in 13 patients (22%) with distant metastases in 15/59 (25%) patients. The 5-year overall survival and distant disease-free survival estimates are 48% and 37%, respectively. At present, 30 patients are alive (median survival 50 months).

*Conclusions.* Based on these encouraging results, a randomized multi-centric phase III study (EORTC 62961/ESHO RHT 95) is on-going (activated July 97) comparing neo-adjuvant EIA chemotherapy with or without RHT in High Risk STS in order to prove further the impact of hyperthermia upon local progression-free survival and long-term survival.

**[Comandone *et al.*, see page 205]**

### Stage T1 intermediate and high-grade soft tissue sarcoma treated with surgery and radiotherapy

A. Pollack, G. K. Zagars, R. S. Benjamin, R. E. Pollock & P. W. T. Pisters

*(Departments of Radiation and Surgical Oncology, University of Texas, MD Anderson Cancer Center, Houston, TX 77030, USA)*

*Purpose.* To assess the efficacy of surgery plus radio-therapy (RT), the effect of treatment sequence and the value of other clinical–pathologic determinants of outcome for stage T1, N0, M0 soft tissue sarcoma.

*Patients and methods.* The cohort described comprised 179 patients with ≤ 5 cm, grade 2–3 malignant fibrous histiocytoma ($n = 117$), synovial sarcoma ($n = 40$) and liposarcoma ($n = 22$) that were treated between 1967 and 1992 mainly with surgery plus RT. Of these, 26% were intermediate grade, 51% were male and 13% received adjuvant chemotherapy. The tumors were located in the lower extremity in 91 patients, upper extremity in 45, trunk in 26 and head and neck in 17; retroperitoneal sarcomas were excluded. The average age was 48 ± 1 years (± SEM). Median follow-up was 97 months. Patients were classified into three groups based on the sequencing of surgery and RT at MDACC: Pre-op, pre-operative RT followed by excision at MDACC; Post-op, excision at MDACC followed by post-operative RT; and RT-alone ($n = 70$), RT without further surgery at MDACC.

*Results.* The 5- and 10-year actuarial rates were 82% and 80% for local control, 84% and 81% for freedom from distant metastasis and 84% and 75% for overall survival. In univariate analyses the only correlates of local control were primary treatment (not recurrent, $p = 0.002$, log-rank) and negative final margin status ($p = 0.1$). Primary treatment was the only predictor of local control in multivariate analysis. Type of treatment given at MDACC was not significant for the entire cohort; yet, was significant when stratified by presenting tumor status. For patients presenting to MDACC with gross disease, 5-year local control was higher (100 versus 71%) using Pre-op ($n = 8$) as opposed to Post-op ($n = 30$). The small numbers limited the power of this comparison ($p = 0.11$). For patients presenting after excision (no gross residual disease), 5-year local control was better (96 versus 75%, $p = 0.013$) using Post-op ($n = 47$) as opposed to Pre-op ($n = 24$). Re-excision at MDACC also proved beneficial as local control was improved (96 versus 81%, $p = 0.012$) using Post-op ($n = 47$) as opposed to RT-alone ($n = 70$). These effects were independent of whether the disease was treated primarily.

*Conclusions.* Optimal local control is obtained when presenting tumor status at referral (measurable versus no measurable disease) is considered in planning the sequencing of surgery and RT.

### Combined surgery and radiation therapy for soft tissue sarcoma of the subcutaneous tissues

I. J. Spiro,[1] A. E. Rosenberg,[2] M. Gebhardt[3] & H. D. Suit[1]

*(Departments of [1]Oncology Radiation, [2]Pathology and [3]Orthopedic Surgery, Massachusetts General Hospital and Harvard Medical School, Boston, MA 02114, USA)*

We reviewed the records of 26 patients with soft tissue sarcoma of the subcutaneous tissues, excluding angiosarcoma, treated at our institution between 1989 and 1997 with combined surgery and radiation therapy. The histologic subtype of these tumors was as follows: MFH (23), synovial sarcoma (1) and liposarcoma (2). The majority of MFHs were widely infiltrating (80%) and the remainder were well circumscribed (20%). Eleven of 26-patients had positive surgical margins. Tumor sites were as follows: thigh (10), leg (5), axilla (3), forearm (3), arm (1), knee (1), ankle (1), buttock (1) and flank (1). Actuarial local control and disease-free survival was 88% and 50%, respectively. There were two local failures, each salvaged with multiple operations and ultimately an amputation. There were one regional failure, three distant failures and two deaths. Despite the conventional wisdom that these tumors present less difficulty in management than deep intra-muscular tumors, infiltrating subcutaneous tumors can present a challenge to the surgeon. Patients with subcutaneous tumors have a high frequency of positive surgical margins and often require multiple surgical procedures with split thickness skin grafting. The optimal management of these patients and the indications for treatment by surgery alone or radiation therapy and surgery will be discussed.

### Poster presentations

### Molecular mechanisms of bone specificity of metastasis

S. Zawaideh & S. Ashkar
(*Laboratory for the Study of Skeletal Disorders and Rehabilitation, Department of Orthopaedic Surgery, Harvard Medical School and The Children's Hospital, Boston, MA 02115, USA*)

The ability of disseminating cancer cells to establish metastases in secondary organs is determined by a combination of factors including mechanical dislodgement of tumor cells, hemodynamic flow and specific host–tumor cell interactions. The distal metastasis of certain neoplasms, such as breast and prostate tumors to bone, cannot be explained by mechanical or hemodynamic factors alone and any model for bone-specific metastasis must account for the selective homing of tumor cells into bone. Bone is an organ that is continuously being remodeled, and factors released during the remodeling process stimulate the migration and subsequent growth of tumor cells in the bone environment. However, the identity of these chemotactic factors is unknown. Osteopontin, a major non-collagenous component of bone matrix, has been demonstrated to modulate the motility of several cell types including tumor cells. Osteopontin can interact with several cell-surface receptors including CD44 and integrins. CD44 is a glycoprotein that is involved in the migration of lymphocytes and tumor cells. CD44 is also highly expressed on metastatic breast carcinoma cells and its expression has been directly correlated to the metastatic potential of these cells. We demonstrate that the specific interaction of osteopontin with its target cells induces a host of physiological responses including chemotaxis, attachment and cell spreading, induction of metalloproteases 2 and 9, inhibition of apoptosis and induction of several cytokines including TGF$\beta$ and TNF$\alpha$. Moreover, we also demonstrate that antibodies raised against the chemotactic domain neutralize the migration of tumor cells to osteopontin. Based on these results we hypothesize that the interaction between osteopontin, or chemotactic peptides released from osteopontin during bone remodeling, and specific splice variants of CD44 on breast cancer cells is responsible for this organ specificity of metastasis. This unique interaction between osteopontin and tumor cells further defines the complex molecular events leading to bone-specific metastasis.

### Effective control of advanced aggressive fibromatosis with chemotherapy. Eight years of experience with methotrexate + vinblastine in 27 patients

A. Azzarelli, P. Casali, S. Fissi, A. Gronchi, A. Rasponi & R. Bertulli
(*Istituto Nazionale Tumori, Via Venezian 1, I-20133 Milano, Italy*)

*Purpose.* Aggressive fibromatosis (AF) can be an impossible challenge for surgeons, and incomplete operations seem to increase the local spread of disease until it is inoperable. With the purpose of improving the surgical results, or in place of surgery, since 1989 at the Istituto Nazionale Tumori, Milan, patients with advanced AF have been treated with chemotherapy.
*Materials and methods.* The regimen was methotrexate 30 mg m$^{-2}$, + vinblastine 6 mg m$^{-2}$ repeated every week for 1 year (52 doses) (Weiss regimen modified, *Cancer*, 1989). In the period 1989–1996, 27 patients received the treatment. All the patients had advanced lesions with evidence of tumor growth that either demanded amputation or were inoperable. The male/female ratio was 17/10 and the mean age was 33 years. By site: three abdominal and mesenteric (in Gardner's syndrome), eight of the scapular girdle, four of the pelvis or pelvic girdle, three of the thigh, six of the trunk and one of the arm.
*Results.* When evaluating the results, we were not able to employ the usual parameters of response; in fact, only four patients had an objective response in terms of shrinkage ≥ 50%. The first signs of response were: (a) improvement of symptoms (less pain if present, improved functionality if impaired), (b) reduction in size and mobility of the lesion or at least the cessation of growth, (c) a lower uptake of paramagnetic contrast at NMR. All these signs were regarded as indicators of response independent of the evidence of tumor shrinkage. One patient died from unrelated heart failure after two cycles. In the three patients with Gardner's syndrome, abdominal complications and emergency conditions made it impossible to continue the treatment; nevertheless, no progression of disease was seen during the few cycles of CT administered. No patient completed the treatment as scheduled, and only one received the 52 cycles but in 547 days (instead of 365). The mean number of delivered cycles/patient was 27 in 420 days, with a mean interval between cycles of 15 days. The main causes of delayed CT were: (a) myelotoxicity (after the first 12 cycles it was difficult to achieve complete recovery from myelo-depression exactly on the eighth day), and (b) hepatic toxicity was seen in six cases, always after the 20th cycle. The liver impairment normalized spontaneously on suspension of CT, and did not reappear after subsequent cycles. Most patients interrupted CT before the number of scheduled cycles for psychological reasons. Any kind of response, except pain, was not evaluable before 8–12 cycles of CT. After a median follow-up of 67 months (range 6–96 months) and a median interval after the end of treatment of 40 months (range 0–78 months) all 23 evaluable patients have stable or regressed disease, with fewer symptoms. Only one patient was re-operated on with a minor partial operation on the edge of the scapula.
*Conclusions.* AF is not necessarily a surgical disease and can be controlled by effective medical treatment with stable results. For advanced lesions the present results are superior to any kind of radiosurgical treatment (historical control). The comparison with tamoxifen (TAM) is a matter for investigation, but the following should be considered (a) TAM delivered for long period is not easily tolerated in young males, and has severe gynecological complications in females; (b) in our historical experience the response rate was not so frequent or stable. However, our future policy will integrate CT and TAM in a sequential fashion according to the patient's sex, age and expectancies of response.

### Communication with cancer patients in an orthopaedic ward

J. Baek-Jensen,[1] J. Keller[1] & J. Mainz[2]
([1]*Centre for Musculo-Skeletal Tumours, University Hospital of Aarhus &* [2]*Department of General Practice, University of Aarhus, DK-8000 Aarhus C, Denmark*)

*Aim.* The aim of the study was to examine the level of information and satisfaction among cancer and shoulder–elbow patients in an orthopaedic ward, to compare the level of information and satisfaction between patients with malignant and benign diseases, and the development of satisfaction over time.
*Patients and methods.* From November 1996 until May 1997, 38 consecutive patients (18 females, 20 males) under suspicion of cancer and 38 consecutive patients (23

females, 15 males) with a shoulder–elbow disease were included in the study. They were matched according to age. The patients in both groups were admitted to the-same ward and surrounded by the same staff. Three questionnaires were constructed containing closed and open questions about purpose and results of the tests, diagnosis and satisfaction with the information given. Patients were interviewed the day after admittance to the ward, again after having received information about treatment, and finally 1 month after discharge.
*Results.* On admittance to the ward the patients had a low level of information and knowledge regarding examinations and diagnosis and a low level of satisfaction. Following the first information, each patient's level of information and knowledge regarding examinations, diagnosis and treatment in both groups improved and remained high 1 month after discharge. Patient satisfaction also improved and remained high in both groups. There were no significant differences observed between the two groups with regard to the level of information and satisfaction. Nevertheless, 9/38 patients under suspicion of cancer and 12/38 shoulder–elbow patients had a wish for more information on prognosis and rehabilitation.
*Conclusions.* The level of information and satisfaction were high, and not influenced by the diagnosis of cancer. Despite these findings the results indicate that patients wish for more information regarding prognosis and rehabilitation.

### Malignant café au lait spots and sarcoma

L. H. Baker, M. C. Woo & E. Fearon
(*Divisions of Hematology/Oncology and Molecular Medicine and Genetics, University of Michigan Medical Center, Ann Arbor, MI 48109-0948, USA*)

Neurofibromatosis type I (NF1) is a common, autosomal dominant disorder. In addition to other features, more than 99% of post-pubertal affected individuals with NF1 have six or more café au lait macules over 15 mm in diameter. The most frequent malignant tumors seen in those with NF1 are gliomas and sarcomas, particularly neurofibrosarcomas, which are seen in about 1–3% of affected individuals. Sarcomas are, in general, a very rare cancer in adults, accounting for less than 1% of adult malignancies. We surveyed a consecutive series of breast cancer patients in our clinic, examining their skin completely for café au lait spots. Of 60 patients with breast cancer examined, no patient was found to have skin lesions consistent with the diagnostic criteria for NF1. However, of 40 patients with sarcoma, 12 had skin lesions that met the diagnostic criteria for NF1. None of the 40 patients had other diagnostic features of NF1, affected relatives with the diagnosis of NF1 or neurofibrosarcomas. The sarcomas seen in the 12 individuals, included four Ewing's sarcoma, four fibrosarcoma, two synovial sarcoma, and one case each of rhabdomyosarcoma and osteogenic sarcoma. The NF1 gene is a tumor suppressor gene spanning over 350 kb of DNA at chromosome 17q11.2. Although previous studies have suggested that the NF1 germ-line mutations are highly penetrant and no evidence for genetic heterogeneity has been reported, variable disease features and severity have been reported in some NF1 families. We are presently pursuing the hypothesis that a subset of the 12 sarcoma patients with skin lesions meeting the diagnostic criteria for NF1, but without other features of the disease, may harbor germ-line mutations in the NF1 gene. The identification of germ-line NF1 mutations in a significant fraction of sarcoma patients would have far-ranging implications.

### Differential expression of SAS, MDM2 and CDK4 in osteosarcoma: regulation of the CDK4 inhibitor p16INK4

M. S. Benassi, G. Gamberi, L. Molendini, P. Ragazzini, M. R. Sollazzo, C. Ferrari, G. Magagnoli, M. Merli & P. Picci
(*Laboratory of Oncologic Research, Istituti Ortopedici Rizzoli, I-40136 Bologna, Italy*)

*Introduction.* DNA amplifications or gains of SAS, MDM2 and CDK4 have been found in human sarcomas, suggesting their involvement in malignant transformation. Recently, a specific inhibitor of the CDK4-kinase activity has been identified as p16INK4 protein. The aim of this study was to evaluate the amplification of the SAS gene and the expression of MDM2, CDK4 and p16 protein in osteosarcoma specimens (OS) with different grades of malignancy.
*Methods.* SAS amplification was analyzed on genomic DNA extracted from paraffin-embedded tumor samples by the quantitative polymerase chain reaction method. The intensity of the DNA bands was determined by densitometric analysis. The protein expression and distribution were evaluated by immunohistochemistry using specific antibodies against the CDK4, MDM2 and CDKN2 (p16) products.
*Results.* Twelve per cent of the low-grade OS showed both SAS amplification and MDM2 over-expression. A strong and uniform nuclear expression of CDK4 and MDM2 proteins was also seen in 44 and 31% of the cases respectively. Concerning p16 protein, most of the tumor cells showed an intense nuclear immunoreactivity in 50% of the low-grade OS and in 39% of the high-grade OS.
*Conclusions.* These results confirm the role of SAS, MDM2 and CDK4 in OS tumorigenesis. The p16INK4 expression varied in relation to cell proliferation and histological grade emphasizing its influence on cell-cycle progression.

### Vascular margins in sarcoma surgery

J. Benevenia, M. Silva, Jr & D. Patel
(*New Jersey Medical School, Newark, NJ 07103, USA*)

Sarcomas commonly present about major vascular structures. The limited anatomic space in these areas poses problems of soft tissue coverage and neurovascular compromise. Vascular involvement may be the most critical determinant of limb salvageability. Vessel resection necessitates amputation or more complex vascular grafting. The purpose of this retrospective study was to compare the local recurrence rate of marginal vascular margin (adventitial dissection) with wide vascular margin (dissection through tissue/fat plane between tumor and vessels). Categories of vascular involvement were grouped into three possible stages: (1) no involvement of vascular tree with clear tissue plane between tumor capsule and vessels; (2) partial involvement of vascular tree with obliteration of tissue/fat plane between tumor capsule and vessels; and (3) complete encasement of major vessels by tumor. Marginal or wide margins were unable to be obtained in group 3 patients without vessel (and nerve) resection. Patients in group 2 had limb salvage with adventitial dissection and preservation of major vessels (marginal margin). Group 1 patients had limb salvage not necessitating adventitial vascular dissection (wide margin). A total of 104 patients were studied. There were 46 patients in group 1 (17 low-grade and 29 high-grade sarcomas) and 35 in group 2

(7 low-grade and 28 high-grade sarcomas) who underwent limb-salvage surgery. Twenty-three patients (2 low-grade and 21 high-grade) had amputations. The local recurrence rates for groups 1, 2 and 3 were 4.3%, 2.9% and 4.3%, respectively. All local recurrences were in high-grade lesions and occurred within 18 months. Overall local recurrence rate for limb salvage was 3.7%. Local recurrence rates for marginal vascular margins are comparable to those of wide resection and amputation. Patients with partial vascular involvement may not necessitate vascular resection-grafting for successful local control in extremity sarcomas.

**Preliminary evaluation of complete resection of major sarcomatosis followed by randomization of early post-operative intraperitoneal chemotherapy**

S. Bonvalot, A. Lecesne, P. Terrier, C. Le Pechoux, D. Elias & Ph. Lasser
(*Institut Gustave-Roussy, 39 rue Camille Desmoulins, F-94805 Villejuif Cedex, France*)

The purpose of this study was to estimate the ability to realize complete resection of major sarcomatosis and to appreciate the addition of early post-operative intraperitoneal chemotherapy (EPIC). From July 1996 to March 1997, 11 major sarcomatoses were operated on. On average, it was the second recurrence (first to fourth). Histology was four liposarcoma, three fibrosarcoma, five leiomyosarcoma and one synovialosarcoma. The median size of the main tumor was 20 cm (from 5 to 45 cm) and the median weight was 5 kg (from < 1 to 35 kg). Patients with complete resection were randomized to EPIC or not. When EPIC was decided, the abdomen was filled with 1 l $m^{-2}$ Ringer's solution, containing adriamycin 5 mg $m^{-2}$ and cis-platin 15 mg $m^{-2}$, at the end of surgery. This procedure was repeated every 4 days. Two patients had surgical exploration only, the limit was twice circular invasion of the superior mesenteric artery. Among the nine patients with complete resection, five received EPIC. Median time of surgery was 6 h (from 2 to 17 h). Once, surgery was realized with two procedures with a 1-week interval. Median blood loss was 1.4 l (< 1 to 5 l). On average, three organs were resected per intervention. One patient had definitive digestive stomia. There was no mortality. There was one duodenum fistula (patient who received EPIC). The mean hospital stay was 2 weeks without EPIC and 3 weeks with EPIC. To date, two patients have had a local recurrence, both received EPIC. Maximalist and complete resection of major sarcomatosis is technically feasible without excessive morbidity or functional prejudice. It allows survey without recurrence beyond 1 year. However, the adjuvant use of EPIC (adriamycin and cis-platin) increases operative follow-up and remains to be evaluated by further study.

**Treatment of adult Ewing's sarcoma, peripheral, neuroectodermal tumor and rhabdomyosarcoma with aggressive combination chemotherapy**

B. E. Brockstein, B. L. Samuels, K. Melton, M. Simon, T. Peabody & A. J. Mundt
(*University of Chicago, Chicago, IL 60637, USA*)

Adult Ewing's sarcoma, peripheral neuroectodermal tumor (PNET), and alveolar and embryonal rhabdomyosarcoma are rare with approximately 250 cases per year occurring in the US. As a result, no chemotherapy regimen is considered as standard for the treatment of these malignancies. Between June 1992 and July 1997, we treated 14 patients with a multi-drug regimen based on the control arm of the current Intergroup protocol for Ewing's sarcoma. Diagnoses included Ewing's sarcoma in six patients (extra skeletal three, tibia one, femur one, pelvis 1), PNET in five patients (chest wall two, pelvis one, gluteus one, kidney one) and rhabdomyosarcoma in three patients (metastatic in two). The median age was 30 years (range 22–70 years), eight female, six male. Chemotherapy (CHTX) consisted of alternating cycles every 3 weeks of the following: (1) CAV = cyclophosphamide (C), 1200 mg $m^{-2}$ over 1 h; doxorubicin (A), 75 mg $m^{-2}$ over 24 h, and vincristine (V), 2 mg iv push; (2) IE = ifosfamide (I), 1800 mg $m^{-2}$ over 1 h $\times$ 5 days with mesna uroprotection; and etoposide (E), 100 mg $m^{-2}$ over 1 h $\times$ 5 days. Of patients receiving radiotherapy (XRT) (seven) received two courses of IE during XRT. Non-biopsy surgery was performed in nine patients (prior to CHTX in five, during CHTX in four). G-CSF was given for 10 days, beginning the day after each chemotherapy cycle. A median of four cycles of IE (2–7) and three of CAV (1–8) were given. CHTX was stopped prior to the 'goal' of 17 cycles for the following reasons: prolonged cytopenia, 4; progressive disease, 4; fatigue, 2; patient choice, 2; high-dose chemo with stem cell reinfusion, 1; psychiatric illness, 1. Toxicities included the following for IE/CAV: grade 4 neutropenia in 71/64% patients grade 4 thrombocytopenia in 28/21% patients, and grade 3 thrombocytopenia in an additional 28/14% patients. Two patients received five cycles of combined V, A, E and I or C and experienced five episodes of grade 4 neutropenia and five admissions for neutropenic fever. Non-hematological toxicities were rare. Responses in eight patients with measurable disease included four complete responses, two with a minor response and two with progression. With a median follow-up of 32 months (13 patients), 5 patients (38%) have died and 8 patients (62%) are alive, seven free of disease. Considering non-pelvic, non-metastatic patients, 6/7 (86%) are alive and free of disease. In conclusion, this regimen is well tolerated, appears efficacious and should be further explored on a larger scale in these diseases.

**Dose-ranging pilot study of adjuvant ifosfamide followed by doxorubicin in resected soft tissue sarcomas**

G. T. Budd, R. Pelley, K. Marks, R. Crownover, M. Joyce, G. Muschler, R. Zehr, T. Bauer, D. Peereboom, L. Wood & R. M. Bukowski
(*The Cleveland Clinic Foundation, Cleveland, OH 44195, USA*)

It has been hypothesized that the sequential administration of active single agents at their maximum tolerated doses (MTD) is therapeutically superior to conventional combination chemotherapy in the eradication of micrometastases. In order to develop a regimen which would allow this hypothesis to be tested in soft tissue sarcomas (STS), we performed a dose-ranging adjuvant chemotherapy trial of single agent ifosfamide (IFX) for three courses followed by doxorubicin (DOX) for three courses. This trial was open to patients with high-grade localized STS who had undergone adequate local therapy and to patients who had undergone complete resection of recurrent or metastatic disease. Courses were administered every 3 weeks and were given with sargramostim (GM-CSF) 5 $\mu$g kg$^{-1}$ subcutaneously. Cohorts of 3–6 patients were entered into successively higher dose levels. Dose-limiting toxicity (DLT) is defined as one or more cycles with granulocytopenia $< 0.5 \times 10^9$/l for 5 days or longer or other toxicity of grade 3 or higher. Eleven patients have been treated to date, seven with primary and four with recurrent tumors. IFX 7.5 g m$^{-2}$ (given in divided doses over 1 h daily × 3) and DOX 75 mg m$^{-2}$ produced acceptable toxicity with 0/4 and 2/6 patients developing DLT, respectively. IFX 9.0 g m$^{-2}$ and DOX 90 mg m$^{-2}$ were unacceptably toxic, producing DLT in 3/5 and 3/3 patients, respectively. We are currently testing interpolated dose levels of IFX 8.25 g m$^{-2}$ and DOX 82.5 mg m$^{-2}$ (two patients entered to date). At a median follow-up of 25 + months (range 2 + –38 + months), a single patient has relapsed. As defined above, the MTD of IFX is $\geq$ 7.5 g m$^{-2}$ but $<$ 9.0 g m$^{-2}$ (total dose given over 3 days) and that of DOX is $\geq$ 75 mg m$^{-2}$ but $<$ 90 mg m$^{-2}$, when given with GM-CSF support. Further dose escalation of this regimen might be possible with an alternative definition of DLT, as only two patients developed febrile neutropenia. The relapse rate is lower than anticipated, which might be due to patient selection, short follow-up, sequential IFX and DOX, or the use of GM-CSF. Randomized comparisons of this regimen with: (a) no therapy, (b) GM-CSF alone or (3) concurrent IFX and DOX would be of interest.

**Phase II trial of first-line high-dose ifosfamide in advanced soft tissue sarcomas: final results a study of the Grupo Español de investigación en sarcomas**

J. M. Buesa, A. López-Pousa, O. Gallego, J. Martín, J. García del Muro, J. Bellmunt, P. Escudero & A. Poveda
*(Oncología Médica, Hospital General de Asturias, E-33006 Oviedo, Spain)*

*Introduction.* In this study were included adult patients with locally advanced or metastatic soft tissue sarcoma (STS), previously untreated with chemotherapy, measurable disease, PS $\leq$ 2 and creatinine clearance $>$ 60 ml min$^{-1}$.

*Treatment.* Ifosfamide (IFOS) 14 g m$^{-2}$ + mesna 7 g m$^{-2}$ by continuous infusion over 6 days every 4 weeks; GM-CSF sc, 5 $\mu$g kg$^{-1}$ × 10 days was added to those cycles with neutropenic fever or no hematologic recovery by day 28, and to all subsequent cycles; no dose reduction was applied. Forty-eight patients entered the study, 46 were fully evaluable (1 ineligible, 1 no data): age 49 years (range 21–68 years); male/female 23/23; PS 0 21, 1 16, 2 9; histology: leiomyos 17, MFH 8, lipos 5, neurog 5, synovial 4, other 7; number of measurable lesions: primary tumor 29, lung 15, liver 7, lymph node 6, intra-abdominal 6, cutaneous 2; number of cycles 163, median 2 (range 1–7); median dose per cycle 14 g m$^{-2}$ (range 8.23–14.87 g m$^{-2}$); 45 patients received $>$ 90% of the projected dose.

*Hematologic toxicity.* (CTC) (G2/G3/G4, per cent of 152 evaluable cycles): hemoglobin 41/38/4, leucocytes 4/16/80, granulocytes 7/11/79, platelets 2/11/18. In 90% of the cycles, leucocytes $\geq$ 3000/mm$^3$, granulocytes $\geq$ 1000/mm$^3$ and platelets $\geq$ 100 000/mm$^3$ occurred by day 27.

*Non-hematologic toxicity.* (Per cent of patients, G2/G3/G4): vomiting 63/8/2, infection 17/17/2, asthenia 41/22/0, neurocortical 11/28/0 (G3 = somnolence in 1 patient and transient hallucinations in 12); renal toxicity (per cent of patients): hematuria G2 2, G4 2, creatinine G1 11, turia G2 2, G4 2, creatinine G1 11, proteinuria G1 68, G2 8, glycosuria $>$ 0.3 g/24 h 11 patients.

*Supportive measures.* GM-CSF was given in 67 cycles (41%) due to neutropenic fever (36%), delay in hematologic recovery (6%), prior toxicity (55%) or protocol violation (3%); blood transfusions were required in 20% of cycles, and platelets in 9 patients; 21 patients were admitted because of neutropenic fever and five for other reasons (overall 20% of cycles).

*Objective activity.* 4 complete response (CR), 13 partial response (PR), 17 stable disease (SD), 9 progressive disease (PD), 2 tumor-related early deaths, 1 toxic death; response rate 17/46 (37%, 95% CI 23–51%). Responses were observed in leiomyos (1 CR, 2 PR), MFH (1 CR, 2 PR), lipos (1 PR), neurogenic (1 CR, 3 PR), synovial (1 CR, 1 PR), angios (1 PR), mixed mesod (1 PR) and unspecified uterine sarcoma (1 PR). The results of this study confirm the activity of high-dose IFOS in STS. This schedule causes an acceptable renal toxicity that is reversible and not cumulative, while S central nervous system side-effects are mild. The use of GM-CSF is advisable with these IFOS doses. Comparative studies should help to define the role of high-dose IFOS as a first-line therapy for the treatment of this group of tumors.

**Activity of doxorubicin after high-dose ifosfamide in soft tissue sarcomas of the adult. A phase II trial of the Grupo Español de Investigación en sarcomas**

J. M. Buesa, A. López-Pousa, O. Gallego, P. Escudero, J. Martín & A. Poveda
*(Oncología Médica, Hospital General de Asturias, Oviedo, Spain)*

Patients with measurable and locally advanced or metastatic soft tissue sarcoma (STS) progressing to first-line therapy with ifosfamide 14 g m$^{-2}$ were included in this study. Doxorubicin was administered by iv bolus at an initial dose of 75 mg m$^{-2}$ every 3 weeks. The dose was to be successively reduced to 60 mg m$^{-2}$ and 50 mg m$^{-2}$ in the presence of neutropenic fever, hematologic toxicity grade 3–4 or estomatitis $\geq$ G2, and treatment was delayed by 1 week if granulocytes were less than 1500/mm$^3$ and/or platelets less than 100 000/mm$^3$ by day 21. Those cycles with weekly counts were evaluated for hematologic toxicity. Eighteen patients were included in the study (1 not evaluable LTFU after 1 cycle, 17 evaluable for non-hematologic and 12 for hematologic toxicity). M/F 9/8; age 51 years (range 28–66 years); PS 0 12, 1 4, 2 1; prior radiotherapy 10 (4 in hematopoietic areas); measurable lesion: primary tumor 7, lung 7, lymph node 1, soft- tissue 1, intra-abdominal 1; histology: MFH 4, lipos 4, leiomyos 3, mixed mesod 2, angios 1, synovial 1, epithelioid 1, undiffer 1; total number of cycles 86, median 5 (1–11). Five patients started therapy at 60 mg m$^{-2}$ due to the severe hematologic toxicity suffered with high-dose ifosfamide, and 4/5 had a first nadir with less than 400 granulocytes/mm$^3$ (1 unknown). Dose intensity for all patients during the whole treatment period (mg/m$^2$/week, per cent of cycles): 20–27, 58% ($\geq$ 25, 25%); 15–$<$ 20, 32%; $<$ 15, 9%. The dose was reduced in 14 cycles and delayed in 27 due to hematologic toxicity (25), non-hematologic toxicity (2), both (6) or other reasons (8). Hematologic toxicity (CTC) (number out of 50 evaluable cycles, G3/G4): leucocytes 31/5, granulocytes 16/30, hemoglobine 3/1, platelets 1/1. Non-hematologic toxicity (number out of 85 cycles): vomiting G2 9, G4 2, stomatitis G2 5, G3 1, cardiotoxicity G2 2, infection G2 3, G3 1. Supportive measures (number of cycles): antibiotics 7, prophylactic CSF 8, admissions because of toxicity 5 (3 neutropenic fevers). Objective activity: 1 complete response (CR), 4 partial response (PR), 8 NC, 4 progressive disease (PD), remission rate 5/17 (29.4%, 95% CI 11–48%). Responses were observed in MFH (1PR), angios (1 PR) lipos (1 histologic CR, 1 PR), and mixed mesod (1 PR). Response to doxorubicin/prior response to ifosfamide: 1 CR/1 NC, 4 PR/3 PR + 1 NC, 8 NC/3 PR + 3 NC + 2PD, 4 PD/1 RC + 1 NC + 2 PD. The results of this small study indicate that doxorubicin is an active drug in those patients failing to respond to high-dose ifosfamide, and that the spectrum of activity appears quite similar. Only 25% of the cycles could be delivered at the projected schedule of 75 mg $m^{-2}$ every 3 weeks, mostly due to the hematologic toxicity encountered. Prior therapy with ifosfamide might reduce the tolerance to doxorubicin.

### Clinicopathological evaluation of lung metastases in soft tissue sarcomas

A. Buonadonna,[1] G. Boetner,[2] B. Pasquotti,[2] A. D'Alessio,[1] P. Stefanovski,[1] G. Lo Re,[1] M. Duati,[3] C. Rossi,[2] V. Canzonieri,[4] A. Carbone[4] & S. Frustaci[4]

(*Divisions of [1]Medical Oncology, [2]Surgery, [3]Radiology & [4]Pathology, Centro di Riferimento Oncologico, I-33081 Aviano, Italy*)

In soft tissue sarcomas, lungs represent the first site of recurrence in over 50% of patients. Surgical resection is reported as curative in 20–30% of cases. This report is a prospective study on 36 patients, affected by metastatic soft tissue sarcomas, who underwent resection of lung metastases. The characteristics of the 31 patients evaluated so far are: median age 48 years (range 18–75 years), male/female 18/13, site of primary was limbs in 68% of the cases, and there was high-grade histology in 90%. After definitive local control of the primary, only 19% of the patients were submitted to adjuvant chemotherapy. Follow-up was performed with clinical evaluation and X-rays of the lungs every 2 months. At the onset of lung nodule(s), all patients but one were submitted to a computed tomography (CT) scan, and 61% received chemotherapy before thoracotomy. A comparison between X-rays, CT scan, surgical and pathological specimen is reported:

| Nodules | X-rays | *p* | CT scan | Surgery | Pathology |
|---|---|---|---|---|---|
| No. seen/ removed | 64/166 | ≤0.001 | 100/166 | 166/166 | 128/166 |
| Per cent | 38 | | 60 | 100 | 77 |
| Median | 2 | | 2 | 3 | 3 |
| Mean | 2.2 | ≤0.02 | 3.2 | 3 | 3 |
| Range | 0–11 | | 1–11 | 1–18 | 1–18 |

Bilaterality was detected in 33% of patients by X-rays and in 52% by CT scan. In 17 patients, a mono-bilateral thoracotomy was performed, whereas median sternotomy was performed in the remaining 14 patients. Macroscopical radicality by the surgical procedure was obtained in 84% of the patients. Statistically significant correlations were found between the first DFS versus 12 months (primary treatment–pulmonary relapse interval) for grading 2 versus 3–4 ($p = 0.04$) and for adjuvant chemotherapy versus control ($p = 0.04$). For the second DFS versus 12 months (radical pulmonary resection–pulmonary relapse interval) significant correlations were found for the sum of the maximum diameters of all removed nodules expressed as $< 20$ versus $\geq 20$ mm ($p = 0.01$). The CT scan was superior to standard X-rays but inferior to the pathological findings. Whether the sum of the maximum diameters of all positive nodules, expressed in millimeters, correlates with the second DSF should be further confirmed in a larger number of patients.

### Gastrointestinal stromal tumor, uncommitted type, with monosomy 14 and 22 as the only chromosomal abnormalities

L. Casorzo & M. Risio
(*Ospedale San Lazzaro, Alba, 12051 Italy*)

Gastrointestinal stromal tumors (GISTs) are non-epithelial neoplasms arising from cells located in the wall of the stomach and small bowel, and are characterized by remarkable variability in their differentiation potential. Most GISTs show immunohistochemical and ultrastructural evidence of differentiation towards smooth muscle cells or neural differentiation. GISTs with dual differentiation towards smooth muscle and neural elements, and tumors lacking differentiation towards either cell type ('uncommitted') are quite infrequent. Consistent chromosomal changes have been previously observed in malignant GISTs of smooth muscle type: multiple numerical changes, including monosomy of chromosome 14, are associated with structural rearrangements involving chromosome 1. Little is known about karyotypic profiles of GISTs with neural differentiation. Monosomy or deletion of chromosome 22, however, has been shown in a variety of nervous system tumors. Here we report the karyotypic analysis of a case of gastric stromal tumor with the histological and immunohistochemical features of a malignant, uncommitted lesion. Clonal monosomy of 14 and 22 were the only chromosomal alterations found. Genomic loss in these chromosomes is likely to be the early event in the malignant transformation of primitive mesenchymal cells of the gastrointestinal wall. Subsequent chromosome abnormalities could then drive the differentiation process towards distinct phenotypical features. If confirmed in further cytogenetic investigations, monosomy of chromosomes 14 and 22 could be used as a reliable marker of GISTs of uncommitted type.

### Characterization of newly established human chondrosarcoma cell line, CS-OKB

T. Chano, H. Okabe, Y. Saeki, M. Ishizawa, M. Matsumoto & S. Hukuda
(*Shiga University of Medical Science, Seta, Ohtsu, Shiga 520–21, Japan*)

A clonal cell line, CS-OKB, was derived from a human chondrosarcoma and characterized by cytogenetic study, immunocytochemical staining and reverse transcriptase–polymerase chain reaction (RT–PCR). Chromosomal abnormalities which were characteristic of malignant cartilaginous neoplasms were identified. Immunocytochemically, CS-OKB cells were intensely stained with anti-type II collagen and anti-keratin sulphate antibodies. Using RT–PCR, CS-OKB transcribes cartilage-specific genes such as type II, X procollagen and aggrecan. The human chondrosarcome cell line, CS-OKB, was confirmed as the stable cell line that synthesizes well-differentiated chondrocyte-specific molecules and expresses well-differentiated chondrocyte-specific genes in uncoated plastic dishes. CS-OKB could be connected into the human chondrocytic cell line and be useful and invaluable not only in studies on human chondrocytes, but also for studying the biological characteristics of human chondrosarcomas.

### Phase II trial of doxil in advanced soft tissue sarcomas

T. Chidiac, G. T. Budd, R. Pelley, K. Sandstrom, D. Mclain, R. Crownover, K. Marks, G. Muschler, R. Zehr, M. Joyce & R. Bukowski
*(Cleveland Clinic Cancer Center, Cleveland, OH 44195, USA)*

*Objective.* To assess the objective response rate and toxicity of doxil in previously untreated (soft tissue sarcoma) for advanced disease. Secondary endpoints are time to response, duration of response, progression-free survival (PFS) and overall survival (OS).
*Methods.* We treated patients in a two-stage accrual phase II design to allow the study to be terminated early should preliminary results indicate that doxil has little or no activity in this population. A total of 28 patients were planned to be entered if one or more responses were observed in the first 14 patients. The study opened on 10/22/96. Doxil was administered at 50 mg $m^{-2}$ every 4 weeks. We have since treated 12 patients of whom only 11 are evaluable. Male/female ratio was 8/4. Median age 61.5 years (range 34–75 years). ECOG performance status: 0 in 3/12, 1 in 7/12 and 2 in 2/12 patients. Leiomyosarcoma (6/12) and malignant fibrous histiocytoma (2/12) were the most common tumors.
*Results.* With a median number of 2 courses (range 1–4 courses) of chemotherapy, we obtained no objective responses in the 11 evaluable patients. One patient is still on the study. Grade 3 and 4 toxicity (leukopenia, neutropenia, anemia, confusion, deep vein thrombosis and mucositis) occurred in a total of four patients. Only one patient with grade 4 neutropenia had a dose reduction. Palmar-plantar erythema developed in 3/12 patients (grade 1 in 2/12 and grade 3 in 1/12). The median follow up of the study was 4.4 months (range 2.5–7.2 months five of the twelve) patients have died. Kaplan–Meier estimates of median PFS and OS are 1.9 and 6.8 months, respectively.
*Conclusions.* Doxil was well tolerated. However, no responses have been observed to date. Updated results will be presented.

### Drug resistance studies using fresh tumour cells from cases of soft tissue sarcoma: the roles of the multi-drug resistant phenotype and the tumour suppressor gene p53

H. M. Coley,[1] M. W. Verrill,[1,2] I. R. Judson[1,2] & C. Fisher[2]
*([1]CRC Centre for Cancer Therapeutics, Institute of Cancer Research, Sutton, Surrey & [2]Sarcoma Unit, Royal Marsden NHS Trust, London, UK)*

*In vitro* data generated from more than 60 fresh surgical samples of soft tissue sarcoma (STS) have been evaluated with respect to clinical outcome in order to clarify the process of clinical drug resistance. Techniques of chemosensitivity testing, immunocytochemistry, flow cytometric analysis (FACS) and gel electrophoresis using single-strand conformational polymorphism (SSCP) have been used to examine the roles of multiple drug resistance (MDR) and p53 in this disease. We have been able to identify a number of patients with tumours showing evidence of *in vitro* drug resistance to ifosfamide and/or doxorubicin, and this was reflected in the clinical outcome. For example, in a case of metastatic nerve sheath tumour, disease progression was seen following the administration of doxorubicin. There was evidence of the multiple drug resistance transporter protein P-glycoprotein and resistance to doxorubicin and ifosfamide was measured using *in vitro* analysis. In addition, this case showed a mutation of the p53 tumour suppressor gene protein (revealed by SSCP analysis). The patient succumbed to their disease 14 months after initial diagnosis. The combination of multiple drug resistance and p53 mutation was shown to be associated with a poor prognosis and was seen in a number of other cases. In serial samples from a patient with locally recurrent disease, we have demonstrated changes in chemosensitivity and drug resistance markers (P-glycoprotein and function). We intend to expand this aspect of the study using serial core biopsies in order to monitor the course of disease progression with regard to drug resistance markers.

### Efficacy and tolerability of an ifosfamide continuous infusion in soft tissue sarcoma patients

A. Comandone,[1] S. Frustaci,[2] A. Bearz[2] A. Boglione,[1] A. Buonadonna,[2] P. Stefanovski[2] & C. Bumma[1]

*(Divisions of Medical Oncology of [1]Torino and [2]Aviano, Italy, on behalf of the Italian Group on Rare Tumours)*

The treatment of relapsed soft tissue sarcoma (STS) patients represents a frustrating issue, since only three agents are active for this disease, namely anthracycline (ADM/EPI), ifosfamide (IFO) and dacarbazine. Other old and new agents have failed to be effective in second-line phase II trials. Recent data have indicated that IFO can be given as a continuous infusion (ci) for several days at a low daily dose, achieving high cumulative doses without acute toxicities. This modality of administration can therefore, also be employed in old or partially compromized previously untreated patients. The aim of the study was to verify the feasibility, toxicity and efficacy of an IFO-ci of 1 g $m^{-2}$ $day^{-1}$ prolonged for up to 21 days, when administered for 2–3 cycles. From March 1996 to June 1997, 36 patients were entered into this phase I–II trial. Their characteristics are: M/F ratio: 16/20, median age: 52.5 years (range 31–76 years), histology: LMS 12, pleomorphic sarcoma 5, synovial sarcoma 5, liposarcoma 5, IFM 4, m. schaw. 3, angiosarcoma 1, m. histiocytoma 1, respectively. Site of primary was: lower arm in 15 patients, upper arm, trunk, uterus in 5 patients each, breast and GI tract in 2 patients, buttock and larynx in 1 pt, respectively. Sites of metastases were: lungs in 28 cases, local relapse in 10, liver in 5, others (kidney, bone, pancreas) in 3, respectively. Pretreatment for the primary consisted of surgery alone in 15 patients, surgery + radiotherapy in 16, chemotherapy and/or radiotherapy in 5. Adjuvant chemotherapy was given in 5 patients, palliative chemotheraphy, for the same parameter lesions, consisted of anthracycline and ifosfamide in 29 patients, 7 patients were previously untreated with chemotherapy because of age ($>65$ years) or poor clinical conditions ($PS \geq 2$). Previous median total dose of IFO was 64.5 g $m^{-2}$ (range 10–96 g $m^{-2}$), whereas it was 443 mg $m^{-2}$ (range 61–937 mg $m^{-2}$) for ADM and/or 755 mg $m^{-2}$ (range: 216–1020) for EPI, respectively.

Median interval between progression of parameter lesions and start of IFO-ci was 2.7 weeks (range 1–21 weeks). Overall, the median number of administered cycles was 3 (range 1–6). The median duration of cycles was 15 (range 10–20), 14.5 (10–21) and 13.5 (9–20) days for the 1st, 2nd and 3rd administered cycles, corresponding to 26, 26, 25 $g\,m^{-2}$, respectively. Non-hematological toxicities were represented by G1–2 N/V in 25% of patients and G3 alopecia in 100% of patients. No hemorrhagic cystitis or microhematuria were observed. Myelosuppression was the main toxicity. G3/G4 leucopenia was observed in 7/1 patients, G3/G4 thrombocytopenia in 2/1 and G3/4 anemia in 1/0 patients, respectively. Overall, we observed 1 complete response (CR) and 7 partial response (PR) for a 24% response rate, and 8 NC and 17 progressive disease (PD); 3 patients are not yet evaluable. The median duration of response was 24 weeks (range: 8–28 weeks), whereas the overall time to progression was 13 weeks (range 0–28 weeks). Responses were observed in both untreated and pretreated patients. The overall response rate of this original schedule associated with the good profile of toxicity deserves further formal investigation inside a prospective phase II trial. The capability to induce responses in highly IFO-pretreated patients is the main finding of this study.

### A phase I/II study of a 72-H continuous infusion of etoposide in recurrent soft tissue sarcoma

C. R. Crawley, I. J. Judson, M. Verrill, C. Hill & F. I. Raynaud
(*Sarcoma Unit, Royal Marsden Hospital, Fulham Road, London, UK*)

*Purpose.* Etoposide has been used with ifosfamide as first-line treatment for advanced and metastatic soft tissue sarcoma (STS) in Scandinavia. However, its contribution to the activity of this combination is unclear. Therefore, we wished to assess the activity and toxicity of the same etoposide schedule, a 72 h continuous infusion, in patients with progressive STS.
*Patients and methods.* Open phase I/II trial performed at a single institution in patients with metastatic or locally advanced STS who had failed first-line treatment. Patients with alveolar or embryonal rhabdomyosarcoma and Ewing's/PNET were excluded. The starting dose level was 200 $mg\,m^{-2}\,day^{-1} \times 3$ escalating in 10% steps in cohorts of three patients until dose-limiting toxicity was encountered. Plasma steady-state etoposide levels were measured on day 2 and 3 of the first two treatment cycles.
*Results.* No responses were seen in 16 assessable patients despite cytotoxic etoposide levels. The median event-free survival was 6 weeks (95% CI 3.31–8.69) and the overall survival was 16 weeks (95% CI 9.28–22, 72). The maximum tolerated dose in this pretreated patient group was 200 $mg\,m^{-2}\,day^{-1} \times 3$. The dose-limiting toxicity was myelosuppression. Plasma etoposide levels were available for 12 patients. Mean etoposide levels were above previously reported cytotoxic thresholds (range 8.3–23.1 $\mu g\,ml^{-1}$). Etoposide levels did not correlate with toxicity or dose.
*Conclusions.* Etoposide given by 72 h infusion is inactive as second-line chemotherapy in STS. Etoposide is associated with significant toxicity when given in these doses, in this patient group.

### Introduction of a Web-based international musculoskeletal tumor registry

R. L. Crownover, A. Bhatia, P. W. T. Pisters, F. R. Eilber, P. Picci, I. Spiro & F. Coevorden
(*The Cleveland Clinic Foundation, Cleveland, OH 44195-5213, USA*)

A Web-based international musculoskeletal tumor registry has been developed for use by CTOS members. The registry is accessible from any browser-capable computer with access to the global internet. The early portion of this presentation will demonstrate software features by logging into the system and navigating among the available options. Additional discussion will include structure and cost of the registry, security/confidentiality issues, uses of gathered information and approaches to study design by participating CTOS members. It is anticipated that physicians will begin using the registry when they return home after the meeting.

### Near-haploidy may characterize inflammatory leiomyosarcoma as a distinct entity

P. Dal Cin,[1] R. Sciot,[2] C. D. M. Fletcher,[3] I. Samson,[4] R. De Vos,[2] N. Mandahl,[5] H. Willén,[5] O. Larson[6] & H. Van den Berghe[1]
(*[1]Center for Human Genetics, [2]Department of Pathology and [4]Department of Orthopedic Surgery, University of Leuven, B-3000, Belgium, [3]Department of Pathology, Brigham & Women's Hospital, Boston, USA, [5]Department of Clinical Genetics, University Hospital Lund & [6]Department of Tumor Biology, Karolinska Institute, Stockholm, Sweden*)

Inflammatory leiomyosarcoma is a recently described variant of leiomyosarcoma arising in deep soft tissue which should be differentiated from the inflammatory variant of malignant fibrous histiocytoma. We cytogenetically investigated an inflammatory leiomyosarcoma in the right thigh of a 28-year-old male and found a near-haploid karyotype: 28, X, +5, +18, +20, +21, +22. In a series of 78 consecutive MFHs analyzed by the Lund group Aspberg and coworkers noted that haploidization (chromosome number up to 34) was a relatively common phenomenon in this tumor type. We had the opportunity to review the histopathologic features of those MFHs reported in literature with a near-haploid karyotype by the Lund group. We found that only a lesion had the typical features of inflammatory leiomyosarcoma and, moreover, it showed an identical near-haploid karyotype. Although additional cases should be karyotyped, the identical near-haploid karyotype further supports that this variant of leiomyosarcoma is a separate entity or a special subset within this group of sarcomas.

### Distal upper extremity function following proximal humeral resection and reconstruction for tumors: contralateral comparison

T. A. Damron, M. G. Rock, M. I. O'Connor, M. E. Johnson, K. N. An, D. J. Pritchard, F. H. Sim & T. C. Shives
(*Mayo Clinic, Rochester, MN 55905 and SUNY Health Science Center, Syracuse, NY 13202, USA*)

*Background.* Most functional analyses following limb-salvage operations about the shoulder have focused on proximal function with the assumption that distal function is largely unaffected.
*Objective.* To examine prospectively collected laboratory data for upper extremity strength distal to the shoulder as well as the functional capability of the reconstructed upper extremity following oncologic limb-sparing procedures which include excision of the proximal humerus.
*Methods.* Objective laboratory data on 32 patients were collected over a 16-year period. Age averaged 35.3 years (range 11–74 years). There were five (15.6%) Tikhoff–Linberg resections, nine (28.1%) modified Tikhoff–Linberg resections, fourteen (43.8%) limited proximal humeral resections and four (12.5%) extended proximal humeral resections. Reconstruction consisted of osteo-articular proximal humeral allograft in nine, allograft–prosthetic composite in one, arthrodesis in seven and a custom prosthesis in fifteen cases.
*Results.* Follow-up duration averaged 42.3 months (range 7–117 months). Statistically significant reductions on the involved side compared to the uninvolved side in grip, forearm pronation, forearm supination, elbow flexion and elbow extension strength were documented ($p < 0.05$). The magnitude of reduction in strength diminishes distally, with the greatest effect in this group of patients being observed in elbow extension, followed by elbow flexion, forearm supination and forearm pronation. Grip strength consistently showed the least amount of strength reduction compared to the uninvolved side, even within resection and reconstruction groups. Subjective patient rating of dexterity was no less than 3/5. Ninety per cent of patients rated their dexterity 4/5 (52%) or 5/5 (38%).
*Conclusions.* Despite the insistence of normal function in the distal upper extremity following limb-salvage procedures, complete normality is not maintained. However, the degree of maintenance of distal function appears to be high and patient satisfaction is very acceptable.

### Sparing of radiation-induced damage to the physis by radioprotectant drugs: laboratory analysis in a rat model

T. A. Damron, R. M. Tamurian & J. A. Spadaro
(*Department of Orthopedic Surgery, SUNY Health Science Center at Syracuse, NY 13210, USA*)

*Background.* The radioprotectant free-radical scavenger drug amifostine (S-2 (3-aminopropylamino)-ethylphosphorothioic acid), administered just prior to radiotherapy, has been demonstrated to protect normal cells from ionizing radiation to a greater extent than tumor cells. Much is known regarding its effects on various soft tissues, bone marrow hematopoietic stem cells and splenic T-lymphocytes, but not to our knowledge on growing bone.
*Purpose.* The aim of this pilot study is to determine if amifostine can preserve the integrity of, or minimize the damage to, the physis during radiation exposure in an animal model.
*Methods.* Weanling Sprague–Dawley rats were randomized into five groups. Group 1 (lo-dose radiation alone): distal femur and proximal tibia in each animal received 1250 rads alone. Group 2 (hi-dose radiation alone): unilateral 1750 rads alone. Group 3 (lo-dose with amifostine): intraperitoneal amifostine (100 mg $kg^{-1}$) 20 min prior to 1250 rads. Group 4 (hi-dose with amifostine): amifostine 100 mg $kg^{-1}$ 20 min prior to 1750 rads. Group 5: control—neither radiation nor amifostine. At 6 weeks following radiation, the animals' limbs were harvested. Femoral and tibial lengths were measured in treated and untreated legs and compared to baseline length. Microscopic sections of the proximal tibial physeal region were prepared.
*Results.* Concordant with previous reports in the literature, the radiation dose of 1250 and 1750 rads reduced net growth in length by 22 and 63%, respectively, in the treated leg. Administered prior to this single-dose regimen of radiotherapy, intraperitoneal amifostine administration at 100 mg $kg^{-1}$ reduces this loss of growth to 13.6 and 46.2%, respectively ($p < 0.05$).
*Discussion.* Having established a beneficial effect of amifostine with a single radiation dose strategy, higher amifostine doses and more clinically relevant fractionated radiation strategies need to be examined in this animal model.

### Correlation of objective laboratory data and MSTS scores following shoulder joint tumor resection/reconstruction

T. A. Damron, M. G. Rock, M. I. O'Connor, M. E. Johnson, K. N. An, D. J. Pritchard, F. H. Sim & T. C. Shives
(*Mayo Clinic, Rochester, MN 55905 and SUNY Health Science Center, Syracuse, NY 13202 USA*)

*Introduction.* The validity of the Musculoskeletal Tumor Society (MSTS) functional evaluation system has not been established relative to objective laboratory analyses. Our purpose was to assess the validity of this tool by comparison with laboratory-generated objective strength and range of motion data.
*Methods.* Thirty-two patients with bone tumors of the proximal humerus or scapula treated surgically by resection of the shoulder joint including the proximal humerus from 1976 to 1992 underwent laboratory assessment of shoulder active range of motion and distal upper extremity isometric strength. Patients also completed MSTS functional assessment questionnaires. The MSTS scores analyzed because of a hypothesized relationship to the laboratory data included the cumulative, functional activity, hand positioning and lifting scores.
*Results.* The cumulative MSTS score showed a consistent trend toward a direct relationship with nearly all of the objective shoulder motion and distal upper extremity strength variables. The most significant associations with the subjective MSTS hand positioning score were direct relationships with active shoulder flexion range ($p = 0.0001$) and active shoulder abduction range ($p = 0.017$). There was no consistent linear relationship between either the MSTS lifting score or the MSTS functional activity score and the objective data. Comparison of the two types of shoulder range of motion assessments showed that the patients were most accurate in their estimation of shoulder abduction and flexion and poor in assessing their internal and external rotation.
*Conclusions.* This study suggests generally good correlation between the overall MSTS score and most objective measures of shoulder motion and distal upper extremity strength but generally poor correlation between individual MSTS scores and objective data.

| Age/ Sex | Site | Sxs Dur | Size of mass | Contr. Rec. | Duration of DM | Insulin (years) | Retinopathy | Nephropathy | Neuropathy | F/U | Bx | Compl. Bx |
|---|---|---|---|---|---|---|---|---|---|---|---|---|
| 28 F | Quad/biceps | 10 days (CT) | None | Prior hx & @ 6 weeks | 11 | 11 | + | + | + | Death 6 months × 2 | Inc | – |
| 31 F | VM/sartorius /gracilis | 3 weeks (MRI) | 10 cm | None | 0 | 0 | – | – | – | 15 months | None | N/A |
| 59 M | Med thigh | 3 weeks (MRI) | 5 × 8 cm | None | 12 | 10 | + | – | + | 20 months | Inc | – |
| 48 M | Med thigh | 4 weeks | 4 × 5 cm | None | 15 | 0 | + | + | + | 12 months | None | N/A |

### Diabetic skeletal muscle necrosis: a soft tissue sarcoma simulator

T. A. Damron, E. M. Levinsohn, T. M. McQuail, H. E. Cohen, M. Stadnick & M. Rooney
*(Departments of Orthopedics, Radiology and Pathology, SUNY Health Science Center, Syracuse, NY 13202, USA)*

*Background.* Necrosis of skeletal muscles in diabetic patients has been well described in the diabetic and radiologic literature, but is not widely recognized in the oncology community despite the fact that its presentation commonly mimics that of a soft tissue sarcoma.
*Objective.* To present the clinical and radiographic features of this process, and to review the appropriate diagnostic evaluation and treatment.
*Methods.* We report a series of four new cases and review all 25 cases previously reported in the literature.
*Results.* Demographic features of this disease process include a mean age of 37 years (range 25–64 years) and absence of gender bias. Mean duration of diabetes (DM) was 16.3 years (range 2–27 years). Either retinopathy, neuropathy or nephropathy was present in all but two. Symptoms of exquisite pain, tenderness and focal swelling in the lower extremity are present typically for less than 1 month prior to presentation. The swelling characteristically occurs in the large muscles of the thigh and results in the formation of a mass which mimics a tumor, abscess or hematoma. Magnetic resonance imaging (MRI) shows slightly increased intramuscular signal on proton density and T1-weighted sequences, markedly increased signal on T2-weighted sequences, a pattern indicative of intramuscular edema in the absence of a discrete muscle-displacing mass. Histologic findings consist of coagulative or hemorrhagic skeletal muscle necrosis and microangiopathy. The symptoms typically resolve without intervention in 3–4 months. Early physical therapy has been associated with prolongation of symptoms and therefore has generally been avoided. Our patients were similar to those reported in the literature in most respects. However, our series included the only patient of which we are aware in whom the muscle necrosis represented the presenting manifestation of diabetes, and one of only two patients with long-standing non-insulin-dependent diabetes mellitus.
*Conclusions.* Diabetic muscle necrosis should always be considered in the differential diagnosis of a lower extremity soft tissue swelling in the appropriate patient. We recommend biopsy in all cases not progressively resolving. However, the MRI characteristics, in the appropriate clinical setting, are highly suggestive, supporting close clinical observation when the patient is already improving at presentation. Radiographic and clinical features highly suggestive of the diagnosis include involvement of more than one muscle group, diffuse swelling within involved muscles rather than displacement of normal structures by a mass, short duration of symptoms, exquisite tenderness and history of long-standing diabetes. Only supportive treatment is advised.

### Radiation therapy and conservative surgery for soft tissue sarcomas of the extremities, superficial trunk and head and neck: an analysis of disease control, patient survival and prognostic factors

A. De Paoli,[1] G. Bertola,[1] F. Gherlinzoni,[2] G. Boz[1] A. Dedkov,[1] R. Talamini,[1] S. Frustaci,[1] M. G. Trovò,[1] C. Rossi[1] & A. Carbone[1]

*([1]CRO, I-33081 Aviano & [2]IOR Bologna, Italy)*

Long-term results were analyzed in a series of patients with primary (130) or recurrent (21) MO soft tissue sarcoma of the extremities (83%), superficial trunk (11%) and head and neck (6%) treated with radiation therapy (RT) and surgical resection (S) at the CRO between May 1985 and October 1996. Median age was 54 years (range 17–80 years) and male–female ratio was 1.1: 1. Thirty-four patients (23%) not amenable to conservative S underwent pre-operative RT with 44.8 Gy/28 fr (BID regimen) and 117 patients (77%) underwent local excision and post-operative RT with 64–66 Gy/32–33 fr. Pre-operative RT allowed conservative chemotherapy in all 34 patients with initially unresectable tumor; in no case was the tumor cut across. A post-operative boost of 16 Gy/8 fr was given to patients with positive or close margins of resection. A subset of 22 patients (15%) were treated with chemotherapy according to a randomized trial of adjuvant chemotherapy for high-risk patients carried out at our institute since 1992. Overall, surgical margins were negative in 62% and positive in 38% of patients. Tumor size ≥ 5 cm (range 5–28 cm) was reported in 103 patients (69%) and 48 (31%) had tumor size < 5 cm (range 2.5–4.5 cm). High-grade tumors (G3–4) were reported in 105 patients (70%) and G2 and G1 tumors in 25 (16%) and 21 (14%) patients, respectively. On 30, April 1997 the median follow-up was 60 months (range 6–143 months). Five-year actuarial results by treatment modality are reported in the table:

| Treatment | Number of patients | Local control | DFS | OS |
|---|---|---|---|---|
| S + post-operative RT | 117 | 88% | 68% | 76% |
| Pre-operative RT + S | 34 | 78% | 23% | 40% |
| Total | 151 | 86% | 60% | 70% |

Tumor size, grade, site, margins and disease status (primary/recurrent) were analyzed as potential prognostic factors. Tumor size (≥ 5 versus < 5 cm) and grade (G3–4 versus G1–2) significantly influenced disease-free survival and overall survival (but not local control) whereas margin status (positive versus negative) was the only significant parameter for local control (but without any impact on disease-free survival (DFS) and overall survival (OS)). The influence of these factors was particularly evident in patients who were treated with pre-operative RT because of the high incidence of large, high-grade tumors.

### Radiation therapy for retroperitoneal soft tissue sar comas: the impact of surgical resection extension and radiation dose on local control

A. De Paoli,[1] G. Boz,[1] G. Bertola,[1] R. Innocente,[1] A. Dedkov,[1] L. Balestrieri,[1] S. Frustaci,[1] A. Merlo,[2] M. G. Trovò & C. Rossi

*([1]CRO, I-33081 Aviano & [2]General Hospital, I-33170 Pordenone, Italy)*

Local tumor control is still the major problem for retroperitoneal soft tissue sarcomas (STS) because of the limited resectability rate and the high incidence of local recurrences after surgery. Between January 1985 and September 1995, 39 patients were treated at the CRO with either post-operative radiation therapy (RT) or RT alone for primary (26) or recurrent (8) retroperitoneal STS. This group of patients was selected from a series of 48 patients with retroperitoneal STS treated at our institute during the same period. Patients treated with resection alone (six), with doses $<36$ Gy (two) or patients with metastatic disease (six) were excluded from analysis. The median age was 52 years (range 16–74 years) and the male–female ratio was 19:15. Twelve patients (35%) had complete resection (no evidence of residual tumor) and 16 (47%) had partial resection (gross or microscopic residual tumor) whereas 6 patients (18%) had an unresectable tumor. A tumor size $<10$ cm (range 6–8 cm) was reported in 3 patients (9%), $\geqslant$ 10 cm (range 10–18 cm) in 26 patients (76%) and 5 (15%) had tumor size $>20$ cm (range 22–28 cm). High-grade tumors (G3–4) were reported in 7 patients (21%) and G2 and G1 tumors in 10 (29%) and 17 patients (50%), respectively. External RT was given with 8–16 MV Linac. Two or three target volumes were usually defined for treatment planning. RT dose was between 36 and 63 Gy (median 54 Gy). On April 30, 1997, the median follow-up was 34 months (range 20–139 months). The 5-year actuarial local control and survival rates were 52% and 42%, respectively. Local tumor control was achieved in 9/12 patients (75%) with complete resection versus 9/16 patients (56%) with partial resection and 0/6 patients with unresectable tumor. The RT dose appeared to influence tumor control with 1/8 patients (12%) having local control with a RT dose $\leq 50$ Gy compared with 17/26 (65%) with a dose $>50$ Gy. Local control was reported in 17/21 (85%) of the patients treated with resection and RT $>50$ Gy. The effect of tumor grade on the results was not demonstrated clearly. Major treatment complications were reported in 5/34 patients (12%) and consisted of small bowel obstruction (two), nephrosclerosis (one) and neuropathy (one). Long-term local control and survival appears to be associated with maximal surgical resection and higher RT doses. New treatment approaches, such as pre-operative RT aimed at increasing the resecability rate and innovative RT modalities aimed at escalating the dose to 50 Gy, including intra-operative RT, might be considered for the treatment of retroperitoneal STS.

### Dose-intensification of liposome-encapsulated daunorubicin with G-CSF as treatment for sarcomas and other solid tumors: phase I dose escalation study

G. D. Demetri, L. A. Stefanowicz, E. Geiger & S. Singer
*(Dana-Farber Cancer Institute and Brigham and Women's Hospital, Harvard Medical School, Boston, MA 02115, USA)*

Anthracycline-based chemotherapy remains a critical component in the cytotoxic treatment of sarcomas and other solid tumors, improved clinical outcomes have been associated with the delivery of more intensive doses of this class of chemotherapy. Although hematopoietic cytokines can mitigate the myelotoxicity of anthracyclines, dose intensification has been limited by non-hematologic toxicities such as severe mucositis and palmar–plantar syndrome. Targeted drug delivery is being explored to achieve high concentrations of chemotherapy selectively within tumor-related vasculature as a mechanism to minimize systemic dose-limiting toxicities of anthracyclines, as well as to improve the antitumor efficacy. Liposome encapsulation represents a promising technology to target drug delivery to the abnormal vasculature of tumor beds and to improve the systemic tolerance of anthracycline chemotherapy. Additionally, liposome encapsulation can dramatically alter the pharmacokinetics of drug delivery which may improve the therapeutic index. There are currently three different liposome-encapsulated anthracyclines which have either achieved regulatory agency approval for oncologic indications or which are in the late-stage clinical development. Our group decided to study the safety and efficacy profile of liposome-encapsulated daunorubicin (daunoxome) when administered at high doses using the hematopoietic support of G-CSF (filgrastim). We chose to study daunoxome because the safety profile at lower doses in other clinical trials appeared quite favorable, with no indication of mucosal or skin toxicities. Additionally, previous studies had shown that the small average diameter (45 nm) and the high stability in circulation of the unique unilamellar liposomes used in daunoxome result in a selectively high tumor accumulation of liposome contents. In our study, we have planned for dose escalation of daunoxome in a standard phase I intercohort fashion. The primary objective of the study is to assess the safety and tolerance of high doses of daunoxome for at least two cycles administered 3 weeks apart, with planned dose levels of 140, 180, 225, 250, 275 or 300 mg m$^{-2}$ given on one day. All treatment is outpatient. Patients with solid tumors are eligible, and prior anthracycline chemotherapy is not an exclusion criterion. To date, eight patients with sarcoma or breast cancer have been treated on the study (five at the first dose level of 140 and three at the next higher dose level of 180 mg m$^{-2}$). Clinical tolerance of treatment has been excellent, with no significant mucositis and no palmar–plantar erythema noted in any patient. Of the 18 cycles of chemotherapy delivered to date, no episodes of fever with neutropenia were observed in patients who were compliant with the protocol-specified G-CSF dosing (one episode of fever with neutropenia occurred in the study in a patient who omitted the G-CSF post-chemotherapy). All cycles have been delivered without treatment delays. One patient with prior doxorubicin treatment (230 mg m$^{-2}$ 4 months prior to study entry) treated with daunoxome at 140 mg m$^{-2}$ exhibited an asymptomatic decline in her left ventricular ejection fraction, but no other significant toxicities have been noted. Pharmacokinetic profiles are being monitored to assess the clearance of these high doses of anthracycline therapy. Further results will be presented at the meeting. In summary, our study is the first to administer such dose-intensive treatments with this novel liposome-encapsulated anthracycline. Daunoxome at high doses has been very well tolerated with G-CSF support and induces no significant mucosal toxicity.

### Phase II study of D-MAP + sargramostim in patients with advanced leiomyosarcomas

J. H. Edmonson, R. S. Marks & J. C. Buckner
*(Mayo Clinic, Rochester, MN 55905, USA)*

In an on-going outpatient study of advanced leiomyosarcomas, we have treated 29 patients using dacarbazine 750 mg m$^{-2}$, mitomycin 6 mg m$^{-2}$, doxorubicin 40 mg m$^{-2}$ and cisplatin 60 mg m$^{-2}$ given intravenously on day 0, with sargramostim 250 $\mu$g m$^{-2}$ every 12 h-subcutaneously on days − 6 to − 3 and on days 1–14 of each 4-week cycle. These patients have received a median of four cycles of this chemotherapy with median first cycle leukocyte and platelet nadirs of $5.6 \times 10^9$/l (range 0.7–$14.1 \times 10^9$/l) and $150 \times 10^9$/l (range 4–$298 \times 10^9$/l), respectively. While this regimen has been generally tolerable with only one patient refusing to continue therapy following the initial cycle, nausea and vomiting, weight loss and fatigue have been troublesome. Continued use of this regimen may produce severe myelosuppression. One patient died following four cycles of treatment with suspected treatment-related interstitial lung infiltrates with pleural effusion. Among the 17 patients with gastrointestinal leiomyosarcomas (GI stromal tumors) objective regression has occurred in 0/4 gastric tumors, 0/9 small bowel tumors and 1/4 colon tumors. In contrast, 5/7 uterine leiomyosarcomas regressed, as did 2/4 somatic (retroperitoneal and body wall) tumors and 1/1 cardiac leiomyosarcomas. Three of the five advanced uterine leiomyosarcomas patients who experienced objective regression have been rendered apparently disease free by interval surgery and are currently alive and well. This regimen is ineffective against GI stromal tumors, but its value against somatic uterine and cardiac leiomyosarcomas deserves further study. Useful therapeutic effects of this regimen may be observed in patients with advanced uterine leiomyosarcomas.

### Soft tissue sarcoma and Recklinghausen's disease

A. Emmermann, M. Peiper, R. Schwarz, H. J. Weh & C. Zornig
(*University Hospital Eppendorf, Hamburg, Germany*)

Malignant transformation of benign soft tissue tumors is seldom proven. However, in patients with Recklinghausen's disease and sarcoma it suggests itself. Therefore, we would like to characterize this group of patients and their therapy. Between 1986 and 1996 in the University Hospital of Hamburg, there were 13 patients with Recklinghausen's disease treated for malignant neurofibroma (12) or fibrosarcoma (1). Six women and seven men with an average age of 30 years (range 19–72 years). The localization of the tumors were lower extremity (four), head and neck (two), CNS (two) and upper extremity (one). The grading was dominated by $G^2$ and $G^3$ tumors. During the first operation, a complete resection (RO) could only be achieved in three cases, in six cases R1 (histological tumor rest) and in four cases R2 (macroscopic tumor rest) situations resulted. Adjuvant or additive therapy took place in nine cases (percutaneous radiotherapy, after-loading therapy and/or chemotherapy). At present three patients living free of tumor and 10 patients have died following multiple local recurrences or distant metastasis. Most of these tumors have a close relationship with large nerves, which results in reduced resectability. This might be the reason for the large number of relapses, and although many of the patients are young, it might be necessary for the first operation to be more radical including where necessary amputations.

### Isolated limb perfusion: a novel gene delivery system

B. W. Feig, M. M. Milas, K. Hunt & R. Pollock
(*MD Anderson Cancer Center, Houston, TX 77030, USA*)

*Introduction.* The use of isolated limb perfusion (ILP) as a treatment modality for extremity sarcomas in the adjuvant/neo-adjuvant setting has been hampered by local toxicity as well as a lack of consensus as to the appropriate chemotherapeutic agents. The major benefit of ILP is the ability to perfuse the tumor with significant doses of chemotherapy or other anti-neoplastic agents with minimal leakage into the systemic circulation. The present study was designed to examine the feasibility of gene therapy delivered by ILP in a rat hind limb sarcoma model.
*Methods.* ILP was performed in Fischer rats by cannulating the femoral artery and vein, isolating the hind limb circulation from the systemic circulation with the use of a tourniquet, and cycling the perfusate for 15 min at 2.4 ml min$^{-1}$. Leakage into the systemic circulation was 7.5% of the total perfusate concentrated in the isolated limb, as determined by perfusion with Tc99 m-tagged RBC. A syngeneic rat fibrosarcoma was implanted into the rat hind limb and allowed to grow to a diameter of 1 cm. Control tumor was implanted in the contra-lateral hind limb of each rat. The right hind limb was then perfused with the replication defective adenovirus Ad5LacZ, which contains the *Escherichia* coli $\beta$-galactosidase reporter gene under control of the CMV promoter. Animals were sacrificed and tumor was harvested at 48 and 72 h, and 7 days post-perfusion. Tissue samples were also obtained from muscle in the perfused limb, lung and liver at the time of necropsy. All tissue samples were stained with X-gal to evaluate for successful gene transfer to the tumor.
*Results.* There was no mortality amongst the rats undergoing perfusion. In addition, limb viability was preserved in all of the perfused hind limbs. The table below shows the number of perfused tumors in which $\beta$-galactosidase expression was observed.

| | 48 h | 72 h | 7 days |
|---|---|---|---|
| Number | 4 | 2 | 2 |
| Control | 0 | 0 | 0 |
| Perfused | 3 (75%) | 2 (100%) | 1 (50%) |

There was no $\beta$-galactosidase expression observed in the lung or muscle tissue samples. A minimal amount of ransgene expression was seen in the liver tissue.
*Conclusions.* This study demonstrates that ILP can be used as a method of delivering genetic constructs to tumors confined to the extremity. There was no evidence of local or systemic toxicity related to the recombinant adenovirus delivered by ILP. We are currently using this model to investigate the efficacy of therapeutic genes delivered to human sarcomas grown in the hind limbs of nude rats.

### T-Cell responses against the EWS/FLI-1 Ewing's sarcoma fusion protein

T. J. Goletz, C. L. Mackall, J. A. & L. J. Helman
(*National Cancer Institute, National Institutes of Health, Bethesda, MD 20892, USA*)

Ewing's sarcoma family of tumors (ESFT) share a specific t(11;22)(q24;q12) translocation that results in the generation of a chimeric transcription factor, EWS/FLI-1. This transcription factor, which is derived from the 3′DNA-binding domain of FLI-1 replacing the 3′RNA-binding domain of EWS, is thought to contribute to neoplastic transformation. In an attempt to develop novel immunotherapies for the treatment of these tumors, we noted that the translocation breakpoint generates a potential neoantigenic region with binding motifs for MHC class I and II molecules. To determine whether T-lymphocytes can recognize the breakpoint, we immunized experimental animals with dendritic cells pulsed with synthetic peptides corresponding to the breakpoint region. $CD8^+$ cytotoxic T-lymphocytes (CTL), which are capable of lysing peptide-pulsed targets, were generated. These CTL recognize two distinct $K^d$-restricted epitopes, one of which spans the translocation breakpoint. These data demonstrate that the translocation event in ESFT generates a neoantigen that can be recognized by CTL. Animal model studies are currently under way to optimize the generation of anti-EWS/FLI-1 CTL using retroviral transduction of dendritic cells, transduced tumor cells and DNA immunization. Additional studies are under way to characterize the efficacy of the response on tumor burden and survival. Based on the preclinical data, we have initiated a clinical trial to investigate the presence of, and/or the ability to generate antitumor responses in patients with recurrent or metastatic ESFT using tumor-specific peptide vaccination plus IL-2. Patients will be vaccinated during lymphocyte reconstitution following ablative chemotherapy in an attempt to preferentially expand antitumor T-cells during the T-cell expansion phase. Immune responses to be assayed include antigen-mediated proliferation, cytokine/production and/or tumor cell lysis. Preliminary results will be discussed.

**Elevated serum levels of vascular endothelial growth factor and basic fibroblast growth factor in patients with soft tissue sarcoma**

C. U. Graeven,[1] E. G. Achilles,[2] N. Andre,[1] N. Peiper,[2] C. Knabbe,[3] C. Zornig[2] & W. H. Schmiegel

*([1]Department of Medicine, University Hospital Knappschaft-skrankenhaus, Bochum, [2]Departments of General Surgery & [3]Clinical Chemistry, University Hospital Eppendorf, Hamburg, Germany)*

*Background.* Angiogenesis is essential for the progression of solid tumors. Tumor cells play an active role in angiogenesis by producing angiogenic factors. Among these, vascular endothelial growth factor (VEGF) and basic fibroblast growth factor (bFGF) have been shown to play a crucial role in neovascularization.

*Purpose.* To determine whether elevated circulating levels of VEGF and bFGF can be detected in sera from patients with soft tissue sarcoma (STS).

*Methods.* Eighteen healthy controls and 55 STS patients were enrolled in this study. In STS patients, sera were drawn prior to initial resection (IR) $n = 31$ or prior to wide re-excision (WR) 2–4 weeks after inadequate local excision $n = 24$. VEGF and bFGF levels were determined by ELISA.

*Results.* Mean levels were pg $ml^{-1}$ (range) for VEGF and bFGF in healthy controls were 167 (22–404) and 3 (1–9). In STS patients with IR, VEGF levels were 628 pg $ml^{-1}$ (range 64–2000 pg $ml^{-1}$) and bFGF levels 16 pg $ml^{-1}$ (1–50 pg $ml^{-1}$). Seventeen STS patients with evidence of disease after WR showed following serum levels: VEGF 515 pg $ml^{-1}$ (range 44 –2000 pg $ml^{-1}$), bFGF 26 pg $ml^{-1}$ (range 1–78 pg $ml^{-1}$). For 7 STS patients with no evidence of disease after WR the VEGF and bFGF levels were 350 pg $ml^{-1}$ (range 94–1150 pg $ml^{-1}$) and 23 pg $ml^{-1}$ (range 1–58 pg $ml^{-1}$).

*Conclusions.* Elevated VEGF and bFGF levels can be detected in sera from STS patients. Consecutive monitoring of VEGF and bFGF in the serum of STS patients might be a valuable new marker to monitor the tumor follow-up.

**Comparison of three methods of tendon attachment to an allograft/prosthetic composite of the proximal femur in an *in vivo* canine model**

G. F. Pluhar, J. P. Heiner, J. J. Bogdanske, J. Johnston, P. A. Manley & M. D. Markel

*(University of Wisconsin, Madison, Wisconsin, USA)*

*Introduction.* Despite the increasing use of resection and reconstruction of the proximal femur with allografts or allograft/endoprosthetic composites, inadequate or failed abductor tendon attachment to the allograft continues to cause functional impairment. Our study compared three methods of gluteus medius tendon attachment to an allograft/endoprosthetic composite of the proximal femur in a canine model.

*Materials and methods.* Twenty-four mongrel dogs were mismatched for major histocompatibility antigens using mixed lymphocyte reactions and divided equally into one of the three groups. Three methods of attachment were compared: (1) tendon—host tendon to allograft tendon with horizontal mattress sutures; (2) grip—host trochanter to allograft using a cable grip system; and (3) wrap—a thin shell of cortical host bone was wrapped around the allograft and secured with cerclage wires without disrupting the gluteus muscle attachment to the host bone. A total hip replacement was performed with the proximal 25% of the femur resected and reconstructed with an endoprosthesis first cemented into the allograft and then into the distal femur with antibiotic-impregnated polymethylmethacrylate. Serial radiography, dual energy X-ray absorptiometry (DEXA) and weight-bearing studies were performed bimonthly and just prior to euthanasia at 9 months. Radiographs were analyzed and graded based on the criteria developed by the International Symposium on Limb Salvage for fusion, resorption, fracture and graft shortening. Immediately following euthanasia, specimens were tested in tension to failure. Data were compared using analysis of variance (ANOVA) followed by a *post-hoc* *t*-test with differences considered significant at $p \leq 0.05$.

*Results.* Radiology: There were no significant differences among the groups for graft shortening. The tendon group had a higher fracture rate than the grip and wrap groups. The wrap group had less resorption and a faster fusion rate than the tendon and grip groups. DEXA: The wrap group had significantly greater BMD in zones 1 and 4, corresponding to the greater trochanter and calcar, respectively. Weight bearing: Only the wrap group reached values for pre-operative force at 9 months. Mechanical testing: There was a trend toward greater total energy and displacement to failure for the wrap group.

*Discussion.* Dogs reconstructed with the wrap method regained weight-bearing capabilities more rapidly, united the allograft and host bone faster with fewer fractures, and had less bone resorption based on radiographs and greater bone mineral density of the greater trochanter and calcar with DEXA than the other methods. The grip method performed better than the tendon-to-tendon method, but did not perform as well as the wrap method. The study indicated that any bone attached to the abductor mechanism should be saved if it does not compromise the margins of the tumor resection.

**Neo-adjuvant adriamycin and twice daily radiotherapy in adult soft tissue sarcoma**

J. P. Heiner, M. Mehta, H. Bailey, J. Fowler, T. Kinsella, D. Heisey & F. K. Storm

*(University of Wisconsin Hospital, Madison, WI 53792, USA)*

Thirty consecutive patients with intermediate or highgrade soft tissue sarcomas greater than 5 cm were entered into the study. The first 10 patients received 3 days intraveneous adriamycin (30 mg/day) followed by 28 Gy of radiation over 8 days. Three minor and two major wound complications occurred along with one local recurrence. Owing to this high complication rate in 1990, we began a study of the same dose adriamycin followed by 200 cGy bid for 8 days to a total of 32 Gy. This twice daily protocol predicts a decrease of 10% in the late complications by altering the biologically effective dose (60.7 $Gy_3$ to 53.3 $Gy_3$) to the tissues. Twenty consecutive patients had this protocol performed. Minimum follow-up was 24 months and median follow-up was 48 months. There was one local recurrence and one major wound complication, both in the same patient. Two of the 20 patients experienced delays in wound healing but did not require further surgery. Four patients developed metastatic disease during the study. Using the late effects scoring system for soft tissues and bones developed by the National Cancer Institute the twice daily radiation schedule had less early ($p \leq 0.01$) and late ($p \leq 0.02$) skin toxicity when compared to once daily radiation. Late muscle toxicity showed a trend ($p \leq 0.19$) in the twice daily group towards decreased late toxicity. We feel, in this pilot study, twice daily radiation shows promise with a low local recurrence rate and a statistically significant reduction in post-operative skin effects.

**Determinants of outcome after the development of pulmonary soft tissue sarcoma metastases**

T. J. Hieken, P. Menini & T. K. Das Gupta
(*Department of Surgical Oncology, University of Illinois at Chicago, Chicago, IL 60612, USA*)

Soft tissue sarcomas frequently metastasize to the lung, often selectively, and a small proportion of such patients may experience long-term survival. We studied 119 soft tissue sarcoma (STS) patients with lung metastases (LM) in an effort to discern factors that might reliably identify a potentially curable subset of patients. Fifty-four female and 65 male patients, ranging in age from 18 to 83 years, developed LM at a mean of 32 months (median 11 months) from the time of initial diagnosis. Overall, 19 of 119 patients survived $\geq 36$ months thereafter (crude 3-year survival 16%, actuarial 3-year survival 18%, mean survival 18.9 months and median survival 10 months). From numerous demographic and tumor features examined, only the time between initial diagnosis and development of LM ($p = 0.02$), unilateral versus bilateral LM ($p = 0.0005$), resection versus no surgical treatment ($p < 0.0001$) and administration of chemotherapy ($p = 0.03$) were significant variables. The latter three parameters retained significance in multivariate analysis. For the subset of 48 patients who underwent metastectomy with curative intent, 17 survived $\geq 36$ months (crude 3-year survival 35%, actuarial 3-year survival 38%, mean survival 32 months and median survival 18 months). For this subgroup of patients, we identified two markers of poor prognosis: multiple (versus solitary) LM and mutant p53 expression, assessed by quantitative ELISA assay, by the metastatic tumor. Only LM mutant p53 expression retained independent statistical significance ($p = 0.04$), suggesting that such tumors are biologically aggressive. These data confirm that long-term survival may be achieved for a subset of patients with pulmonary STS metastases, predominantly those with mutant p53-negative solitary or unilateral metastases who undergo surgical metastectomy. In this setting, mutant p53 may act to permit growth of chemo-resistant micrometastatic tumor cells.

**Reversal of drug resistance in osteosarcoma cells by variable resistance modifiers**

M. Hirata, K. Kusuzaki, H. Takeshita, S. Hashiguchi, H. Murata, Y. Hirasawa & T. Ashihara
(*Departments of [1]Orthopaedic Surgery, [2]Pathology, Kyoto Prefectural University of Medicine, Kyoto 602, Japan*)

*Introduction.* Multi-drug resistance (MDR) is one of the major problems in sarcoma chemotherapy. In this study, we investigated the effects of variable resistance modifiers (RMs) on adriamycin (ADM) resistance in osteosarcoma cells.
*Materials and methods.* We used the P-glycoprotein (Pgp)-positive, MDR murine osteosarcoma cell line (MOS/ADR) and the parental cell line (MOS) for this study. We tested six kinds of RMs, including immunosuppressors (cyclosporin A and FK506), a quinoline derivative (MS209), a steroid (methylpredonisolone), surfactants (Tween 20, Tween 80 and Polxamer 188), radiation and pulse electromagnetic field stimulation (PEMFs). To assess the effect of the RMs on ADM resistance, cells were incubated for 72 h in graded concentration of ADM with or without each of the RMs. The dose of ADM required to produce 50% growth inhibition ($IC_{50}$) was determined by use of the MTT assay. The sensitization by the RMs was expressed as the ratio of $IC_{50}$ without the RMs to $IC_{50}$ with the RMs.
*Results.* The Pgp-positive MOS/ADR 1 cells were 15-fold more resistant to ADM than the parental MOS cells. When FK506 was added at a final concentration of 3 $\mu$M to the MOS/ADR1 cells, 30-fold sensitization was observed. Similar effects were obtained at 3 $\mu$M MS209 or 0.03% Tween 80, whereas cyclosporin A and Tween 20 were less effective. In combination with radiation (1 Gy) and PEMFs (4 Gauss), three-fold sensitization was observed. No significant effects were found for methylpredonisolone or polxamer 188.
*Discussion.* The results demonstrated that ADM resistance in osteosarcoma cells could be reversed by variable RMs. However, the usefulness of these RMs remains to be proven because of the toxicity of RMs. MS 209 has now entered clinical trials. Surfactants have been used as solubilizing agent for drugs. PEMFs has been used for treatment of pseudoarthrosis. We hope that these methods will be helpful in overcoming MDR.

**Long-term treatment morbidity of extremity soft tissue sarcomas with intra-arterial chemotherapy, external beam radiotherapy and surgery**

H. Hoekstra, P. Nijhuis, E. Pras, D. Sleijfer, W. Molenaar & H. Schraffordt Koops
(*Groningen University Hospital, The Netherlands*)

*Introduction.* In the early 1980s the treatment of intra-arterial (ia) chemotherapy, pre-operative external beam radiotherapy (EBRT) and surgery was introduced in the limb-saving treatment of locally advanced soft tissue sarcoma (STS) of the extremity. The results and the short- and long-term morbidity were analyzed.

*Patients and methods.* Between 1983 and 1987, 11 patients with locally advanced STS were treated by continuous ia doxorubicin (20 mg $m^{-2}$ day $^{-1}$) for 3 consecutive days, pre-operative 35 Gy EBRT (10 × 350 cGy) and surgical resection. Non-radical resections received an addition 20–30 Gy EBRT.
*Results.* Limb saving was achieved in 10 patients (91%). During a median follow-up of 84 months (range 3–136 months), no local recurrences occurred and five patients developed pulmonary metastases (45%). Ten years disease-free survival and overall survival was 55%. Short-term morbidity: three patients, skin toxicity due to doxorubicin (27%); two patients, wound complications (18%) necessitating surgery. Long-term morbidity: radiation fibrosis with functional impairment in 60% of the survivors; one patient, stress fracture of the affected femur; one patient, severe progressive radiation-induced motor-sensory neuropathy of the sciatic nerve.
*Conclusions.* Although the combined treatment is feasible as a limb-saving treatment (91%) for locally advanced STS of the extremity without increasing the risk of local recurrence, this treatment should no longer be advocated due to the especially high long-term treatment-related morbidity.

**Primary non-Hodgkin's lymphoma of bone: a clinicopathological investigation of 61 cases**

F. H. Heyning,[1] P. C. W. Hogendoorn,[1] M. H. H. Kramer,[1] J. Hermans,[2] E. M. Noordijk[3] & Ph. M. Kluin[1]

(*Depts of [1]Pathology, [2]Medical Statistics and [3]Radiation Oncology, Leiden University Medical Center, Leiden, The Netherlands*)

*Introduction.* A retrospective analysis of patients presenting with primary lymphoma of bone (PLB) was performed to determine clinical factors affecting prognosis in relation to histological subtype and treatment outcome.
*Patients and methods.* From the files of the Netherlands Committee on Bone Tumors, 106 patients were retrieved who presented with PLB at one or more skeletal sites between 1943 and 1996. Reclassification of non-Hodgkin's lymphoma was performed according to the updated Kiel and REAL classification with the use of immunohistochemistry. Survival and prognostic factors were investigated.
*Results.* After reclassification, 61 patients were available with sufficient clinical data from moment of diagnosis and adequate follow-up. Most PLB were of the centroblastic-multilobated subtype (44%). All immunophenotyped PLB ($n = 38$) were of B-cell origin. PLB presented most often in the long bones (49%), with Ann Arbor stage I (46%), II (16%), IV (18%) and unknown (20%). Notwithstanding the heterogeneous treatments the 5-year overall survival was 60%. Complete remission was achieved in 29 patients with a 5-year disease-free period of 77%. Patients older than 60 years at presentation had a worse overall survival (76% versus 36% OS, $p = 0.0005$). Patients with bulky disease (> 5 cm diameter) had a shorter disease-free period (86% versus 58% DFP, $p = 0.04$).
*Conclusions.* PLB represents an uncommon bone tumor with a relatively homogeneous morphology and clinical behavior. As compared to other intermediate-grade lymphomas, the prognosis is favorable.

**Deletions of the tumor suppressor gene *CDKN2/P16* in human chondrosarcoma**

J. Asp,[1] S. Inerot[2] & A. Lindahl
(*[1]Department of Clinical Chemistry and Transfusion Medicine & [2]Department of Orthopedic Surgery, Sahlgren's University Hospital, S-413 45 Göteborg, Sweden*)

*Relevance to muscoskeletal conditions.* Understanding of the molecular events leading to chondrosarcoma is important for the prognosis and treatment of patients with this disease.
*Introduction.* The CDKN2 gene, which encodes the tumor suppressor protein p16, has been found to be altered in many forms of human cancers. p16 has its role in the dividing machinery of the cell, the cell cycle, where it functions as a negative regulator. When p16 is active, the cell is halted in the $G^1$ phase of the cycle. Inactivation of p16 by gene deletion or other events leading to a loss of functional protein will disrupt the regulatory pathway, including p16, and result in loss of cellular growth control. The aim of the present study was to investigate whether the CDKN2 gene was deleted or mutated in human chondrosarcoma.
*Methods.* Genomic DNA extracted from tumor cells and chondrocytes cultured in monolayer were used for amplification by polymerase chain reaction, Exons 1, 2 and 3 and their flanking regions of introns of the CDKN2 gene were amplified, as well as a fragment of the glyceraldehyde-3-phosphate dehydrogenase gene (G3PDH) used as a positive control. The amplified fragments of exons 1 and 2 were subjected to sequencing.
*Results.* Cells from 10 chondrosarcomas of different histologic grade and cells from 10 biopsies of hyaline cartilage obtained from patients during surgery were included in this study. In addition, lymphocytes were obtained from one of the tumor patients. All three exons of CDKN2 were deleted in three of the chondrosarcomas, i.e. in 30% of the tumors. In contrast, all of the samples of chondrocytes, as well as the sample of lymphocytes, contained all exons. One case of chondrosarcoma was found to contain a heterozygous position in codon 148 (exon 2). A nucleotide substitution from GCG to ACG had occurred, resulting in an amino acid change from alanine to threonine. This has previously been reported as a polymorphism. The same heterozygous position was also found in the DNA derived from the lymphocytes from the same patient. No point mutation or other minor alteration was found in the sequenced exons among the chondrocytes.
*Discussion.* The p16 coding gene CDKN2 is the most frequently altered gene among those encoding the components of the cell cycle. In this study, the gene was found to be deleted in 3/10 chondrosarcomas, but no sequence alterations were found except for a heterozygous position for a polymorphism. Our results indicate that p16 may play a role in the formation of tumors in human chondrosarcoma. A definite conclusion, however, cannot be drawn until a larger material has been studied and until the whole pathway including CDKN2, CDK4 and the retinoblastoma protein has been investigated totally in chondrosarcoma. There are also difficulties in examine tumors since even a minimal contamination of normal cells in the sample may hide a gene deletion or mutation.

**Combined resection, intraperitoneal chemotherapy and whole-abdominal radiation for the treatment of peritoneal mesothelioma**

M. L. Keohan,[1] J. Chabot,[3] K. Fountain,[3] K. H. Antman[1] & R. N. Taub[1]

(*Columbia University Divisions of [1]Medical Oncology, [2]Surgery & [3]Radiation Oncology, Columbia-Presbyterian Medical Center, New York, NY 10032, USA*)

Previous studies have suggested that multi-modality therapy prolongs survival in patients with peritoneal mesothelioma. The feasibility of combined resection, intraperitoneal (IP) chemotherapy and whole-abdominal radiation (RT) were evaluated in this pilot study. Patients were scheduled to undergo debulking with intraperitoneal catheter placement, followed 2–3 weeks post-operatively with five courses each of IP cisplatin 100 mg $m^{-2}$ alternating with doxorubicin 25 mg given weekly for a total of 10 courses. After re-exploration to document disease status and to resect residual disease, patients were to receive 3080 cGy to the abdomen and pelvis. Ten symptomatic patients (5 M/5 F), median age 48 years (range 45–71 years) were enrolled. Six patients had prior asbestos exposure. Two patients are currently awaiting second laparotomy; thus, eight patients are evaluable. Of these, four patients underwent surgical debulking and IP chemotherapy but did not receive planned RT. Four completed all three modalities of treatment. There have been no toxic deaths or renal complications. Three patients had grade 3 nausea during IP chemotherapy. One blocked catheter required discontinuation of chemotherapy, no other intraperitoneal complications occurred. Two of the four patients receiving RT developed transient grade 3/4 colitis. Two patients died of progressive disease. Six of the eight evaluable patients are alive, three have no evidence of progressive disease, two have intra-abdominal disease and one has a solitary metastasis to a left inguinal node. Surviving patients report decreased symptoms and improved quality of life after therapy. These results confirm the tolerability and efficacy of combined multimodality treatment and have led to our current phase I/II study of intraperitoneal chemotherapy/interferon, surgery and radiotherapy.

**Cancer risk data, blood and tissue collection program for familial soft tissue sarcomas**

W. G. Kraybill, J. Gibbs, B. McGrath, G. Anderson, P. Zhang, J. Brooks, C. Farrell, R. Perez, L. O'Malley, D. Driscoll & T. Weber
(*Roswell Park Cancer Institute, Buffalo, NY 14263, USA*)

The goals of this project are to collect blood and, when appropriate, tissue and to take extensive family and personal medical histories of selected proband with soft tissue sarcomas (STS). Blood, and if appropriate tissue, will be collected from selected family members. Eligibility of proband for this project are: (1) a family history of two or more individuals with STS; (2) a history of early onset of cancer in any family member with additional family members with STS; (3) multi-centric cancers in any family member with at least one family member having STS; (4) a family history of a hereditary disorder known or suspected of predisposing to STS; (5) known or suspected exposure to carcinogens and personal biopsy of STS; (6) two or more first-degree relatives of a proband with STS with the diagnosis of cancer. Peripheral blood cells and plasma will be banked from effected and non-effected first-degree family members of the proband and selected individuals from their families. Fixed tissues from participants who have had surgery either at or outside Roswell Park Cancer Institute will be collected, processed and stored through the Department of Pathology procurement plan. The ultimate goal is to make data and tissue available for cancer genetic research under Institutional Review Board approved research programs. Registry data and tissue will be made available to Roswell Park Cancer Institute researchers and selected outside scientists through individually arranged collaborations with this project's investigators.

**Binucleation induced by acridine orange in bone giant cell tumor cells**

K. Kusuzaki, H. Takeshita, M. Hirata, S. Hashiguchi,[1] H. Murata,[1] Y. Hirasawa[1] & H. J. Mankin[2]
(*[1]Orthopaedics, Kyoto Prefectural University of Medicine, Kyoto 602, Japan & [2]Massachusetts General Hospital, Massachusetts, USA*)

*Introduction.* There are many multi-nucleated giant cells in giant cell tumor of bone (GCT); however, it is still controversial how such multi-nucleation is created. It may be induced by cell fusion of mononuclear tumor cells, but nuclear division without cytoplasmic division may also be possible. We found many binuclear cells in isolated and smeared GCT cells. These binuclear cells possibly associate with the formation of multi-nuclear cells. Therefore, this study was undertaken to clarify the mechanism of binucleation in GCT using primary culture cells exposed to acridine orange (AO) which is a fluorescent vital staining dye for cytoplasms and the nucleus, and which inhibits mitosis.

*Materials and methods.* Culture cells were isolated from the explant of fresh materials from two GCTs (GCT1 and GCT2). These cells were cultured in D MEM with 10% fetal calf serum. After administration with 0.5 $\mu$g $ml^{-1}$ AO, at 0, 6, 24, 48, 96 h and 6 days, the following parameters were detected; 1, cell growth rate (GR); 2, frequency of hyperdiploid cells (%HDC) by DNA cytofluorometry; 3, mitotic index (MI); 4, BrdU labeling index (LI); and 5, frequency of binuclear cells (%BNC).

*Results.* Compared to control cells which were cultured in AO-free medium, the GR of both GCT cells exposed to AO was remarkably inhibited. MI was 0 from 24 h to 6 days. %HDC was increased at 24 h and was kept high until 6 days. LI was temporaly increased at 6 h, but was decreased at 48 h. %BNC was gradually increased.

*Discussion.* AO inhibited DNA synthesis and cell mitotic activity in cultured GCT cells, and it finally caused inhibition of cell growth. However, the frequencies of $G^2$ arrest cells and binuclear cells were increased. These results suggest that binuclear cells in GCT may be formed from $G^2$ arrest cells by amitotic nuclear division, not by mitosis without cytoplasmic division and not by cell fusion.

**Osteosarcoma in elderly patients: an unusual event for a bad prognosis disease. The Institut Gustave Roussy experience**

J. P. Delord, O. Merimsky, G. Missenard, K., Fizazi, D. Vanel, P. Terrier, M. Spielmann & A. Le Cesne

Osteosarcoma (OS) is a rare malignant tumor of bone that primarily affects teenagers. It occurs rarely in adult patients and seldom in elderly patients. In order to clarify the role of age in the prognosis of this disease, we performed a retrospective study on the behaviour of patients more than 40 years of age with OS who were treated in a single institution. Of 200 adult patients treated at IGR from 1976 to 1996, 31 were more than 40 years (15%). There were 16 males and 15 females. The median age was 56 years (range 40–84 years). Primary tumor sites included femur in eight, jaw and facial bone in six, pelvic bone in six, thoracic chest in four, humerus in three vertebra in two and tibia in two. Nine patients had synchronous pulmonary metastasis. Three patients developed OS in radiation fields after previous radiotherapy (breast cancer in two, Hodgkin's disease in one). One patients had juxta-cortical OS and two had Paget's disease. Twenty-eight patients were evaluable for survival. Initial treatment included surgery alone in two patients, surgery before chemotherapy in seven patients, radiotherapy alone in eight patients, radiotherapy after surgery in three patients and chemotherapy in a metastatic situation in six patients. In addition, two patients had neo-adjuvant chemotherapy before conservative surgery. The median survival was 1 year (range 1 month to 14 years). All but two patients died of metastatic evolution. One of the survivors had juxta-cortical OS and is alive 14 years after diagnosis. The second had femoral OS and underwent chemotherapy before conservative surgery (II B Rosen classification responder). This patient is alive 7 years after diagnosis. This retrospective study suggests that age is an unfavorable prognosis factor in OS. However, these results need to be modulated, first, by the inadequate therapeutic strategy due to the initial surgical approach, in these unexpected tumors and, second, by the use of non-aggressive adjuvant treatment due to the characteristics of these patients. As observed in younger patients, the incorporation of chemotherapy in the management of elderly patients with OS will indoubtably increase the prognosis of this disease.

### Local recurrence and subsequent survival in extremity soft tissue sarcoma

J. J. Lewis, D. H. Leung, E. S. Casper, J. Woodruff & M. F. Brennan
(*Memorial Sloan-Kettering Cancer Center, New York, NY 10021, USA*)

*Background.* The relationship between local recurrence and subsequent metastatic disease and death in patients with extremity soft tissue sarcoma (STS) is controversial. We analyze the correlation between local recurrence and subsequent survival in patients with primary extremity STS, treated and prospectively followed at a single institution.
*Methods.* Nine hundred and ninety-four patients with primary extremity STS were treated between July 1982 and December 1996 and then prospectively followed. The relationship of local recurrence with the development of subsequent distant metastasis and disease-specific mortality was analyzed adjusting for patient, tumor and pathological factors. Log-rank test and Cox proportional hazard regression were used to study the influence of local recurrence on distant metastasis and survival.
*Results.* The median follow-up was 3.97 years. During this time, 308 patients (31%) developed recurrence. Of these, 89 (9%) had local recurrence, 170 (17%) metastatic recurrence and 49 (5%) synchronous local and metastatic recurrence. On univariate analysis, there was a significant difference in metastasis ($p = 0.0001$) and disease-specific survival ($p = 0.0001$) between patients with and without local recurrence. In addition, on multivariate analysis, local recurrence was strongly associated with development of subsequent metastasis ($p = 0.0001$) and disease-specific death ($p = 0.0001$).
*Conclusions.* In this single institution analysis of a large group of patients with primary extremity STS local recurrence is strongly associated with development of subsequent metastases and disease-specific death. These data suggest that similar, or the same, biologic factors influence both local and systemic failure and that local recurrence is a poor prognostic factor for metastasis and survival.

### Regional induction chemotherapy and surgical resection for large or unresectable soft tissue sarcomas: evaluation of tumor necrosis, disease-free survival and local recurrence—is radiation therapy necessary for good responders?

R. Henshaw, D. Priebat, D. Perry, B. Shmookler & M. Malawer
(*Washington Cancer Institute at The Washington Hospital Center, Washington, DC 20010, USA*)

*Introduction.* Treatment of large and/or unresectable soft tissue sarcomas (STS) of the extremities remains a major challenge. Based upon experience with primary bone sarcomas, we hypothesized that induction chemotherapy would permit limb-sparing surgery by inducing tumor necrosis. In addition, we postulated that local control can be obtained for good responders without radiation therapy (RT).
*Strategy.* Twenty-four patients presenting with large and/or unresectable intermediate to high-grade STS of the extremities were prospectively enrolled in a neo-adjuvant (induction) protocol consisting of two cycles of intra-arterial (regional) cis-platin and intravenous adriamycin chemotherapy followed by limb-sparing surgery. Only patients who were deemed unresectable or of large tumor volume were treated in this study. Sixty-seven per cent of patients had high-grade MFH, with 41% involving the groin or popliteal fossa.
*Pathology.* Detailed quantitative pathologic analysis of surgical specimens was performed to determine per cent (%) tumor necrosis and assess surgical margins. If there was greater than 90% necrosis with negative margins, post-operative RT was not utilized. RT was given only if necrosis was less than 90% or for a positive margin. Good and poor responders were defined as those with greater and less than 90% tumor necrosis, respectively.
*Analysis.* Relapse- and disease-free survival was determined using the Kaplan-Meier method. Local recurrence was evaluated. Odds ratios were calculated based upon surgical margins and percentage of tumor necrosis for local recurrence as well as survival.
*Results.* Median follow-up was 51.5 months (range 19–133.4 months). The limb-salvage rate was 92% (22/24 patients), overall survival was 87.5% and disease-free survival was 70.4%. Local control was 92% (22/24 patients). Both patients with local recurrence were re-resected and remain alive and free of disease 29 and 85 months post-recurrence. Of the five patients who received RT, one died of metastatic disease, one is alive with metastasis, and the other three are disease free. Overall, five patients developed metastatic disease, of whom two remain alive. Median histologic tumor necrosis was 95% (range 5–100%). Close surgical margins were strongly associated with recurrent disease (one local and two metastatic) with an odds ratio of 10.5.

*Conclusions.* This study demonstrates that neo-adjuvant chemotherapy successfully shrinks most large and/or unresectable extremity STS allowing for limb-sparing procedures with excellent local control. The median percentage of tumor necrosis achieved (95%) is comparable to that seen in neo-adjuvant treatment of osteosarcoma. This study suggests that RT is not necessary for all STSs, and can be limited to those patients with poor histological response or close surgical margins. We recommend induction chemotherapy for all difficult or unresectable high-grade soft tissue sarcomas of the extremities.

**Comparison of postmorbid weight loss and albumin levels in patients with sarcomas versus other malignancies**

K. L. Melton, K. A Harris & B. E. Brockstein
(*The University of Chicago Medical Center, Chicago, IL 60637, USA*)

We have observed that patients with sarcoma seem to differ clinically from patients with carcinomas. One specific difference we have observed is the general 'health' of sarcoma patients seems to be good even at the end of life. We chose initially to explore weight loss and albumin levels, as a marker of overall nutritional status, in an attempt to quantitate measures of health. We retrospectively reviewed charts of 20 consecutively deceased patients from each of three categories: (1) sarcoma patients, excluding patients with intra-abdominal primaries; (2) gastrointestinal (GI) cancer patients; and (3) other carcinoma patients, including nine prostate cancer patients, seven breast cancer patients and four lung cancer patients, all without liver metastases. Weights and albumin levels were recorded for all patients using the time frames of baseline, mid tx and within an average of 9 weeks of death. The mean weight change for the three groups is as follows:

| | Baseline to mid (kg) | Mid to end (kg) | Total change (kg) |
|---|---|---|---|
| GI | − 1.66 | − 1.71 | − 3.06 |
| Other carcinomas | − 1.23 | − 3.27 | − 4.98 |
| Sarcoma | − 1.00 | − 0.170 | − 0.79 |

The mean albumin level is as follows:

| | Baseline to mid (g dl$^{-1}$) | Mid to end (g dl$^{-1}$) | Total change (g dl$^{-1}$) |
|---|---|---|---|
| GI | − 0.38 | − 0.38 | − 0.73 |
| Other carcinomas | − 0.24 | − 0.33 | − 0.57 |
| Sarcoma | − 0.22 | − 0.17 | − 0.41 |

In conclusion, our data demonstrate a trend toward a difference in weight loss and albumin level change for sarcoma patients compared to patients with GI cancers and other carcinomas without liver metastases. In order to better define the differences, we plan a prospective study to validate this data. Potentially, measures of cytokines could be made to determine if there is specific excretion from the tumor that may correlate with the weight loss. If such a factor is found, a specific drug therapy or treatment could be developed to maintain weight, improve outcome and hopefully add quality to a cancer patient's end of life.

**The reproducibility of the assessment of mitotic and proliferation indices in pediatric soft tissue sarcomas**

W. M. Molenaar, B. E. C. Plaat, E. R. Berends, M. F. Mastik & G. J. te Meerman
(*Depts of Pathology and Medical Genetics, University of Groningen, Groningen, The Netherlands*)

In the histological grading of soft tissue sarcomas, mitotic activity plays a major role. However, the seemingly simple counting of mitoses is fraught with difficulties, which may lead to substantial inter-observer variation. Therefore, antibodies (e.g. MIB-1) have been developed which decorate proliferating cells in tissue sections. The detection of thus labeled cells appears relatively easy, but the method is more cumbersome and it is uncertain whether proliferation and mitotic activity have the same biological significance. In the current study of 20 pediatric sarcomas, four experienced pathologists and four less experienced observers (pathology, resident, medical student, research technician and PhD student) assessed the mitotic index as number of mitoses per 2 mm$^2$ in H&E stained sections, thus mimicking routine pathology practice. In adjacent sections stained with immunoperoxidase stains for MIB-I, the proliferation index was assessed as the estimated percentage of labeled cells in the tumor cell population. Analysis of variance revealed that the variation between tumors explained 77% of the variation in mitotic indices in the group of experienced observers, as compared to 49% in the less experienced group. The proliferation indices showed less variation between observers than the mitotic indices. The variation between tumors explained 64% of the variation in proliferation indices in the experienced group and 71% in the less experienced group. No correlation was found between mitotic and proliferation indices in the various tumors. The results suggest that training is an important factor in the reliability of mitotic counting. The use of proliferation markers has a higher reproducibility, especially in less experienced observers. However, for clinical use it has the disadvantage of being the more expensive and more time-consuming technique; moreover, the biological significance of proliferation is as yet unestablished and may differ from that of mitotic activity.

**Rhabdomyosarcoma of the cystic duct in a child**

N. P. Padiyar
(*Wayne State University School of Medicine, Detroit, MI 48201, USA*)

Neoplasms of the hepatobiliary systems in either adults or children are very rare. Malignant tumors of the bile duct and the gall bladder are even rare. There are a handful of these cases reported in the literature. Embryonal rhabdomyosarcoma of the cystic duct in a child is reported here. Although these tumors are exceedingly uncommon in this age group, they are morphologically similar to embryonal rhabdomyosarcomas in other organs. The case discussed here is an embryonal rhabdomyosarcoma of the cystic duct, presented with obstructive jaundice caused by the tumor. The diagnosis was based on clinical findings proven by tissue diagnosis. The biopsy showed a spindle cell neoplasm with mild pleomorphism and abundant mitosis. These findings were supported by immunohistochemical staining. The large mass encircling the cystic duct was excised following therapy The post-operative course was unremarkable.

**Feasibility and activity of continuous infusion high-dose ifosfamide in advanced and/or metastatic, pretreated soft tissue sarcomas: a phase II study**

R. Palumbo,[1] B. Castagneto,[2] C. Gatti,[3] P. Raffo,[1] G. Villani[1] & S. Toma[1]

*([1]National Institute for Cancer Research, Department of Medical Oncology, University of Genoa, I-16132, [2]Department of Oncology, Casale Monferrato Hospital, Alessandria, & [3]Department of Oncology, Sanremo Hospital, Italy)*

*Background.* Ifosfamide has important activity in pretreated soft tissue sarcoma (STS), and recent data support a clinically significant dose–response relationship for this agent. Continuous infusion (ci) administration and hematopoietic support have been shown to allow dose intensification regimens, by reducing both hematologic and non-hematologic toxicities. The optimal dose and schedule of ifosfamide when given at high doses remains to be identified. In a previous phase I study, we demonstrated the feasibility of a ci high-dose ifosfamide (HDI) regimen in an ambulatory setting on patients with advanced solid tumors. The present phase II study was performed to verify the antitumor activity and toxicity of such a schedule in patients with advanced pretreated STS.
*Patients and methods.* Thirty-eight patients with advanced and/or metastatic STS, all pretreated with anthracycline with or without standard-dose ifosfamide, were treated. Ifosfamide was given by ci at a dose of 3.5 g $m^{-2}$ $day^{-1}$ over 4 consecutive days, with equidose mesna uroprotection over 5 days. G-CSF was added at a dose of 200 $\mu$g $m^{-2}$ $day^{-1}$ subcutaneously from day 6 to day 12. Cycles were repeated every 3 weeks, in an outpatient setting.
*Results.* A total of 159 cycles of therapy were given (median 4 per patient, range 3–6). Overall treatment compliance was satisfactory. Major toxicity was hematologic, with six febrile neutropenic episodes requiring parenteral antibiotics. Both renal and neurologic toxicity were mild. An overall response rate of 39% was observed (95%, 26%–55%), with 1 complete and 14 partial remissions. Twelve out of 15 patients (80%) with lung metastases as the only site of disease responded to therapy, six of whom underwent successful salvage surgery. All but one of the responder patients had previously received standard dose ifosfamide. Median response duration was 9 months (range 5–21 + months); the overall median survival ranged from 6 to 30 + months (median 13 months).
*Conclusions.* Our results show that HDI is an active regimen in advanced and/or metastatic anthracycline-pretreated STS, further confirming its ability to circumvent resistance to both previous anthracycline and low-dose ifosfamide therapy. Future clinical trials should be aimed at evaluating the impact of different administration schedules on clinical response and outcome (i.e., repeated short daily infusion versus ci). The potential role of HDI as front-line chemotherapy as well as in the adjuvant treatment of STS needs to be investigated in randomized trials.

**Gastrointestinal autonomic nerve tumor (GANT): a clinicopathological and immunohistochemical study of a case**

F. Pari,[1] E. Aitini,[1] R. Fante,[2] F. Colpani,[2] C. Pulica,[3] F. Adami,[1] D. Zamagni[1] & F. Smerieri[1]

*(Departments of [1]Medical Oncology, [2]Pathology & [3]Surgery Departments, C. Poma Hospital, Mantova, Italy)*

GANT is an uncommon specialized form of gastrointestinal stromal tumor (GIST) whose immunohistochemical features support a myenteric plexus derivation. A 46-year-old male presented complaining of abdominal pain and strain. Patient examination revealed ascites devoid of malignant cells. A computed tomography (CT) scan showed a pelvic mass but endoscopic examination was negative for neoplasm and biochemestry was normal. The patient underwent laparotomy: a large ($40 \times 14 \times 7$ cm) tumor arose from the sigma, filling the pelvis and infiltrating the bladder and the gastric antrum. Histology showed a mesenchymal epithelioid tumor without significant pleomorphism. Cells had hyperchromatic vescicular nuclei and eosinophilic large, sometimes vacuolated, cytoplasm. Cellular arrangement was patternless and lymphoid aggregates coexisted. Mitotic figures were $5 \times 50$ HPF. A wide range of immunohistochemical stains was performed: vimentin, CD34, neuron-specific enolase (NSE) were positive; chromogranin, S-100 protein, smooth-muscle actin, desmin, LCA, EMA were negative. The tumor was diagnosed as GANT and confirmed by two different consultant pathologists. Eight months later the patients developed an omental recurrence. GANT originated from the myenteric nervous system. Neural differentiation has been postulated on ultrastructural features and on reactivity with CD34, NSE and vimentin. CD34, a myeloid progenitor cell antigen, is expressed in endothelial and non-committed mesenchymal cells. Expression of CD34 in GANT suggests the possibility that this tumor derives from a precursor element of nerve plexus differentiation. GANT aggressive behavior correlates with tumor size ($> 10$ cm) and/or mitotic figures ($> 5 \times 50$ HPF). To our knowledge, this is the first case of a large bowel GANT described in the literature.

**Survival and local recurrence in soft tissue sarcomas of the extremities and trunk: a study of 101 patients with primary tumors treated within 6 years**

M. Peiper, D. Zurakowski & C. Zornig
*(Department of Surgery, University Hospital, Hamburg, Germany & Department of Research Computing, Children's Hospital, Harvard Medical School, Boston, USA)*

*Background.* Few articles have analyzed prognostic data regarding survival and local recurrence in a large series of primary soft tissue sarcomas (STS) of the extremities and trunk operated on within a short period of time.
*Methods.* A consecutive series of 101 patients with primary extremity and truncal sarcomas from 1988 to 1993 was analyzed using logistic regression and survival analysis. Patients were treated by the same surgeon using the same multi-modality treatment plan.
*Results.* Fifty males and 51 females had a median age 49 years. Three patients presented with positive regional lymph nodes and two with synchronous distant metastases. Seventy-eight tumors were located at an extremity, while 23 were truncal STS. In 97 patients, the tumors were resected completely, the remainder were resected R2. Thirty-two tumors were highly differentiated (G1) 32 moderately (G2) and 37 tumors were graded G3. Thirty-two tumors were smaller than 5 cm (T1) and 69 tumors were larger than 5 cm (T2). Radiation therapy was performed in 44 patients: in 12/32 patients with low-grade sarcomas, 10/32 with intermediate-grade sarcomas and 22/37 with high-grade sarcomas. While 41 patients, were treated adjuvantly, radiotherapy (RT) was administered in three patients with R2 resections. Grading (G3 versus G1, $p < 0.01$), tumor size ($p < 0.05$), distant metastases ($p < 0.01$) and resection quality (R1 versus RO, $p < 0.01$;

R2 versus RO, $p < 0.05$) were univariate predictors of mortality. Stepwise logistic regression revealed that multivariate predictors of mortality consisted of tumor grade ($p < 0.0001$), presence of lymph nodes ($p < 0.001$), resection quality ($p < 0.01$) and location ($p < 0.01$). Patients with G3 tumors survived significantly less often than patients with G1 tumors ($p = 0.01$). Analysis of local recurrence revealed positive regional lymph nodes and resection quality as the strongest risk factors (both $p < 0.05$).
*Conclusions.* An optimized surgical treatment of the primary tumor manifestation augments survival time and reduces the rate of local recurrence. Post-operative RT is indicated in cases where no ideal RO situation can be achieved.

**Human colorectal cancer-derived epithelial and mesenchymal cells in co-culture**

L. Picariello, G. Fiorelli, S. Benvenuti, S. Zecchi Orlandini, L. Formigli, L. Messerini, A. Calzolari, M. L. Brandi & F. Tonelli
(*Department of Clinical Physiopathology, University of Florence, Florence, Italy*)

The interactions between mesenchymal and epithelial cells are known to play an essential role in several pathological processes including the human colorectal adenocarcinoma. The synthesis of a peritumoral extracellular matrix can provide a permissive environment for tumor development and spreading, contributing to tumor progression. To add information on the interactions between colorectal epithelial and mesenchymal cells, we investigated the role of adhesion proteins such as laminin, fibronectin, vitronectin and collagen type IV on the adhesion of a human colon adenocarcinoma cell line (HCT8 cells) to fibroblasts derived either from human colorectal cancer (CKF) or normal colon (CNF) specimens in co-culture. The proliferative activity of CKF, measured by cell counting was significantly ($p < 0.05$) higher than that of CNF. The HCT8 cell adhesion, evaluated by a colorimetric method, was significantly higher when the cells were co-cultured with CKF than with CNF. Morphological evaluation of the co-cultures by scanning electron microscopy confirmed the higher adhesion of HCT8 cells to cancer-derived mesenchymal cells. The higher proliferative activity of CKF and the preferential adhesion of HCT8 cells to these fibroblasts would suggest an increased production of matrix proteins by CKF. Inhibition studies with specific monoclonal antibodies against laminin, fibronectin, vitronectin and collagen type IV showed that CKF synthesized more adhesion proteins than CNF. In addition, CKF exhibited a higher expression of cadherins in comparison with CNF. The present findings show: (a) higher adhesion of HCT8 cells to fibroblastic monolayers derived from colorectal cancer than to normal colon fibroblasts and (b) a higher expression of cadherins in tumor-derived fibroblast monolayers. The different synthesis of extracellular matrix proteins and cadherins by tumor fibroblastic cells could offer a means to understand both tumor progression and spreading.

**Genetic changes in non-myxoid liposarcomas**

B. E. C. Plaat, P. R. A. Sars, J. Sagrudny, W. M. Molenaar, R. M. Bohle & E. v. d. Berg
(*Departments of Pathology,, Surgery and Medical Genetics, University of Groningen, Groningen, The Netherlands & Department of Pathology, Justus-Liebig University Giessen, Germany*)

In myxoid liposarcomas and their poorly differentiated counterparts, round cell liposarcomas, the occurrence of a chromosomal translocation between chromosomes 12 and 16, i.e. t (12; 16) (q13; p11), has been well established. At a molecular level a TLS/FUS-CHOP fusion is involved. Much less is known about genetic alterations in non-myxoid liposarcomas, although ring chromosomes have been described in well-differentiated liposarcomas. The current study reports genetic changes in six cases of non-myxoid liposarcomas and two cases with a presumptive, but uncertain, diagnosis of (non-myxoid) liposarcoma. All three well-differentiated liposarcomas showed near-diploid karyotypes with ring chromosomes in two cases and telomeric associations in one of these two, as reported in the literature. The third case showed an addition of the short arm of chromosome 16, involving band p11, but no changes in 12q13. Two dedifferentiated liposarcomas showed aneuploidy, whereas one case was near-diploid. All three showed very complex karyotypes. One case with a tentative diagnosis of round cell liposarcoma showed a near-diploid karyotype with complex chromosomal changes including a ring chromosome. The final case, tentatively diagnosed as pleomorphic liposarcoma, revealed a complex karyotype ranging from haploid to octaploid without 'specific' changes. None of the cases showed a t (12;16) or a TLS/FUS-CHOP fusion transcript on RT–PCR. The latter findings are in keeping with the literature on non-myxoid liposarcomas and argue against a diagnosis of round cell liposarcoma in one of the cases with uncertain histological diagnosis. The presence of a ring chromosome in this case is in line with a diagnosis of liposarcoma, although the histology does not resemble a well-differentiated subtype. The karyotype in the case tentatively diagnosed as pleomorphic liposarcoma did not reveal any changes 'characteristic' of liposarcoma, or of another specific soft tissue tumor type.

**Synovial sarcoma in children and adolescents: 30 years of experience with multi-modal therapy**

R. B. Raney, M. F. Okcu, N. Jaffe, M. Choroszy & A. Cangir
(*The University of Texas, MD Anderson Cancer Center, Houston, TX 77030, USA*)

We reviewed the charts of 39 children and adolescents with synovial sarcoma; 22 were male, 17 were female. Median age at diagnosis was 11.4 years (range 5.7–16.9 years). Twenty-four were caucasian, 10 were hispanic, 4 were African American and 1 was oriental. Median tumor diameter was 5.5 cm (range 1–17 cm). According to the IRS clinical grouping system, 24 had group I (localized, completely removed), 10 had group II (micro-residual), 2 had group III (gross residual) and 3 had group IV (metastatic) disease. Primary sites were: lower extremity 21, upper extremity 9, trunk 5, and head and neck 4. Histologically 20 were biphasic, 18 were monophasic and 1 was not specified. The treatment varied according to the time of diagnosis and tumor size/location, as follows: surgery (S) and chemotherapy (C) (pre- or post-operatively), 15; S, radiotherapy (R) and C, 14; S and R, 6; S only, 3, and C only, 1. With a median follow-up of 6.8 years, the survival rate is 74% (29/39): group I, 22/24; group II, 7/10; groups III + IV, 0/5. One patient died from telangiectatic osteosarcoma secondary to radiotherapy. All of the patients who were group III or IV died in spite of different modes of treatment. Of the nine who died from synovial sarcoma, eight were more than 10 years old and had maximum tumor diameter of $> 5$ cm at the time of diagnosis; six had biphasic tumors and three monophasic. Survival by histologic type was 83% (15/18) with monophasic tumors and 65% (13/20) with biphasic tumors ($p > 0.05$). Of the 10 group II patients with micro-residual disease after S, three were treated with C successfully; of the other 7 who received R (7) + C (4), four are alive and disease free. Post-operative C and R seemed to be effective for patients with microscopic disease, although the number of patients in this group was small. Gross total removal of the primary tumor was necessary for long-term survival. Proof of efficacy of non-surgical treatment, especially C, will require a randomized comparison with larger numbers of patients.

### Dose-intensive chemotherapy with stem cell support for adult patients with advanced soft tissue sarcoma: a phase I study

P. Reichardt,[1] J. Tilgner,[1] M. Y. Mapara,[1] P. Hohenberger[2] & B. Dörken[1]

*([1]Department of Hematology, Oncology and Tumorimmunology & [2]Department of Surgical Oncology, Robert-Rössle-Klinik, Virchow-Klinikum, Humboldt University, D-13122 Berlin, Germany)*

Dose-intensive combination chemotherapy with anthracyclines and ifosfamide (IFO) currently seems to be the most promising approach for patients with advanced soft tissue sarcoma. In a previous phase II study using IFO 2.5 g $m^{-2}$ $day^{-1}$ as a continuous infusion, days 1–5 and epirubicin (EPI) 45 mg $m^{-2}$ $day^{-1}$ as a continuous infusion, days 2 and 3 repeated every 3 weeks, a response rate of 52% with 22% complete response (CR) was seen in 46 patients. Due to these encouraging results, a phase I study using further increased doses of EPI and IFO with hematopoietic stem cell support was started. After one induction cycle of IFO/EPI at the above mentioned dosage, stem cells were harvested (1–3 leukaphereses with a median total of $14 \times 10^6$ CD 34 + cells/kg) and up to four cycles of dose-intensified IFO/EPI, followed by stem cell support ($> 2 - 10^6$ CD 34 + cells/kg) were given every 3 weeks. Cumulative doses of IFO and EPI are escalated in a stepwise manner in groups of three patients. up to 17.5 g $m^{-2}$ and 150 mg $m^{-2}$ per cycle, respectively. So far, 15 patients (M:F = 10:5, median age: M = 32 years, F = 49 years) have been entered and a total of 40 dose-intensified cycles have been applied. Duration of leukopenia and thombocytopenia was up to 6 and 4 days, respectively, in all cycles. Severe non-hemotological toxicity so far consisted of reversible grade 4 nephrotoxicity (1 patients, dose step 2) and reversible grade 4 hepatotoxicity (1 patients, dose step 3). Application of up to 5 cycles of dose-intensified chemotherapy with stem-cell support every 3 weeks seems feasible without cumulative hematotoxicity Recruitment and dose escalation are on-going and final results will be presented.

### Prospective trial of conservative surgery and selective use of radiotherapy for AJCC T1 extremity and trunk soft tissue sarcomas

P. Respondek, A. Pollack, B. Feig, A. Yasko, K. Hunt, M. Johnson, J. Griffin, K. Zagars, R. E. Pollock & P. W. T. Pisters

*(The Sarcoma Center, University of Texas, MD Anderson Cancer Center, Houston, Texas, USA)*

*Background.* Recent reports have suggested that some patients with T1 (< 5 cm) primary soft tissue sarcoma (STS) can be treated by surgery alone. In an effort to better define which patients can be treated by surgery alone, we have initiated a prospective trial evaluating the use of conservative surgery with selective use of post-operative external beam radiotherapy (RT) for patients with AJCC T1 extremity and trunk STS.
*Methods.* Patients with T1 primary extremity and trunk STS were treated with conservative, limb-sparing surgery and careful assessment of microscopic surgical margins. Patients presenting with primary STS in situ underwent conservative surgery with assessment of microscopic margins at MDACC. Patients referred to the MDACC following pre-referral excision with equivocal or frankly positive microscopic margins underwent re-excision of the scar and tumor bed ($n = 2$). Patients referred following pre-referral excision where excision was associated with unequivocal negative microscopic margins were treated with observation ($n = 2$). Post-operative RT was employed for all patients with positive microscopic margins following re-excision ($n = 2$).
*Results.* Nineteen patients have been entered on this protocol since 2/1/96. STS were located in the lower extremity ($n = 9$), upper extremity ($n = 6$) and trunk ($n = 5$). Ten patients (50%) had high-grade lesions. Five patients (26%) had deep lesions. The majority of patients were treated with surgical resection or re-excision alone ($n = 17$); two patients were treated with post-operative RT for positive microscopic surgical margins following resection at the MDACC. Median follow-up is short (11 months, range 3–18 months). Two patients have experienced local recurrences at 2 and 12 months, respectively. There have been no distant recurrences. Neither patient received post-operative RT. Both patients who experienced local recurrence had lesions located in the groin and at the knee—wide local excision with satisfactory gross margins can be difficult.
*Conclusions.* Additional patients and longer follow-up will be required to determine which, if any, subsets of patients with small STS can be treated by surgery alone.

### Results from a new multi-disciplinary sarcoma clinic

L. M. El-Helw, P. C. Lorigan, J. Goepel & M. H. Robinson

*(YCRC Department of Clinical Oncology, Weston Park Hospital, Sheffield S10 2SJ, UK)*

*Objective.* Prospective data have been collected for all new patients seen at a specialist multi-disciplinary sarcoma clinic since its inception in October 1992 with respect to pattern of referral, clinical presentation and management. The numbers referred have been compared to population-based (1.7 M) data provided by the Trent Cancer Registry.
*Patients.* Three hundred and fifty patients were registered with a diagnosis of sarcoma in North Trent during this period. Two hundred and seventeen patients, 104 males (47.9%) and 113 females (52.1%), were seen in the multi-disciplinary clinic, 151 (70%) of these were new cases. The median age was 60 years (range 15–91 years). Lower limb and truncal sites predominated with 71 patients (32.7%) in each group. The most common sites seen were the anterior thigh (34 patients), retroperitoneum (20) and chest wall (18). Sixty-nine (31.8%) patients were referred by general surgeons, 57 (26.3%) by GPs, 11 (5.1%) by physicians and 4 (1.8%) by thoracic surgeons. Forty-seven (21.7%) patients were referred for a pre-operative decision on treatment and 67 (30.8%) after surgery. The remainder were seen with local recurrence (17%), metastasis (7.4%), both local recurrence and metastasis (2.3%), or for an opinion or follow-up. In the population, leiomyosarcoma predominated (26%) with 11% liposarcoma, whereas the most common histological types in patients attending the clinic were liposarcoma in 32 patients (16.2%), leiomyosarcoma in 29 (14.7%) and MPNST in 16 (8.1%). The majority of patients (51%) presented with stage III disease, the other stages were less common; stage I (22%), stage IV (16.4%) and stage II (10.6%). Only 45 patients (20.1%) had no surgical treatment and 5 patients (2.3%) underwent metastasectomy. Six out of 115 patients (5.2%) with limb sarcoma had an amputation. Thirty-five out of 67 patients (52.2%) referred for treatment after primary surgery required a second operation because of residual macroscopic disease (18), residual microscopic disease (9) or because there was doubt about the surgical margins (8). Local recurrence occurred in 56 patients (25.8%), 43 (76.8%) of them were treated radically, 13 (23.2%) were treated palliatively. The median time to local recurrence was 546 days for STS and 757 days for gynaecological sarcoma. Metastasis occurred in 65 (30%) patients. The most common site was lung (47.7%) and the median time to first metastasis was 462 days for soft tissue sarcoma (STS) and 620 days for gynaecological sarcoma.

*Conclusions.* Overall, 43% of patients in North Trent with a new diagnosis of sarcoma were referred to the sarcoma clinic, 50% of whom were referred at, or around, the initial diagnosis. Moreover, only 20% were seen before primary treatment was carried out. However, it appears that there was improvement in the pattern of referral after June 1992 as 37 151 (24%) patients with STS sarcoma were referred to the sarcoma clinic before primary treatment, while only 2 patients were referred to the sarcoma clinic before primary treatment prior to June 1992. There is a need to improve the pattern of referral.

### Diffuse aggressive pigmented villonodular synovitis of the knee: a unique surgical approach combined with radiotherapy

C. Rubert, A. Kelly & M. Malawer
(*Washington Cancer Institute at The Washington Hospital Center, Washington, DC 20010, USA*)

*Introduction.* The diffuse form of pigmented villonodular synovitis (PVNS) with both intra- and extra-articular extension has been recently termed aggressive synovitis due to its high rate of local recurrence. It is often confused with peri-articular sarcomas. To date, it has been difficult to treat with surgery alone. The purpose of this study was to develop a new strategy to treat the diffuse aggressive type of PVNS.

*Methods.* Four patients with recurrent diffuse aggressive PVNS involving both anterior and posterior compartments of the knee were treated with a combination of surgery (using a two-stage approach) and radiotherapy (RT). All patients had failed previous synovectomies. Magnetic resonance imaging (MRI) was used to delineate extent of disease in both compartments. All patients had both intra-and extra-articular disease involving anterior and posterior compartments with invasion of the popliteal space. All patients underwent open radical anterior synovectomy followed by a period of post-operative rehabilitation. All patients were required to regain full motion prior to a second-stage open posterior synovectomy. All patients received moderate dose RT (3000 cGy in 15 fractions).

*Results.* All patients achieved full extension and an average of 100° of flexion. All patients are full weight bearing without recurrent swelling or pain. At a median follow-up of 1 year, there were no recurrent effusions or local recurrences. There were no hemarthroses, skin flap problems, nerve palsies or contractures. One patient developed a post-operative hematoma which resolved after drainage and a short period of immobilization. There were no complications of RT (i.e. no problems with wound healing, joint stiffness or contracture).

*Conclusions.* Although benign, the diffuse form of PVNS behaves aggressively, has a high rate of local recurrence and may result in joint destruction. The differential of a peri-articular sarcoma is important. Limited arthroscopic synovectomy is recommended only for biopsy. Open radical synovectomy from both anterior and posterior approaches is required to remove extra-articular disease. This strategy of a two-stage approach minimizes the morbidity often encountered following open synovectomy. RT is recommended following surgical resection of all gross disease. To date, there have been no recurrences with excellent functional results. In patients with diffuse aggressive PVNS involving both anterior and posterior compartments with extra-articular involvement, we recommend a two-stage approach to surgical excision followed by RT.

### 'Telangiectatic' (high-grade hemorrhagic) soft tissue sarcomas—an analogue to telangiectatic bone sarcomas: diagnosis, treatment and outcome

C. Rubert & M. Malawer
(*Washington Cancer Institute at The Washington Hospital Center, Washington, DC 20010, USA*)

*Introduction.* High-grade soft tissue sarcomas which present as a large 'hematoma' or hemorrhagic mass are rare but tend to have a distinct clinical presentation, pathologic findings, response to chemotherapy and propensity for early dissemination. Like telangiectactic osteosarcomas, diagnosis can be difficult. We report seven patients with this unique entity.

*Patients and methods.* Seven patients with high-grade sarcomas who presented with large hemorrhagic masses were evaluated. There were three males and four females. All patients presented with a diagnosis of hematoma based upon ultrasound computed tomography (CT) or (MRI), aspiration and clinical exam. The tumor site was the thigh in five patients and the calf in two patients. MRI and CT scans were difficult to interpret and suggested large blood-filled cavities with surrounding edema. The wall (pseudocapsule) was of variable thickness with enhancing nodules and septae along the margins. Difficulty in establishing the diagnosis of malignancy occurred in all patients. Attempted core needle biopsies and aspirations yielded nondiagnostic bloody fluid. Open biopsy was necessary in all patients to obtain a satisfactory amount of tissue for diagnosis. Five of the seven patients underwent neo-adjuvant chemotherapy utilizing adriamycin and cis-platin combinations. Limb-sparing surgery was performed on 6/7 patients. Post-operative radiotherapy was used in one patient with a poor histological response (< 90% tumor necrosis) and one patient who did not receive neo-adjuvant chemotherapy and had a close surgical margin.

*Pathology.* All gross specimens revealed hemorrhagic tissue containing large, blood-filled cavities. Viable tumor cells were concealed by tremendous amounts of hemorrhage and necrosis, or present only within the walls of the tumor pseudocapsule. MFH was diagnosed in four patients, leiomyosarcoma in two patients and synovial sarcoma in one patient.

*Results.* The median follow-up was 8 months (range 6–10 months). There were no local recurrences. One patient (with synovial sarcoma) presented with metastatic disease. To date, no other patients have developed metastatic disease.

*Summary.* Great caution must be used in distinguishing hematomas from hemorrhagic soft tissue tumors. The similarity in diagnostic difficulty, CT and MRI findings, pathology and grade of tumor suggests that there is a subgroup of soft tissue sarcomas (MFH and leiomyosarcomas) which presents with clinical, pathological and radiological similarity to the hemorrhagic counterpart of bone sarcomas (telangiectatic osteosarcoma). This represents a unique clinical entity or subgroup of soft tissue sarcomas. Although diagnosis is difficult, hemorrhagic sarcomas tend to respond to neoadjuvant chemotherapy, thereby permitting a limb-sparing surgery.

**Unplanned excision of soft tissue sarcoma of the extremities**

K. A. Siebenrock & R. Ganz
(*Orthopaedic Surgery, University of Bern Inselspital, CH-3010 Bern, Switzerland*)

Fifteen patients were reviewed who were referred to our department after unexpected excision of a soft tissue sarcoma of the extremities. Seven (47%) tumors were located in the muscle, deep to the fascia and seven lesions exceeded 5 cm in size. Most striking was the lack of awareness of the primary physician towards the possibility of a malignant lesion. In 2/3 patients, no pre-operative imaging studies were performed. Only two patients (13%) had a magnetic resonance imaging (MRI) scan. Fine needle biopsy was unsuccessfully performed in three patients (20%). Resection margins were intralesional in 11 (73%) and marginal in 4 patients. Nine lesions (60%) were high-grade lesions with MFH being the most frequent sarcoma ($n = 4$). Surgical oncologic rules were disregarded in six cases including a transverse incision, contamination through wrongly placed drains and the opening of probably uninvolved joints. Postoperative magnetic resonance imaging (MRI) scans showed a poor negative predictive value for residual tumor. Repeated resection was performed in all patients including amputation in three patients. In 10 patients (67%), residual tumor was found. Four patients received adjuvant local radiation, with additional chemotherapy in two of them. Follow-up was obtained at an average of 4.1 years (range 7–114 months). Four patients (27%) developed metastasizing disease with a local recurrence in three of them (20%). One patient (7%) died due to tumor progression. Inadequate primary excision of a soft tissue sarcoma leads to a high local recurrence rate, more mutilating surgery and obscures the long-term prognosis. Physicians' alertness towards the possible malignant origin of an enlarging soft tissue mass can not be overemphasized. Definitive surgical treatment including biopsy belongs in the hands of one oncologically trained surgeon who can reliably perform wide resection and plan the necessary reconstructive procedure.

**Comparative genomic hybridization and representational difference analysis as tools to identify and molecularly clone tumor-specific chromosomal anomalies**

A. Simons,[1] I. Janssen,[1] R. F. Suijkerbuijk,[1] A. Forus[2] & A. Geurts van Kessel[1]

([1]*Department of Human Genetics, University Hospital Nijmegen, The Netherlands &* [2]*Department of Tumor Biology, Norwegian Radium Hospital, Oslo, Norway*)

Gains and losses of chromosomal sequences within a tumor genome can be detected in a single-step *in situ* hybridization procedure following the comparative genomic hybridization (CGH) strategy. Previously, we have used this technique to define novel genomic imbalances (i.e. amplifications) in human osteosarcomas and soft tissue sarcomas of various histologic (sub)types. In order to isolate these amplified sequences, we have now extended these CGH analyses by employing the recently developed representational difference analysis (RDA) technique. RDA allows for the direct isolation of differences between tumor DNA and normal DNA through the combination of subtractive hybridization and polymerase chain reaction (PCR) amplification processes. An osteosarcoma showing clear amplifications of genomic regions on chromosomes 17 (p11.2) and 19 (q13.1) by CGH was subjected to RDA. Using tumor DNA as tester and a mixture of DNAs from five normal individuals as driver, several DNA fragments were obtained after three rounds of subtraction and amplification. Southern blot analysis revealed the amplification of some of these RDA fragments in the tumor genome. Subsequent somatic cell hybrid analysis disclosed their chromosome 19 origin. FISH analysis using a YAC isolated with one of the chromosome 19-specific RDA fragments on normal human chromosomes and on tumor nuclei confirmed its chromosomal localization (i.e. 19q13.1) and its amplification in the tumor, respectively. These results demonstrate that CGH and RDA appear to be useful tools for identifying and molecularly cloning tumor-amplified sequences.

**Early results of pegylated-liposomal doxorubicin (Doxil) in refractory sarcoma**

K. M. Skubitz
(*University of Minnesota Medical School, Minneapolis, MN 55455, USA*)

Pegylated-liposomal doxorubicin (Doxil) is a unique form of liposomal doxorubicin in which the liposomes are coated with polyethylene glycol. The polyethylene glycol confers useful properties including a diminished uptake by the reticuloendothelial system, leading to a much longer half-life in blood (~50–60 h) and a different toxicity profile from non-pegylated liposomes. We have begun a phase II study of Doxil in refractory sarcoma. The patient population consists of patients with sarcomas that have failed doxorubicin, DTIC, ifosfamide, VP-16 and, in some cases, additional chemotherapy. The initial dose per course is 55 mg $m^{-2}$. Courses are repeated every 4 weeks or longer. Dose modification based on mucositis and hand–foot syndrome (the main limiting toxicities) is performed following an algorithm that modifies both the dose and interval between treatments. Treatment was generally well tolerated. Of the first 22 evaluable patients, 7 had stable disease for 6, 3, 3, 2+, 3+, 8+ and 8+ months, 12 patients had progressive disease, 1 a mixed response and 1 a partial response (maintained for 8+ months).

One patient exhibited a more complex response; her tumor was growing rapidly, the tumor volume increased after three treatments but appeared more cystic suggesting necrosis. No further treatment was given and she was observed. Four months after stopping treatment computed tomography scans revealed that the tumor had decreased markedly in size. Two months later scans revealed the tumor had begun to grow again and treatment was reinstated. These data suggest that pegylated-liposomal doxorubicin has significant activity in refractory sarcoma and is appropriate for evaluation in earlier stage disease.

**Clinical and molecular characterization of a familial desmoid syndrome**

A. M. Yahanda, E. R. Fearon, K. A. Taylor, M. W. Mulholland & V. K. Sondak
*(University of Michigan Medical School, Ann Arbor, MI 48109, USA)*

Familial desmoid syndromes are exceedingly rare, although desmoid tumors are well recognized as secondary manifestations of classic familial adenomatous polyposis (FAP). We report a kindred of six women spanning two generations all with desmoid tumors in the absence of classic features of FAP. The desmoids were generally multiple, locally recurrent and in most cases occurred at a young age. The majority of the tumors were in the superficial tissues of the abdomen, chest wall, breast or axilla and occurred at sites with no known prior trauma or surgery. In one member of the kindred, a desmoid was located in the retroperitoneum in a site of two previous surgeries. Further evaluation of this family with endoscopy demonstrated that all members with desmoids and one obligate carrier without desmoids had adenomas of the duodenum and periampullary region, as well as innumerable fundic gland polyps of the stomach. Several of the members were found to have one or two adenomas of the colon, while others were found to have none. These findings suggested that this family might represent an attenuated form of FAP. FAP is known to result from mutations in the adenomatous polyposis coli (APC) gene. To establish possible linkage of familial desmoid disease with the APC gene locus, haplotype analysis was performed using highly polymorphic microsatellite markers flanking or within the gene. All members of this family affected with desmoid tumors were found to have a specific haplotype, implicating APC as the responsible gene. Mutational analysis was then performed using both heteroduplex analysis and direct sequencing, and a chain terminating mutation was identified at codon 2504 of the APC gene. This mutation destroys a *Fokl* restriction site, allowing rapid screening for the mutation within the family. All affected members and an unaffected obligate carrier demonstrated a restriction pattern consistent with the loss of this *Fokl* site. Evaluation of 60 normal alleles showed only the expected wild-type pattern. These studies demonstrate that this familial desmoid syndrome represents a hitherto unrecognized phenotypic variant of FAP. The mutation at codon 2504, near the 3′ end of the APC gene, preserves the $\beta$-catenin binding domain, the loss of which is implicated in the genesis of colonic polyposis and cancer. The mechanism by which mutations 3′ to the $\beta$-catenin binding domain still predispose affected individuals to desmoid tumors as well as adenomas and polyps of the stomach and duodenum is unknown, but merits further study. Understanding the pathogenesis of desmoid tumors in this kindred could provide new insights and even treatments for sporadic desmoids as well.

**Pattern of relapse in extremity soft tissue sarcomas and impact on survival of palliative treatment**

S. Frustaci,[1] P. Stefanovski,[1] A. De Paoli,[1] F. Gherlinzoni,[2] H. Zmerly,[2] A. Comandone,[3] A. Buonadonna,[1] P. Olmi,[3] V. Ippolito,[3] G. Apice[3] & P. Picci[2]

*(From the [1]Aviano, [2]Rizzoli & [3]collaborating institutions)*

The natural history of extremity, high-grade soft tissue sarcomas (STS) is known. The majority of patients develop distant metastases, mainly at the lung, as a first site of relapse 2–3 years after diagnosis. Lung relapse ultimately represents the cause of death in all patients, determining a median survival of $\approx 12$ months. However, more aggressive approaches in the adjuvant and palliative setting may have partially changed this scenario. The follow-up of patients entered in a prospective randomized adjuvant versus control trial provides on opportunity to re-evaluate the pattern of relapse and verify whether a palliative aggressive approach ($CT + CH \pm CT$) can increase post-metastasis survival. From June 1992 to November 1996, 104 patients affected by extremity, high-grade, $> 5$ cm, deep-seated STS were randomized to adjuvant chemotherapy (CT) or follow-up. Specific sites of first relapse and successive relapses as of May 1997 were as shown in the table.

| Relapses | First | | | Second | | | Third | | | Total |
|---|---|---|---|---|---|---|---|---|---|---|
| | CT | FU | Total | CT | FU | Total | CT | FU | Total | |
| Local | 2 | 5 | 7 | 1 | 1 | 2 | | | | 9 |
| Local and distant | | 2 | 2 | | | | | | | 2 |
| Distant | | | 38 | | | 5 | | | 4 | 47 |
| Lung | 15 | 17 | 32 | 1 | | 1 | | | | 32 |
| Lymphnodal | | 2 | 2 | | 1 | 1 | | | | 3 |
| Visceral | 1 | | 1 | | | | | | | 1 |
| Brain | | | | | 2 | 2 | | | | 2 |
| Other | 1 | 2 | 3 | | 1 | 1 | 2 | 2 | 4 | 8 |
| Total | 19 | 28 | 47 | | | 7 | | | 4 | 58 |

Treatment of 32 patients with exclusive lung metastases was (1) chemotherapy + surgery in 7 patients, (2) surgery in 6, (3) exclusive CT in 12 and (4) none in 8, respectively. Chemotherapy consisted of an ADM-IFO-based combination in all patients. The overall response rate was 54.5% (6 of 11 evaluable patients). The median post-metastasis survival was 102, 103 and 71 weeks for the three groups, respectively. Even though selection factors may have influenced these figures, we conclude that an aggressive approach with intensified CT and/or surgical resection of lung metastases should be attempted whenever possible in first relapse patients.

### Experimental trial on *in vivo* visualization of mouse osteosarcoma with acridine orange

T. Suginoshita,[1] K. Kusuzaki,[1] S. Hashiguchi,[1] M. Hirata,[1] G. Minami,[1] Y. Hirasawa[1] & T. Ashihara[2]

*([1]Departments of Orthopaedic Surgery & [2]Pathology, Kyoto Prefectural University of Medicine, Kyoto, Japan)*

*Introduction.* If the malignant tumor is visible during surgery, complete tumor resection with minimal damage to intact tissues is possible. Such a tumor resection provides excellent limb function in patients with malignant bone and soft tissue tumors. In this study, we attempted to visualize mouse osteosarcoma using acridine orange (AO) which is one of the vital fluorescence dyes.
*Materials and methods.* Radiation-induced MGH mouse osteosarcoma cell line (MOS) was inoculated into C3H mice (4-weeks-old, male) subcutaneously. After macroscopic tumor formation, each mouse received an intraperitoneal injection of 0.5, 1.0, 1.5, 2.0 2.5 $mg\,kg^{-1}$ AO. The mice were illuminated with a blue excitation light which was selected from a halogen lamp source at 1, 2, 3, 24 h and 1 week after injection. Differences in fluorescence intensities between the tumor and surrounding normal tissues such as muscle, skin or fat was evaluated qualitatively under a low-power fluorescence microscope. Pulmonary metastatic lesions were also detected.
*Results.* In the high-dose AO group (1.5–2.5 $mg\,kg^{-1}$), both tumor and soft tissues emitted intensive fluorescence, while in the low-dose AO group, both emitted weak fluorescence. In these two groups, it was difficult to distinguish the tumor from normal soft tissues under a low-power fluorescence microscope. However, the difference in qualitative fluorescence intensities between the tumor and normal soft tissues was most evident in the group at 2 or 3 h after injection with 1.0 $mg\,kg^{-1}$ AO. At 1 h or at 24 h and 1 week after injection with 1.0 $mg\,kg^{-1}$ AO, fluorescence contrast between the tumor and soft tissues was unclear. In pulmonary metastatic lesions, even small lesions, less than 1 mm in diameter, were distinguishable from normal lung tissue at 2 h after injection with 1.0 $mg\,kg^{-1}$ AO.
*Conclusions.* These results suggest that AO may be useful in distinguishing tumor from surrounding intact soft tissues under illumination with blue light at 2 or 3 h after intraperitoneal injection with 1.0 $mg\,kg^{-1}$. We hope that this idea will be applicable to human malignant bone and soft tissue tumors.

### Hepatic artery infusion with doxorubicin and CDDP for liver metastases of angiosarcoma: a case report

P. Reichardt, J. Tilgner, S. Bender, E. Fischer-Funk & B. Dörken
*(Department of Hematology, Oncölogy and Tumorimmunology, Robert-Rössle-Klinik, Virchow-Klinikum, Humboldt University, D-13122 Berlin, Germany)*

Angiosarcoma of the breast is a very rare and highly aggressive disease that carries a very bad prognosis. We report the case of a 46-year-old female with a history of angiosarcoma of the right breast that had been removed by mastectomy in August 1995. One month later, angiosarcoma of the left breast was diagnosed and again a simple mastectomy was performed. Due to infiltration into the thoracic wall, the patient received three cycles of post-operative chemotherapy with epirubicin and ifosfamide until January 1996. In November 1996, a single liver metastasis was removed by hemihepatectomy. In March 1997, the patient presented to our hospital with multiple liver metastases and massive ascites. Since liver metastases of soft tissue sarcomas rarely respond to systemic chemotherapy, we performed hepatic artery infusion (HAI) with CDDP 100 $mg\,m^{-2}$ over 2 h, followed by doxorubicin 30 $mg\,m^{-2}$ given by bolus injection. Therefore, a 5 French catheter was introduced in the femoral artery and finally placed in the A. hepatica propria. After chemotherapy, the catheter was removed and the patient was discharged 2 days later without any complaints or laboratory abnormalities related to the procedure. Ten days later the patient presented with grade 4 myelotoxicity but without any ascites. Magnetic resonance imaging (MRI) showed regression in the count and size of the multiple liver metastases. HAI was repeated on day 26 after initial treatment with identical procedure and chemotherapy doses. After 7 weeks, MRI showed progression of the liver metastases. The patient was put on systemic ifosfamide, that had to be stopped due to severe toxocity after a total dose of 6 $g\,m^{-2}$. Today, the patient is alive 14 weeks after the first HAI. Despite the rather short remission duration, this case suggests a potential for intra-arterial chemotherapy for liver metastases of soft tissue sarcoma in selected cases.

### Importance of a multi-disciplinary approach in the treatment of advanced and/or metastatic soft tissue sarcomas: experience of the national Cancer Institute of Genoa

S. Toma, G. Canavese, A. Grimaldi, L. Moresco, R. Palumbo, P. Percivale, P. Raffo, C. Vecchio, G. Villani & F. Badellino
*(National Institute for Cancer Research, I-16132, Genova, Italy)*

Curative-intent surgery remains the main goal in the multi-disciplinary approach to soft tissue sarcoma (STS). In advanced disease also, the surgical approach is to be pursued in those patients which can be rendered disease free. In view of this endpoint, different arms such as chemotherapy (CT) and radiotherapy (RT) can be used alone or in combination, to allow further radical surgery. Here, we review the results of a sequential phase II study performed at our institution on patients with advanced and/or metastatic STS with: (1) systemic poli-CT and (2) combined CT/RT treatment. (1) Ninety advanced and/or metastatic STS patients were treated with epirubicin (at escalating doses from 50 to 120 $mg/m^2$/course) combined with ifosfamide (10 $g/m^2$/course). ORs were 57% (13 complete response (CR) and 39 partial response (PR)), with a clear dose–response effect for epirubicin. Lung metastasectomy was performed in 14 responder patients (15%). The overall median survival time was found to be positively correlated with the achievement of ORs: 10 months for all evaluable patients versus 21 months in complete or partial responders versus 31 months in patients who underwent surgery. (2) The therapeutic ratio of a concomitant CT/RT schedule was investigated in 58 patients with locally advanced, inoperable and/or metastatic STS. Doxorubicin was administered by portable infusion pumps at a dose of 12 $mg/m^2$/day over

5 consecutive days, concomitantly with RT (150 or 200 cGy/day, for trunk or extremity lesions, respectively, days 1–5); cycles were repeated every 2–3 weeks in an ambulatory regimen. ORs were 58%, with 3 CR and 32 PR. After 2–4 cycles of treatment, 19 responder patients (54%) underwent surgery, which was radical in all but one case. At a median follow-up of 36 months (range 15–96 months) median survival was 15 months in the whole group (range 2–61 months) and 22 months in responders, reaching 38 months in patients who had undergone radical surgery. The ORs were achieved with low median dose of both doxorubicin and RT (180 mg/m$^2$ and 3.000 cGy, respectively). Also in this population the achievement of an OR had a predictive value for overall survival that was statistically significant. From our experience, it emerged that the main prognostic factor in such populations is probably whether radical surgery is possible, considering that overall survival time had approximately tripled in patients undergoing surgery compared to those who did not. The choice of optimal treatment in advanced STS patients primarily depends on the achievable goals in the individual patient's therapy plan. Thus, the combined CT/RT schedule might be an optimal approach where the loco-regional disease is to be privileged, also deserving further investigation in less advanced stage of disease (neoadjuvant setting). The schedule's good activity with almost absent toxicity, as well as the possibility of limb-sparing surgery emerged as significant issues. However, more aggressive systemic CT with correlated increased toxicity should be considered in those patients who might ultimately benefit from radical surgery (i.e. STS patients with lung metastases).

### Mitotic count, proliferation and apoptosis in soft tissue sarcomas in relation to treatment response to epirubicin, vindesine and ifosfamide

H. H. T. Luu, W. T. A. van der Graaf, B. E. C. Plaat, M. F. Mastik, H. J. Hoekstra & W. M. Molenaar
(*Departments of Pathology, Medical Oncology and Surgical Oncology, University of Groningen, Groningen, The Netherlands*)

*Introduction.* Recent studies suggest a relation between treatment response to chemotherapy and mitotic count, proliferation and/or the amount of apoptosis in lymphomas and carcinomas. The aim of the present study was to relate these parameters to the response to chemotherapy in patients with metastatic soft tissue sarcoma (STS) treated with EVI (epirubicin, vindesine and ifosfamide). Non-responders to EVI (i.e. progressive and stable disease) were compared to responders (i.e. partial or complete response) regarding the amount of mitoses, proliferation and apoptosis in a tumor presentation $< 6$ months before treatment with EVI.
*Methods.* On archival material of 14 tumors, i.e. 7 (recurrent) primary tumors and 7 metastases from 23 evaluable STS patients who were treated with EVI, mitoses were counted per 2 mm$^2$ in H&E stained sections. Proliferation was determined using immunohistologic staining for the proliferation associated Ki-67 antigen (MIB1). Apoptosis was visualized by enzymatic detection of DNA fragmentation (TUNEL-method).
*Results.* Although not significant ($p > 0.05$), differences between responders and non-responders were observed in number of mitoses, proliferation and apoptosis:

| TUMpreEVI | Non-responders | Responders |
|---|---|---|
| Mean number of mitoses (per 2 mm$^2$) | $9 \pm 5.3$ | $6 \pm 5.5$ |
| Mean proliferation (%) | $26 \pm 7.8$ | $39 \pm 24.0$ |
| Mean apoptosis (%) | $0.60 \pm 0.55$ | $0.27 \pm 0.26$ |

*Conclusions.* These data from tumor material obtained $< 6$ months before the treatment with EVI of this relatively small number of patients with distant metastases may indicate that a good response to EVI is associated with a high proliferative activity and a low apoptotic index.

### The use of paediatric chemotherapy protocols at full dose is both a rational and feasible treatment strategy in adults with ewing's family tumours

M. W. Verrill, I. R. Judson, E. Wiltshaw, J. M. Thomas, C. L. Harmer & C. Fisher
(*Sarcoma Unit, Royal Marsden NHS Trust, London SW3 6JJ, UK*)

Ewing's sarcoma (ES) and PNET are rare, limiting opportunities for therapy studies in adults. Chemotherapy regimens adapted from paediatric studies are often used for adults but concerns about poor outcome and treatment toxicity may adversely affect drug dose intensity. Thirty-four patients with ES/PNET at our institution have received dose-intensive chemotherapy comprising ifosfamide 9 g m$^{-2}$ (first four cycles) or 6 g m$^{-2}$, doxorubicin 60 mg m$^{-2}$ and vincristine 2 mg (IVAD) given 3 weekly and we have reviewed received drug dose intensity (RDI), toxicity and survival. Patient characteristics were as follows, median age 23 years; male 21, female 13; previously treated 1; bone 13, soft tissue 21; tumour burden, localized $< 100$ ml 11, localized $> 100$ ml 13, metastatic 10. RDI in 30 evaluable patients was 0.92 compared to the standard IVAD schedule. Myelosuppression was the major toxicity; 83% of patients experienced grade 4 neutropenia. There was no major renal or cardiac toxicity. In patients without metastases at presentation, 5-year overall survival was 63% and progression-free survival was 39%. Tumour burden at presentation was statistically significantly associated with survival ($p = 0.002$). The 5-year survival rate of 80% in patients presenting with low volume non-metastatic disease was equivalent to published paediatric series. Although the IVAD regimens are myelotoxic in adults, they can be given safely. We recommend that adults with ES/PNET should be included in current multi-centre, multi-disciplinary treatment studies directed at children.

### Susceptibility of fibromatosis cells in short-term culture to ifosfamide: a possible new approach to the treatment of clinically aggressive cases

M. W. Verrill,[1,2] H. M. Coley,[1] I. R. Judson[1,2] & C. O. Fisher[2]
([1]*CRC Centre for Cancer Therapeutics, Institute Cancer Research, Sutton SM2 5NG &* [2]*Sarcoma Unit, Royal Marsden NHS Trust, London SW3 6JJ, UK*)

Deep fibromatoses are large, often rapidly growing but benign soft tissue tumours. Although surgery is the main stay of treatment, in unremitting and aggressive cases the use of cytotoxic chemotherapy may produce objective tumour responses. Fresh tumour samples from four patients with fibromatosis were investigated as part of a study of drug resistance in soft tissue tumours. Following short-term culture of fibromatosis cells *in vitro*, chemosensitivity to 4-hydroperoxy-ifosfamide (IF), the active form of ifosfamide, and doxorubicin (DOX) was tested. Following 72-h continuous exposure to each drug, surviving cell fraction was assessed using the LDH assay. Mean $IC_{50}$ values for IF and DOX were 6.2 and 0.35 $\mu mol\ l^{-1}$ respectively. In samples of soft tissue sarcoma (STS) from the same study the mean $IC_{50}$ values for IF and DOX were 14.8 and 1.69 $\mu$mol. The difference in mean IF $IC_{50}$ values for fibromatosis and STS samples was statistically significant. We are not aware of any other report suggesting the use of IF in this condition. These observations suggest that, for patients with inoperable or progressive lesions of fibromatosis causing significant morbidity, IF may prove to be a valuable treatment option.

### Prognosis in soft tissue sarcomas

S. Vraa, J. Keller, O. S. Nielsen, A. G. Jurik, O. Sneppen & O. M. Jensen
(*The Sarcoma Center, Aarhus University Hospital, Aarhus, Denmark*)

*Purpose.* The purpose of the study was to evaluate the outcome and possible prognostic factors of patients with soft tissue sarcoma (STS) after surgical treatment.
*Patients and methods.* From January 1979 to July 1993, 316 patients with STS in the extremities or truncus, without distant metastasis were treated surgically at the Sarcoma Center in Aarhus. The median age was 56 years range (1–94 years). Fifty-two patients (16%) had a low-grade tumor, 60 (19%) an intermediate and 204 (65%) a high-grade tumor. Fifty-nine patients (19%) had tumor localized in the upper extremity, 163 (52%) in the lower extremity and 94 patients (30%) presented with tumor in the truncus, including shoulder and buttock lesions. One-hundred and ninety-eight tumors (63%) extended in one anatomical compartment only while 118 tumors (37%) spread into two ore more compartments. Sixty-seven patients (21%) underwent amputation and 249 patients (79%) had a local resection. Fifty patients (16%) received adjuvant radiation therapy and 13 patients (4%) received adjuvant chemotherapy. The minimum follow-up time was 46 months. For the statistical analysis the log-rank test and Cox's proportional hazard model were used.
*Results.* Local recurrence developed in 59 patients (19%) and the actuarial local relapse rate was 18% at 5 years. Prognostic factors for local control were surgical margin, adjuvant radiation therapy, histological grade and compartmental location of the tumor. Five- and 10-year survival rates were 75 and 67%, respectively. Prognostic factors for survival were age, size of tumor, lower extremity location, histological grade and compartmental location of the tumor.
*Conclusions.* Histopathological grade and anatomical extension were the most important factors for local control and survival in STS.

### Application of comparative genomic hybridization in the analysis of genomic changes in malignant fibrous histiocytomas

V. M. Weibolt,[1] A. Forus,[2] A. Simons,[1] J. Bridge,[3] A. Geurts van Kessel[1] & R. F. Suijkerbuijk[1]

(*[1]Department of Human Genetics, University Hospital Nijmegen, The Netherlands, [2]Department of Tumor Biology, The Radium Hospital, Oslo, Norway & [3]Department of Pathology/Human Genetics, University of Nebraska, Omaha, USA*)

*Background.* Malignant fibrous histiocytomas (MFHs) form a heterogeneous group of sarcomas that are the most common malignant mesenchymal tumors in adults. Even though there are numerous studies describing the clinical, histochemical, ultrastructural and tissue culture properties, the histogenesis of this neoplasm remains controversial. So far, cytogenetic studies showed high karyotype complexity including double minutes, ring chromosomes and various other marker chromosomes. The former two chromosomal anomalies are indicative of gene amplifications.
*Objective.* To identify consistent unbalanced chromosomal abnormalities that somehow are correlated with tumor initiation and/or progression in relation to the different histological subtypes using comparative genomic hybridization. Such abnormalities have proven to be indicative for the involvement of tumor-related (tumor supressor and onco-) genes.
*Method.* Comparative genomic *in situ* hybridization (CGH) is a molecular cytogenetic method capable of screening whole genomes for over- and under-representation of DNA sequences and mapping them directly to their position on normal chromosomes. Tumor DNAs extracted from fresh, frozen or formalin-fixed paraffin-embedded tissue can be used, thus eliminating the need for tumor tissue culturing. CGH experiments are carried out using a 1:1 mixture of digoxigenin-labeled tumor DNA and biotinylated normal (control) DNA, hybridized on to normal metaphase spreads. Hybridization is visualized with two spectrally different fluorochromes. Fluorochrome intensities are measured, calculated and compared along the axis of the chromosomes in order to create a CGH ratio profile of those chromosomes using specifically designed CGH software. Significant changes in the CGH ratio for a certain chromosomal region, resulting in values above or below the statistically determined thresholds, reflect gain (amplification) or loss (deletion) of that region. As such, CGH may pinpoint to chromosomal sites that are likely to harbor oncogenes or tumor suppressor genes.
*Results.* In our study, we have performed CGH analysis on DNA subtracted from approximately 50 soft tissue and bone MFHs to gain insight into the significance of genomic imbalances in relation to the histopathological features such as subtype, tumor grading and cytogenetic findings. In the 28 samples analyzed so far, gains and/or deletions concerning whole chromosomes or chromosome regions have been found in 26 cases. The most frequently over-represented regions are 1p21–22 (10 cases), 1q21-q22/23 (12 cases), 6q14–23 and chromosome 19. The under-represented regions are 1p33–35, 9p (13 cases), and 10q25–26 (14 cases). These patterns may be useful in establishing diagnosis, therapy and clinical behavior. A detailed overview of the results on the CGH analysis of all cases will be presented and discussed.

### Phase II study of temozolomide in adult patients with advanced soft tissue sarcoma

P. J. Woll, I. Judson, S. M. Lee, S. Leyvraz, S. Rodenhuis, O. S. Nielsen, J. M. Buesa, P. C. Lorigan, C. Hermans, M. van Glabbeke & J. Verweij
(*EORTC Soft Tissue and Bone Sarcoma Group, Brussels, Belgium*)

Dacarbazine is one of the active cytotoxics for soft tissue sarcoma (STS). It undergoes N-demethylation in the liver to the active derivative, MTIC. Temozolomide is an MTIC pro-drug which does not require metabolic activation and is orally bioavailable. It has broad-spectrum antitumour activity in experimental models. In preclinical studies, its activity was schedule-dependent. In phase I

and II studies, it has shown promising activity against metastatic melanoma and primary brain tumours when administered at 150 mg $m^{-2}$ daily for 5 consecutive days every 4 weeks, then 200 mg $m^{-2} \times 5$ days 4 weeks if no myelosuppression worse than CTC grade 2 was observed in the first cycle. We tested temozolomide for activity in advanced STS using this schedule. Thirty-two patients were enrolled with locally advanced or metastatic STS progressive after one combination or two single agent regimens. One patient was not pretreated. Data are available for 31 patients: 15 male, 16 female, median age 50 years (range 27–73 years). Leiomyosarcoma was the most common pathology (32%). Ninety-seven treatment cycles were given (median 2, range 0–10). One partial tumour response was seen and 10 patients had stable disease for at least 8 weeks. Twenty-four stopped treatment because of disease progression and two because of toxicity. The treatment was well tolerated. Nausea (26 patients) and vomiting (17 patients) were common but were only severe (CTC grade 3/4) in 4 and 5 patients, respectively. Grade 2 leukopenia occurred in three patients and grade 3 thrombocytopenia in one. Temozolomide cannot be recommended for further study in advanced STS at this dose and schedule.

### Infrequent mutation of the *p16/MTS1* gene and over-expression of cyclin-dependent kinase-4 in human primary soft tissue sarcoma

J. Yao, R. E. Pollock, A. Lang, M. Tan, P. Pisters, D. Goodrich, A. El-Naggar & D. Yu
(*Department of Surgical Oncology, Department of Tumor Biology, Department of Pathology, University of Texas, MD Anderson Cancer Center, Houston, TX 77030, USA*)

The $p16^{INK4a}$/MTS1 (p16) gene encodes a specific inhibitor of cyclin-dependent kinase (CDK)4 and CDK6. The p16 gene is frequently mutated or deleted in many types of cancer cell lines as well as in certain types of primary tumors. p16 knockout mice are viable but predisposed to sarcoma and B-cell lymphoma. To investigate the role of p16 in human soft tissue sarcoma tumor progression, we examined the p16 gene by Southern blot analysis and polymerase chain reaction. (PCR) sequencing in 30 pairs of primary soft tissue sarcomas and autologous normal tissue. Only one tumor sample showed possible rearrangement of the p16 gene. In contrast, Western blot analysis of the p16 protein in 20 pairs of samples showed decreased p16 expression in only 20% of the tumors but elevated p16 expression in 40% of the tumors when compared with the normal controls. The over-expression of p16 was not concomitant with the loss of the RB protein as is found in several other types of cancers, since more than half the tumors with increased p16 expression also had high levels of RB protein. However, the p16 target protein CDK4 was over-expressed in at least 60% of the tumors. In the majority of cases, CDK4 over-expression accompanied elevated p16 and/or RB levels. Our results suggest that: (a) alteration of the p16 gene is infrequent in primary soft tissue sarcoma, (b) CDK4 may act as an oncogene in soft tissue sarcoma, and (c) elevated p16 and RB levels might be the result of compensatory upregulation of these proteins to counteract CDK4 over-expression in these tumors. Our results also suggest that it is more informative to examine aberrations in the 'p16–CDK4/cyclin D–RB' pathway than to examine selectively individual components in this pathway when investigating genetic changes involved in human malignancy.

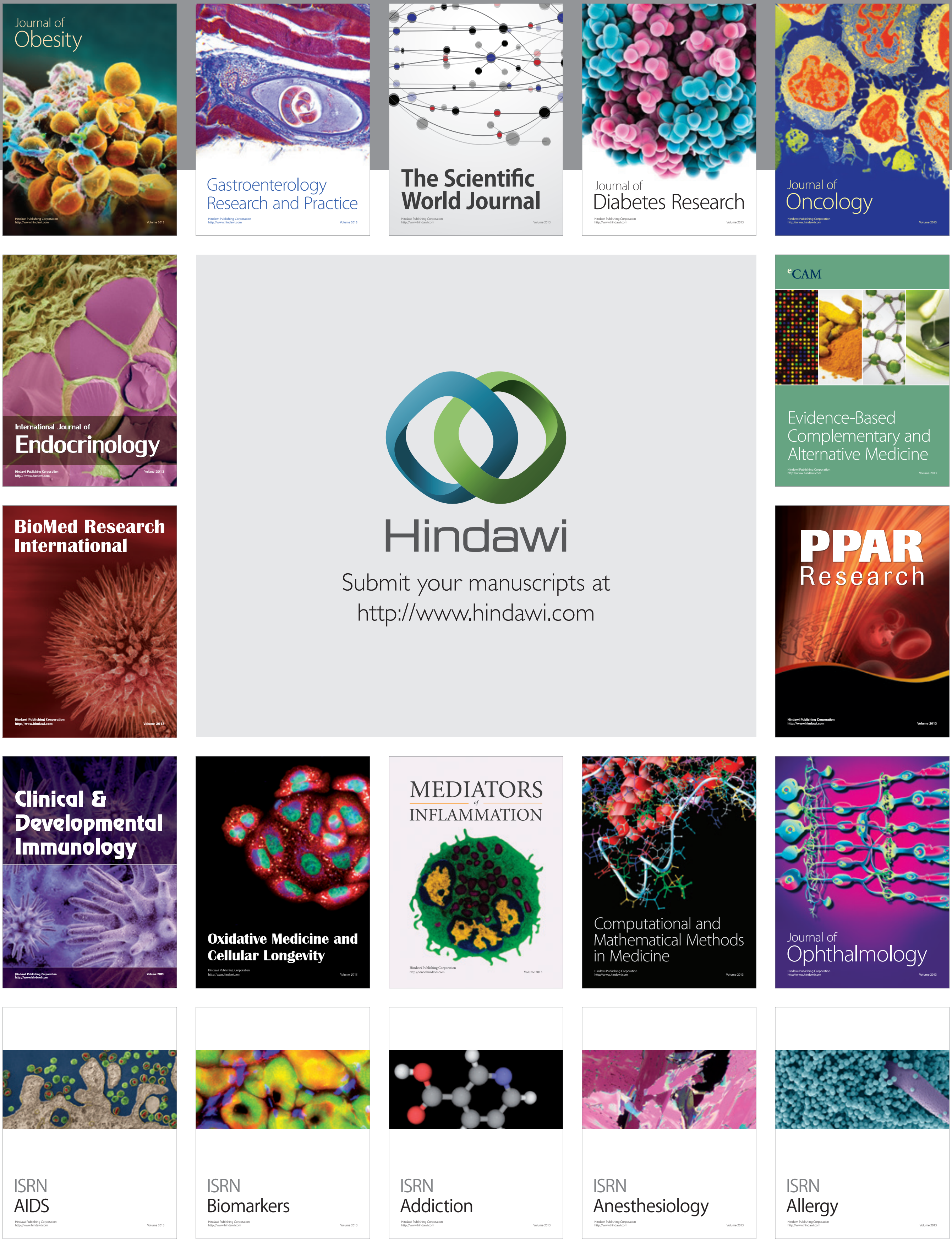

Journal of Obesity

Gastroenterology Research and Practice

The Scientific World Journal

Journal of Diabetes Research

Journal of Oncology

International Journal of Endocrinology

Hindawi

Submit your manuscripts at http://www.hindawi.com eCAM

Evidence-Based Complementary and Alternative Medicine

BioMed Research International

PPAR Research

Clinical & Developmental Immunology

Oxidative Medicine and Cellular Longevity

MEDIATORS of INFLAMMATION

Computational and Mathematical Methods in Medicine

Journal of Ophthalmology

ISRN AIDS

ISRN Biomarkers

ISRN Addiction

ISRN Anesthesiology

ISRN Allergy

